# Cell autonomous regional differences in oligodendrocyte lineage development and responses to oncohistone H3.3 K27M

**DOI:** 10.1038/s41467-026-76468-6

**Published:** 2026-08-26

**Authors:** Jared M. Andrews, Kaitlin M. Budd, Chang-Hyuk Kwon, Jon D. Larson, Abbas Shirinifard, Lawryn H. Kasper, Chanrika C. Williams, Alfonso Lavado, Sharon King, Jorge Gutierrez, Daniel Stabley, Tong Lin, Sara A. Lewis, Paul A. Northcott, Arzu Onar-Thomas, Suzanne J. Baker

**Affiliations:** 1 Department of Developmental Neurobiology, St. Jude Children’s Research Hospital, Memphis, TN USA; 2 Center of Excellence in Neuro-Oncology Sciences, St. Jude Children’s Research Hospital, Memphis, TN USA; 3 St. Jude Graduate School of Biomedical Sciences, St. Jude Children’s Research Hospital, Memphis, TN USA; 4 Center for Pediatric Neurological Disease Research, St. Jude Children’s Research Hospital, Memphis, TN USA; 5 Department of Cell and Molecular Biology, St. Jude Children’s Research Hospital, Memphis, TN USA; 6 Information Services, St. Jude Children’s Research Hospital, Memphis, TN USA; 7 Department of Biostatistics, St. Jude Children’s Research Hospital, Memphis, TN USA

**Keywords:** Differentiation, CNS cancer, Paediatric cancer, Glial biology, Epigenetic memory

## Abstract

Diffuse midline glioma, H3K27-altered (DMG), is a lethal midline brain tumor. Most DMG, unlike glioma arising in other regions, harbor histone H3.3 K27M (K27M) mutations. The basis for this anatomical selectivity remains unclear. Stem-like DMG cell transcriptomes most resemble oligodendrocyte precursor cells (OPCs). Using conditional K27M knock-in mice, we show that K27M reduces oligodendrocyte differentiation, altering the proportions of oligodendrocytic cell states in a region-specific manner. In vivo EdU labeling in tissue-cleared whole brains revealed greater K27M-driven increases in proliferation within pons, midline, and hindbrain regions than in telencephalon. In vitro, wild-type brainstem OPCs proliferate more slowly and differentiate later than telencephalic OPCs. K27M enhances brainstem OPC proliferation and disrupts regional transcriptional programs, selectively restraining full maturation of brainstem OPCs while inducing brainstem-selective upregulation of bivalent Bmp, Wnt, and Notch pathway genes. These findings suggest that K27M exploits intrinsic regional differences in oligodendrocyte development, creating a brainstem-selective window for gliomagenesis.

## Abstract (web summary)

Here authors report that histone H3.3 K27M mutations drive diffuse midline glioma by ablating intrinsic regional differences in oligodendrocyte development, selectively expanding immature oligodendrocyte precursor cell populations in the brainstem while disrupting normal differentiation programs.

## Introduction

Diffuse midline glioma (DMG), H3K27-altered, is a lethal disease arising in midline brain structures, most commonly in the pons. This subgroup of tumors is uniformly characterized by a dramatic reduction in the epigenetic modification H3K27me3, usually driven by somatic point mutations substituting lysine 27 with methionine (K27M) in histone H3 variants (DMG H3 K27M), predominantly in H3.3^1,2^. K27M dominantly inhibits PRC2 activity to broadly decrease H3K27me3 when expressed in diverse cell types derived from different brain regions and tissues, but in human disease contexts, K27M mutations are highly specific for DMG^3^.

Clonal representation of the K27M mutation in DMG patient tumors and analyses of tumor evolution in DMG H3 K27M patients show that K27M is an early or initiating event in tumorigenesis^4–7^. Although K27M alone is insufficient to drive tumorigenesis in model systems, multiple models have shown that K27M cooperates with additional gliomagenic alterations to increase or hasten tumorigenesis^8–15^. Utilizing inducible genetically engineered mouse models, we previously showed that widespread expression of activated PDGFRA and deletion of Tp53 drove high-grade gliomas with broad anatomical distribution. Combining these mutations with H3.3 K27M selectively accelerated gliomagenesis and increased the proportion of brainstem tumors^10^.

How K27M may alter development and specifically predispose midline and brainstem glia to DMG pathogenesis remains largely unaddressed. Introducing H3K27M with other gliomagenic mutations in neural stem cells or glial progenitors of model systems gives rise to glioma^8–12,14^. However, the temporal incidence of DMG and the super-enhancer landscape of DMG support oligodendrocyte progenitor cells (OPC) as likely DMG candidate cells of origin^16–20^. Furthermore, most cells within patient DMG K27M tumors resemble a highly proliferative OPC-like state^18,21,22^. Knockdown or knockout of K27M in DMG H3 K27M tumor cells reduces proliferation and induces glial differentiation signatures^23,24^, suggesting that K27M plays a role in maintenance of the proliferative OPC-like state, thus connecting DMG pathogenesis to oligodendrocyte development.

Axon ensheathment by myelinating oligodendrocytes is essential for rapid action potential propagation, axonal health, and metabolic support of axons throughout the brain; however, the developmental timing and physiological consequences of myelination varies by brain region. Forebrain OPCs arise in waves, initially from the embryonic ganglionic eminences and subsequently from forebrain progenitors that ultimately generate most postnatal OPCs. However, transcriptomes of OPCs arising at different stages converged into similar transcriptional states when evaluated in the mouse at P7^25^. Myelination occurs first in the brainstem and proceeds from caudal to rostral, with cortical developmental myelination and adaptive myelination in response to neuronal activity continuing into adulthood and contributing to learning, memory, and motor function^26^. The regional variation in oligodendroglial developmental dynamics likely reflects the different requirements for myelination in maintaining vital functions versus important roles in plasticity. Transplantation studies have demonstrated cell-extrinsic and -intrinsic regional effects on OPC maturation comparing adult neocortical gray matter and underlying white matter. The white matter was a more conducive environment for OPC differentiation and produced OPCs that differentiated more efficiently than gray matter OPCs, regardless of the region of transplantation^27^. The heterogeneous electrophysiological properties of OPCs also influence their functional states and show both age-dependent and regional variation^28^.

However, regional differences in oligodendroglial populations are understudied, especially pertaining to the brainstem and tumorigenesis, and mechanisms underlying the exquisite spatiotemporal specificity of DMG H3 K27M remain unclear^3,29^. Here, we use in vivo and in vitro approaches employing early postnatal wild-type and H3.3 K27M knock-in mice to identify distinguishing differentiation dynamics and transcriptional signatures between oligodendrocytes from wild-type telencephalon and brainstem in normal development, and to demonstrate region-specific effects of H3 K27M relevant to DMG pathogenesis.

## Results

### Expression of H3.3 K27M in embryonic NSPCs and their subsequent progeny causes neurodevelopmental defects

**Fig. 1:**
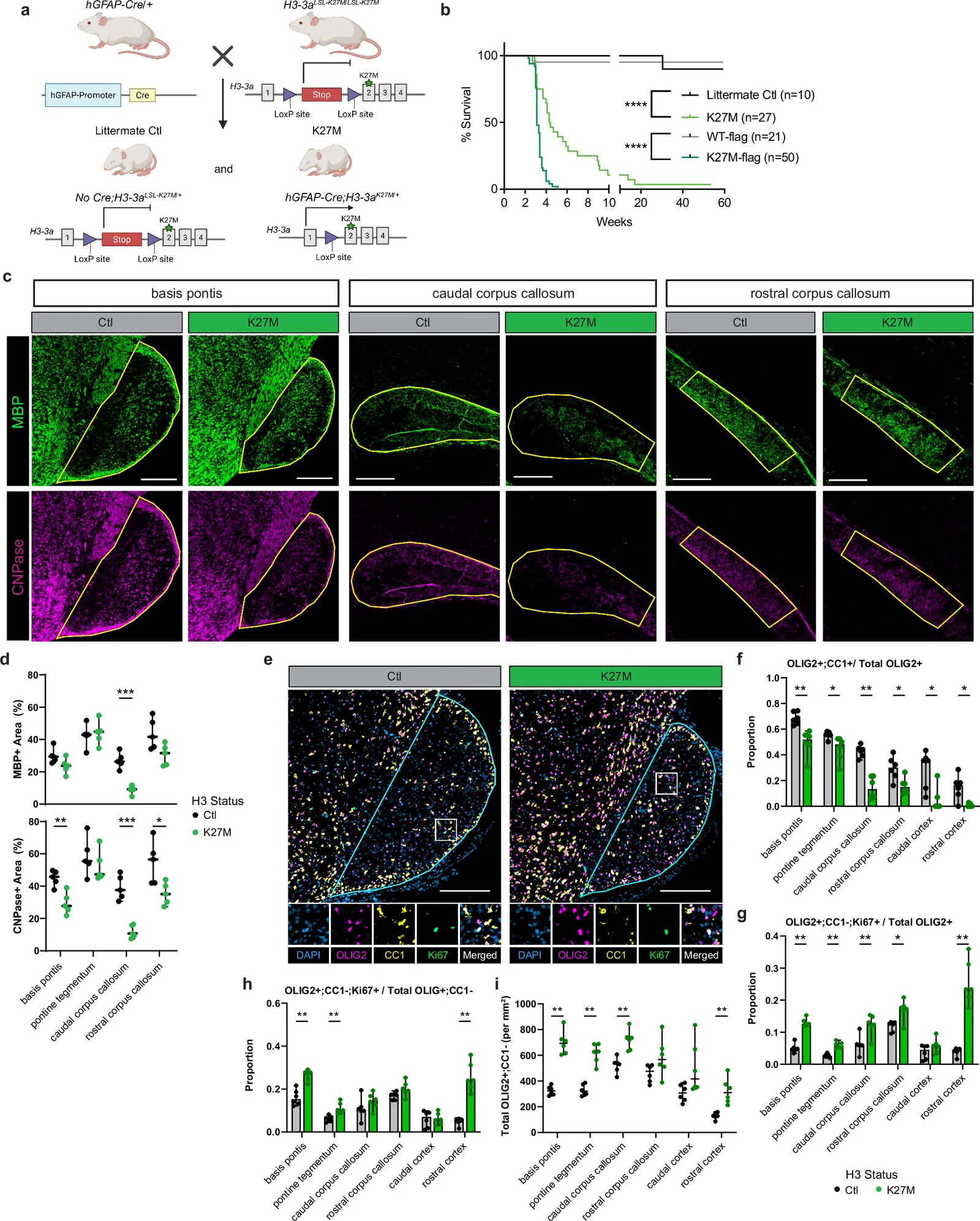
H3.3 K27M reduces mature oligodendrocytes (mOL) and alters oligodendrocyte (OL) lineage composition in a region-specific manner. **a** Diagram of *H3-3a*^*LSL-K27M*^ and *hGFAP-Cre* transgenic alleles and experimental breeding scheme (Ctl and K27M). Created in BioRender. Dobson, C. (2026) https://BioRender.com/ctnq88r. **b** Survival curves of WT-flag (*n* = 21), K27M-flag (*n* = 50), K27M (*n* = 27), and their littermate control mice (*n* = 10) (two-tailed Log-rank test, K27M-flag vs WT-flag *p* < 0.0001; K27M vs Ctl *p* < 0.0001). **c** Representative IF images for mOL markers MBP and CNPase at P14 in the basis pontis and caudal/rostral corpus callosum in Ctl and K27M mice. Scale bars are 200 μm. **d** Quantification of MBP+ (top) and CNPase+ (bottom) areas in specified regions at P14 in Ctl and K27M mice (*n* = 5; Student’s unpaired, two-tailed *t* test with Benjamini-Hochberg adjustment). **e** Representative images of immunofluorescence labeling of CC1 (mOL marker), OLIG2 (pan-OL lineage marker), Ki67 (proliferation marker), and DAPI in the ventral pons at P14 in Ctl and K27M mice. Scale bars are 200 μm. Cyan outlines highlight areas used for basis pontis quantification. **f**–**i** Quantification of proportion of mature OLs in total OLIG2+ lineage (**f**), proliferating oligodendrocyte progenitor cells (OPCs) in total OLIG2+ lineage (**g**), proliferating OPCs in all OPCs (**h**), and OPC density (**i**) in different brain regions in Ctl and K27M mice at P14 (*n* = 6; two-sided Mann-Whitney U tests with Benjamini-Hochberg adjustment; Supplementary Data 1). For **d**, **f**–**i**, each point represents the average for 2 sequential slices from a mouse with error bars indicating median ± 95% CI; *p*.adj < 0.05: *; *p*.adj < 0.01: **; *p*.adj < 0.001: ***. Source data are provided as a Source Data file.

To investigate how H3.3 K27M contributes to region-selective DMG in a developmentally and physiologically relevant context, we utilized *H3-3a-LSL-K27M* conditional knock-in mice expressing H3.3 K27M from the endogenous *H3-3a* locus following Cre-mediated deletion of the *loxP*-STOP-*loxP* cassette (LSL) (Fig. 1a). Because the cell of mutation in which K27M mutations first arise, subsequently leading to DMG, remains unclear, we chose an unbiased approach to probe the region-specificity and cell state-dependent effects of K27M during glial development. We bred *H3-3a-LSL-K27M* to *hGFAP-Cre* mice^30^, which constitutively express Cre in neural stem and progenitor cells (NSPCs) starting at ~E13.5 (Fig. 1a). Cre activity and conditional expression of H3.3 K27M or wild-type H3.3 with a C-terminal Flag tag (K27M-flag and WT-flag, respectively)^10^ was confirmed by nuclear Flag staining that recapitulated H&E staining of nuclear architecture in NSPCs and all lineages arising from them, including most glia and many neurons (Supplementary Fig. 1a, b).

As expected, H3K27me3 is detectable in WT-flag but not in K27M-flag mice (Supplementary Fig. 1c, d). K27M-flag mice died of unknown causes by 4 weeks of age, while K27M mice lacking the flag tag lived longer but still had reduced survival compared to controls (Fig. 1b). Brain MRIs at postnatal day 21 (P21) in K27M-flag mice revealed significant decreases in whole brain, corpus callosum, and hippocampal region volumes with increased lateral ventricle volumes compared to WT-flag and littermate control mice (Supplementary Fig. 1e–i). We confirmed hGFAP-Cre-mediated recombination occurred in >91% of Olig2-expressing oligodendrocyte (OL)-lineage cells in the pons and corpus callosum with no difference in Cre-activity between K27M-flag and WT-flag genotypes (Supplementary Fig. 2).

### H3.3 K27M reduces mature oligodendrocytes and increases proliferating OPCs in a region-specific manner

To study gliogenesis, we focused on the early postnatal 1–3 week period when massive expansion and differentiation of glial progenitors occur. To remove potential contributions from the epitope tag, we used conditional knock-in mice without the flag tag (K27M) and their littermate controls (Ctl) (Fig. 1a, b).

We assessed oligodendrocyte maturation in the developing mouse brain at P14 by immunofluorescence staining (IF) of CNPase, a marker of mature oligodendrocytes (mOL), and myelin basic protein (MBP), a major component of myelin. Because K27M mutation was associated with variable volumetric changes across brain regions, we evaluated regional phenotypes by comparing the proportions of immunopositive cells within anatomically defined regions (Fig. 1c). Within the ventral pons, which is proposed as a primary site of origin for DMG H3 K27M, quantification revealed reduced CNPase+ area with K27M in the basis pontis but not the tegmentum (Fig. 1d). CNPase+ areas were significantly reduced in K27M mice in rostral and caudal regions of corpus callosum, the main myelin tract in the telencephalon. MBP+ areas showed similar patterns to CNPase in these regions, although the MBP decrease in K27M mice only reaches statistical significance in the caudal corpus callosum (Fig. 1c, d).

Based on region-specific reduction in CNPase and MBP, we hypothesized that K27M may alter oligodendrocyte maturation. Thus, we co-immunostained for Olig2 (a pan-OL lineage marker), CC1 (a mOL marker), and Ki67 (a proliferation marker) at P14 (Fig. 1e; Supplementary Fig. 3 and Supplementary Data 1). The total proportion of oligodendrocyte lineage cells (Olig2+) was not significantly changed by K27M. However, we found a significant K27M-dependent reduction in the proportion of CC1+ mOLs and a corresponding increase in the proportion of proliferating OPCs (Olig2+; CC1−; Ki67+) as compared to all Olig2+ cells across both brainstem and telencephalon regions (Fig. 1f, g). The proportion of OPCs proliferating increased in the basis pontis, pontine tegmentum, and the rostral cortex (Fig. 1h), but the proportion of all proliferating cells that were OPCs only increased significantly in the basis pontis (Supplementary Fig. 3c). Notably, K27M increased total cell density in all regions assessed (Supplementary Fig. 3d) but only significantly increased Olig2+ cell density in the pontine tegmentum and rostral cortex (Supplementary Fig. 3e).

MBP and mature oligodendrocyte (mOL) markers, CNPase and CC1, are reduced across multiple brain regions, with the largest decreases observed in the caudal corpus callosum (Fig. 1d, f). In contrast, the basis pontis exhibits the greatest increase in the proportion of proliferating OPCs and the density of OPCs (Fig. 1h, i), highlighting regional variation in K27M-dependent effects on oligodendrocyte development.

### H3.3 K27M increases region-specific progenitor cell postnatal proliferation

**Fig. 2:**
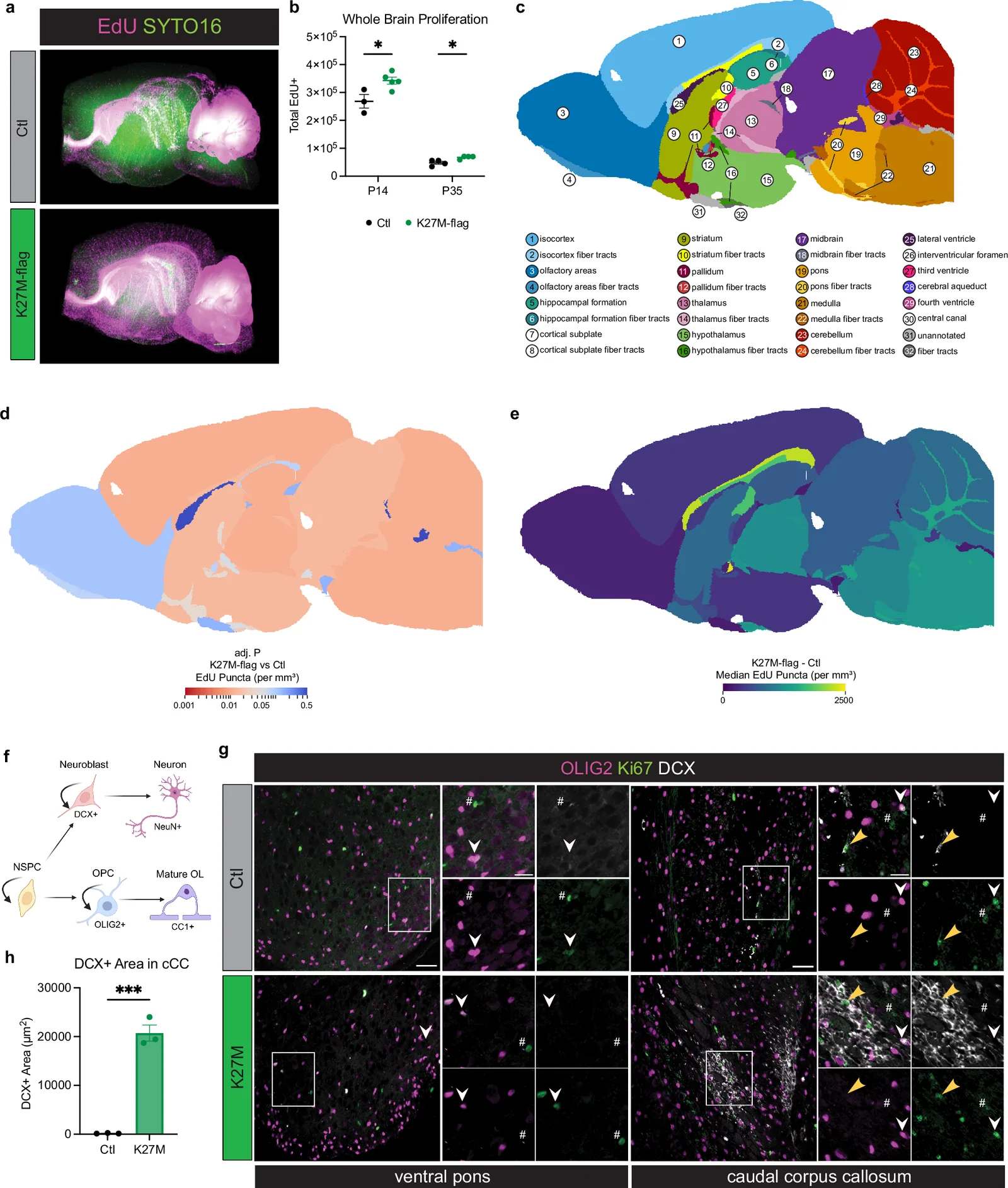
H3.3 K27M increases postnatal proliferation of distinct neural lineage progenitor populations by brain region. **a** A representative 3D rendering of a cleared brain with Click-iT detection of EdU, SYTO16 nuclear stain, and whole-brain imaging in P14 K27M-flag and Ctl mice. **b** Quantification of total EdU+ cells in whole cleared brains at P14 (Ctl *n* = 3; K27M-flag *n* = 5; adj. *P* = 0.031649) and P35 (*n* = 4 per group; adj. *P* = 0.031649) with two-tailed unpaired Student’s *t* test, Holm-Sidak adjustment. **c** Broad region atlas used for whole cleared brain quantification. **d** Per region adjusted *P* values comparing volume-normalized EdU puncta counts for P14 K27M-flag and Ctl (*n* = 6 per genotype; Two-sided Mann–Whitney U test with Benjamini-Hochberg adjustment; Supplementary Data 2). **e** Per region difference in median volume-normalized EdU puncta count between K27M-flag and Ctl mice**. f** Graphical depiction of neural cell lineages and markers. Created in BioRender. Dobson, C. (2026) https://BioRender.com/1ebiy3i. **g** Representative immunofluorescence images of OLIG2, Ki67, and DCX in the ventral pons and caudal corpus callosum in P14 K27M and Ctl mice. Scale bar is 50 μm, or 20 μm in the boxed high magnification insets. Marker combinations identified by #(Ki67+; OLIG2−; DCX−), white arrow (Ki67+; OLIG2+; DCX−), and yellow arrow (Ki67+; OLIG2−; DCX+). **h** Quantification of DCX+ area in the caudal corpus callosum (*n* = 3; unpaired Student’s *t* test; *p* = 0.00024023). For **b** and **h**, each point on the graph represents a measurement from one mouse with error bars showing mean ± SEM. *p* < 0.05: *; *p* < 0.001: ***. Source data are provided as a Source Data file.

To investigate how K27M confers region-specific predisposition to transformation in early postnatal progenitor cells, we performed in vivo EdU-labeling, whole brain tissue clearing, and imaging by light-sheet microscopy to visualize proliferating cells. We pulse-labeled K27M and littermate control mice with EdU at P14 or P35. In control mice, the number of proliferating cells declined from P14 to P35, consistent with the timing of early postnatal gliogenesis, with continued proliferation as expected in the subventricular zone (SVZ) and rostral migratory stream (RMS) at P35 (Fig. 2a, b). We found K27M increased proliferation throughout the brain at P14 and P35 (Supplementary Videos 1 and 2).

We next imaged cleared brains from a larger cohort of P14 mice with both EdU-labeling and Olig2 staining or CNPase staining alone. Cleared brain images were registered to a custom mouse brain atlas (derived from^31^) to quantify proliferating or Olig2+ cells by region (Fig. 2c and Supplementary Data 2). K27M significantly increased proliferating cells in most brain regions, with the greatest increases in fiber tracts, brainstem (medulla and pons), and thalamus (Fig. 2d, e). K27M significantly increased Olig2+ cells in the thalamus and striatum only, and showed the greatest increase in pons, although this was not significant (p.adj = 0.067) (Supplementary Fig. 4). We used CNPase architecture to generate matched images for quantification of basis pontis size, confirming reduced basis pontis size in K27M mice (Supplementary Fig. 5). Taken together, these data suggest K27M may sustain pools of proliferating progenitors and increase proliferation throughout the brain beyond the normal developmental window, which may contribute to altered brain morphology.

To determine if other neural cell lineages were hyperproliferative with K27M, we stained for Ki67, Olig2, and neuroblast marker Dcx at P14 (Fig. 2f–h). No Dcx+ neuroblasts were observed in the ventral pons in either K27M or Ctl mice. With K27M, there was a marked increase in Dcx+ neuroblasts in the caudal corpus callosum, which appear to emanate from outside of the normal proliferative zone of the SVZ/RMS (Fig. 2g, h). In addition, P14 K27M brainstem tissue exhibited reduced expression of *Rbfox3*, the gene encoding the mature neuron marker NeuN (Supplementary Fig. 6a), and K27M mice had reduced NeuN+ neurons in the ventral pons at P21 (Supplementary Fig. 6b, c).

Previous studies reported that PRC2 deficiency in OPCs causes OPC-to-astrocyte fate switch in both the cortex and brainstem^32,33^. Notably, we identified no K27M-dependent change in astrocyte development by immunostaining or qRT-PCR for astrocyte markers in the telencephalon or brainstem at P14 (Supplementary Fig. 7), indicating that K27M has different effects on OL-lineage fate than complete PRC2 deficiency.

Taken together, these studies show K27M expands progenitor pools throughout the brain with regional variation in lineage type. K27M exerts OL-lineage-selective effects in the ventral pons in vivo, with increased proliferation and delayed or restrained maturation that could provide a window of increased susceptibility to transformation.

### H3.3 K27M does not induce novel cellular states in the early postnatal developing mouse brainstem

We previously showed H3.3 K27M selectively accelerated gliomagenesis in the brainstem of an in vivo model recapitulating the regional selectivity of the mutation that is the foundation of DMG^10^. Cellular states present in the developing brainstem have not been well-established, so we utilized single-cell RNA-seq to resolve cell states across normal development and assess the effects of H3.3 K27M alone in early postnatal brainstem development. We performed single-cell RNA-seq on whole brainstem tissue in K27M and littermate controls. We used timepoints reflecting significant gliogenic phases in the pons: the highest density of proliferative cells (P4), the largest total number of progenitor cells (P14), and when gliogenesis is largely complete with mostly mature cells and few progenitors remaining (P21)^16^. After processing and quality control, 92,053 total cells remained, which were integrated, clustered, and annotated based on known markers (Supplementary Fig. 8a, b). K27M genotype and expression were confirmed in expected cell types and samples (Supplementary Fig. 8c, d). Interestingly, we found no cell clusters unique to K27M expression, indicating K27M expression in NSPCs does not induce a previously unidentified abnormal cell type. K27M did not significantly alter cell type proportions at any timepoint, suggesting subtle effects on cell state not easily resolved via the singular snapshot provided by scRNA-seq (Supplementary Fig. 8e).

To assess the cell type-specific effects of K27M, we performed differential expression analyses between K27M and control on pseudobulk profiles of each timepoint and cell type (Supplementary Data 3). Cells in the glial lineages (astrocytes and oligodendrocytes) were most impacted, retaining signatures of immature OPCs and reduced differentiated cell state signatures (Supplementary Data 4 and Supplementary Fig. 9a). K27M also drives intrinsic neuronal expression programs in both the astrocyte and oligodendrocyte lineages. When comparing K27M and control expression along pseudotime of the oligodendrocyte lineage via condition testing, we identified significantly reduced expression of genes associated with OL differentiation (*Gpr17*, *Qdpr*) and increased expression of neuronal genes (*Tenm3*, *Slit2*) in K27M (Supplementary Fig. 9b, c and Supplementary Data 5). We defined a robust, 156-gene, cell-type-agnostic signature of the K27M mutation enriched for developmentally relevant GO terms (Supplementary Fig. 9d and Supplementary Data 6). This signature is highly expressed in all cell types with K27M expression (Supplementary Fig. 9e, f).

Altogether, these results suggest that K27M does not alter the global identity of developing brainstem cell states but may induce expression of some genes normally associated with neural progenitors, enforce extended expression of pre-OPC and OPC signatures, and restrain expression of oligodendrocyte maturation and myelination-relevant transcriptional programs.

### H3.3 K27M drives cell-autonomous increases in brainstem OPC proliferation

**Fig. 3:**
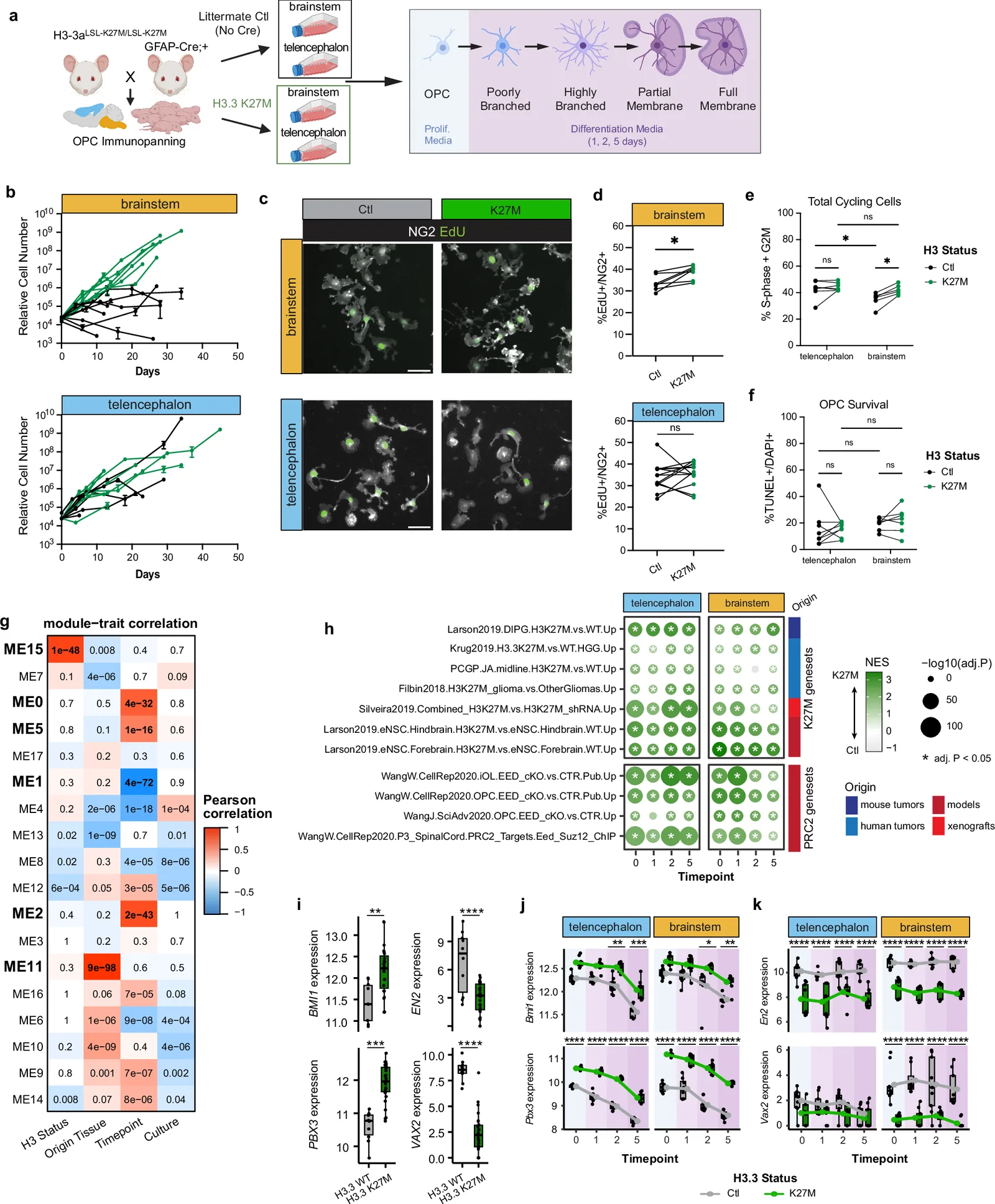
H3.3 K27M intrinsically increases brainstem OPC proliferation and recapitulates DMG H3 K27M tumor signatures. **a** Schematic for in vitro differentiation of OPCs from the brainstem and telencephalon of H3.3 K27M and littermate control mice. (BioRender: Dobson, C. (2026) https://BioRender.com/6ox3ppz). **b** Growth curves. Each line = average cell number from independent OPC cultures in proliferation media; points/error bars = technical triplicate mean ± SEM (*n* = 8 genotype pairs per region; linear mixed effect models, Location × Genotype × Days interaction; Wald’s t, two-sided, df via Satterthwaite, *p* = 0.0136). **c** Representative IF images of EdU and NG2. Scale bars, 50 μm. **d** Proportion of proliferating OPCs per genotype (two-tailed paired *t* test; brainstem *p* = 0.0159, *n* = 7; telencephalon *p* = 0.5006, *n* = 13). **e** Cycling cells from FACS (two-way ANOVA, Fisher’s LSD; brainstem *n* = 6; telencephalon *n* = 7; brainstem Ctl vs K27M *p* = 0.0204; Ctl telencephalon vs Ctl brainstem *p* = 0.0102). **f** Apoptosis (TUNEL + /DAPI+ nuclei) (brainstem *n* = 7; telencephalon *n* = 8; two-way ANOVA, Fisher’s LSD). **d–f** each point = average from independent culture, paired samples connected; ns = not significant, *p* < 0.05: *, *p* < 0.01: **. **g** WGCNA heatmap of module–trait correlations from RNA-seq of cultured/differentiated OPCs (*n* = 9 per location/genotype/timepoint except brainstem K27M 2d and brainstem Ctrl 1d where *n* = 8). *P* values per module/trait (two-tailed asymptotic *t* test on correlation coefficients). Bold = modules with correlation >0.5 or <−0.5, *p* < 0.001, and no correlation to culture of origin. **h** GSEA dotplot of NES for K27M or PRC2-related signatures, K27M vs control (*n* = 9 except brainstem K27M 2d and brainstem Ctrl 1d where *n* = 8). Significance from two-tailed permutation testing + Benjamini–Hochberg (Supplementary Data 9). **i** RNA-seq boxplots of log_2_(normalized counts + 1) for known H3.3 K27M up- (*BMI1* adj.p = 0.007531, *PBX3* adj.p = 0.000528) and down-regulated (*EN2* adj.p = 2.314E-09, *VAX2* adj.p = 1.785E-09) genes in primary midline high-grade gliomas (H3 WT *n* = 10; H3.3 K27M *n* = 27; two-tailed Wald test, Benjamini-Hochberg^43^,). **j**,** k** RNA-seq boxplots for H3.3 K27M-associated genes (as in (**i**)) in the OPC differentiation cohort by origin and timepoint (*n* = 9 except brainstem K27M 2 d and brainstem Ctrl 1 d, where *n* = 8; two-tailed Wald test, Benjamini-Hochberg, p.adj<0.05, |FC | > 1.1). The line connects the group average per timepoint. Boxplots: center = median, box = quartiles, whiskers = 1.5 × IQR. For **h–k**: p.adj < 0.05: *, <0.01: **, <0.001: ***, <0.0001: ****. Source data provided as a Source Data file.

Brainstem and telencephalon oligodendrogenesis occur asynchronously, with OPC differentiation and myelination beginning much earlier in the brainstem than the telencephalon^34–36^. NG2+ OPCs are broadly distributed at P0, and MBP+ mOLs are present at P0 in the brainstem but not until P4 in the telencephalon (Supplementary Fig. 10). Additionally, OPC proliferation and differentiation within a region do not proceed in a synchronized fashion. Thus, to study the regional dynamics and K27M effects on OPC proliferation and differentiation, we pooled brainstem and dorsal telencephalon tissue from K27M and littermate control mice at P5–P7 and immunopanned for CD140a+ OPCs to establish purified primary OPC cultures (Fig. 3a)^37^.

We first assessed the regional, cell-autonomous effects of K27M in OPC proliferation. Growth curves showed that K27M did not significantly alter the proliferation of telencephalon OPCs. In contrast, control brainstem OPCs expanded significantly slower than telencephalon OPCs, and K27M increased brainstem OPC proliferation to control and K27M telencephalon levels (linear mixed effect models, Location*Genotype*Days interaction term; Wald’s t-static, *p* = 0.0136; Fig. 3b). FACS cell cycle analysis and EdU-incorporation confirmed fewer cycling control OPCs in the brainstem than in the telencephalon, and K27M increased the proportion of cycling brainstem OPCs but not telencephalon OPCs (Fig. 3c–e). OPC survival did not differ by brain region or with K27M (Fig. 3f). These results indicate that early postnatal brainstem OPCs are intrinsically less proliferative than telencephalon OPCs and that K27M drives a cell-autonomous increase in brainstem OPC proliferation.

### Expression signatures of H3.3 K27M cultured OPCs throughout differentiation recapitulate DMG H3.3 K27M tumor signatures

We next utilized this region-specific primary OPC culture system to identify the transcriptional impact of K27M in synchronized differentiating oligodendrocyte lineage by culturing primary brainstem and telencephalon-derived OPCs in differentiation media for 1, 2, or 5 days (day 0 cultures remained in proliferation media) followed by RNA-seq (Fig. 3a). To identify expression programs associated with brain region of origin, H3 status, and differentiation in an unsupervised manner, weighted gene correlation network analysis (WGCNA) was performed on the gene count matrix, and the resulting gene modules were correlated with metadata traits (Fig. 3g and Supplementary Data 7). Multiple gene modules (MEs 0–2,5) were significantly and robustly correlated with differentiation timepoints, but only single modules correlated with H3 status (ME15) or origin tissue (ME11). ME11 contains Hox family genes and well-known transcription factors for telencephalon (*Foxg1*, *Emx2*, *Lhx2/9*, etc.) and brainstem (*Pax6*, *Irx1/2/3/5*, *Gbx1/2*, etc.) origin and identity (Supplementary Fig. 11)^10,38–41^. Notably, these developmental origin-specific gene signatures largely persisted through differentiation regardless of H3 status.

To connect the developmental impact of K27M in the OL-lineage with DMG H3 K27M pathogenesis, we determined the similarity of K27M-induced expression programs to those seen in DMG H3 K27M. We performed pre-ranked gene set enrichment analysis (GSEA) on differential expression analyses of the K27M and control OPC cultures at each timepoint split by origin tissue (Supplementary Data 8) using K27M up-regulated and PRC2 target gene sets taken or derived from published datasets of primary patient tumors, patient-derived orthotopic xenografts, mouse neural stem cell models, mouse OPCs, and mouse model tumors (Fig. 3h and Supplementary Data 9)^10,21,24,42^. These gene sets showed significant positive enrichment (p.adj < 0.05) in nearly all K27M versus control comparisons across timepoints and tissue origin. As expected, K27M up-regulated genes are positively enriched for PRC2 targets and genes up-regulated in PRC2-null OPCs and immature oligodendrocytes across timepoints and tissue origin.

Known DMG H3K27M up- (*BMI1, PBX3*) and down-regulated (*EN2*, *VAX2*) genes (p.adj < 0.05)^43^ in primary midline tumors (Fig. 3i) are recapitulated in the cultured OPCs (Fig. 3j, k). While some genes are recurrently affected by H3.3 K27M regardless of origin tissue, certain DMG H3 K27M markers reflect intrinsic location-specific expression patterns also observed in the cultured OPCs (e.g., *Vax2*). Together, these analyses demonstrate that K27M expression signatures in cultured proliferating and differentiating OPCs are representative of DMG H3 K27M signatures.

### Brainstem-derived OPCs exhibit delayed in vitro differentiation and are more primitive than telencephalon-derived OPCs

**Fig. 4:**
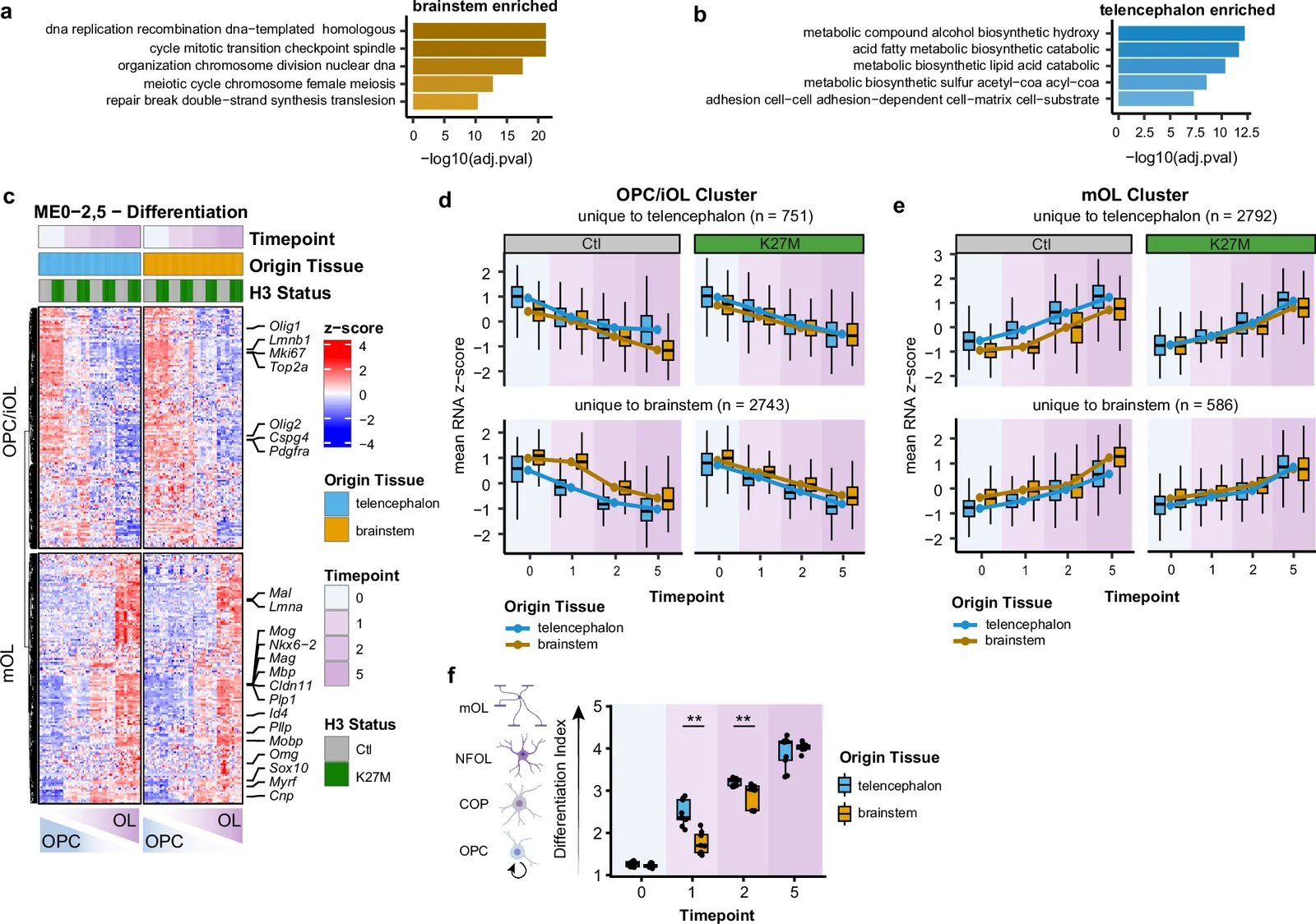
OPCs display different in vitro differentiation timing based on region of origin, which is ablated by H3.3 K27M. **a**, **b** Summary of top words describing gene ontology (GO) biological process terms identified by enrichment analyses of genes uniquely up-regulated (two-tailed Wald test, Benjamini-Hochberg adjustment, *P*.adj < 0.05, effect size testing threshold FC > 1.1) in control brainstem vs telencephalon (**a**) or telencephalon vs brainstem (**b**) oligodendrocyte progenitor cell (OPC) cultures at any timepoint. GO terms clustered by semantic similarity with a threshold of 0.7 and the top -log_10_(adjusted *P* value) (one-tailed hypergeometric enrichment test with Benjamini-Hochberg adjustment) for cluster members plotted. Word frequency was derived from all GO terms in each cluster with common stopwords removed. The top 5 clusters by max −log_10_(adjusted *P* value) of member terms are plotted. **c** Expression heatmap of genes in differentiation-associated WGCNA modules for all samples. Select OPC or oligodendrocyte lineage differentiation-associated genes labeled. Genes were clustered into OPC/immature OL (OPC/iOL) or mature OL (mOL) groups via k-means clustering (*k* = 2). **d**, **e** Boxplots of average **e**xpression z-scores for genes uniquely up-regulated (two-tailed Wald test, Benjamini-Hochberg adjustment, *P*.adj < 0.05, effect size testing threshold FC > 1.1, Supplementary Data 8) at any timepoint in either telencephalon or brainstem-derived control cultures for OPC/iOL (**d**) or mOL (**e**) cluster genes identified in (**c**). The line conn**e**cts the group average per timepoint. The same genes are shown for K27M cultures. **f** Boxplots displaying differentiation index derived from cell type proportions for control OPC cultures across differentiation timepoints (*n* = 9 per location/timepoint except brainstem Ctrl 1 day, where *n* = 8) as determined via MuSiC2 using the oligodendrocyte lineage from our developing mouse brainstem single-cell dataset as reference (Supplementary Figs. 8, 9 and Supplementary Data 11). Y-axis elements modified from a diagram created in BioRender. Dobson, C. (2026) https://BioRender.com/6ox3ppz. Two-sided Mann–Whitney U tests were performed between origin tissues at each timepoint, and adjusted *P* values generated via Benjamini-Hochberg adjustment (1 day *p* = 0.0013; 2 day *p* = 0.0055). *P*.adj < 0.05: *; *P*.adj < 0.01: **; *P*.adj < 0.001: ***; *P*.adj < 0.0001: ****. For boxplots, center line, median, box limits, upper and lower quartiles, whiskers and 1.5x interquartile range. Source data are provided as a Source Data file.

We examined baseline differences between normal brainstem and telencephalon by summarizing the biological processes of the genes uniquely up-regulated in either brainstem-derived OPC or telencephalon-derived OPC control cultures at any timepoint (Fig. 4a, b and Supplementary Data 10). Control brainstem-derived OPC cultures were most enriched for cell cycle and DNA replication terms (Fig. 4a). Alternatively, control telencephalon-derived OPC cultures were enriched for lipid and fatty acid metabolism, extracellular matrix, and adhesion terms (Fig. 4b), which are imperative to OL differentiation and myelination^44–47^. This suggests the OL-lineage in the telencephalon may be more primed for environmental cues to induce and sustain differentiation, while the brainstem OL-lineage may retain a more proliferative state through differentiation.

This was substantiated by investigating the spatiotemporal dynamics of OPC differentiation in these cultures. WGCNA identified multiple large gene modules associated with OPC/immature oligodendrocytes (iOL) or mOLs (Figs. 3g and 4c). We compared control cultures at each timepoint to identify genes that uniquely stratify samples by origin tissue at one or more timepoints. We then assayed overlap between these origin tissue-specific gene sets and the OPC/iOL (Fig. 4d) and mOL (Fig. 4e) clusters in the differentiation-associated WGCNA modules (ME0-2,5). Genes uniquely up-regulated in control brainstem-derived cultures have much greater overlap with the OPC/iOL cluster genes than those in the mOL cluster (2743 vs 586; Fig. 4d, e left), with the converse being true for genes uniquely up-regulated in control telencephalon cultures (751 vs 2792; Fig. 4d, e left). We also identified differentially expressed genes between sequential timepoints for each origin tissue, and the delayed induction of differentiation in brainstem-derived control OPC cultures is particularly apparent between the 1- and 2-day timepoints (Supplementary Fig. 12a–d left). In addition, control brainstem-derived OPC cultures maintain significantly higher expression of proliferative (*Mki67*, *Ccnb1*) and primitive OPC genes (*Lmnb1*, *Pdgfra*) throughout differentiation (Supplementary Fig. 12e). Conversely, early markers of committed oligodendrocyte precursors (*Gpr17*, *Fyn*) and oligodendrocyte maturation (*Plp1*, *Cnp*) are up-regulated more quickly in control telencephalon-derived OPC cultures (Supplementary Fig. 12f). These dynamics demonstrate a cell intrinsic delayed differentiation in brainstem-derived versus telencephalon-derived OPCs in vitro.

To compare our differentiated cultures to the fully resolved OL-lineage, we estimated the abundances of distinct OL-lineage cell types in our control cultures using deconvolution with our developing mouse single-cell dataset control samples as a reference and converted these proportions to a differentiation index for each sample (Supplementary Fig. 8; Fig. 4f and Supplementary Data 11). At 1 and 2 days of differentiation, telencephalon-derived OPC cultures had significantly higher differentiation indices driven by greater committed oligodendrocyte progenitor (COP) and newly formed oligodendrocytes (NFOLs) proportions, while brainstem-derived OPC cultures contained greater proportions of cycling OPCs. These differences were largely resolved by the 5-day timepoint.

These findings illustrate robust intrinsic differences in the cell state and differentiation dynamics of OPCs derived from brainstem and telencephalon. Telencephalon-derived OPCs display a more committed, or “primed,” state for differentiation while brainstem-derived OPCs exhibit an earlier OPC-state marked by delayed differentiation, sustained proliferation, and persistent early OL-lineage markers.

### H3.3 K27M mitigates origin tissue-specific differentiation dynamics, delaying OPC transition to COP in telencephalon and restraining full maturation of NFOL to mature oligodendrocytes in brainstem

We investigated whether K27M impacted the region-specific variations between telencephalon- and brainstem-derived OPC differentiation dynamics. Regional differences in OPC/iOL- (Fig. 4d) and mOL-associated (Fig. 4e) expression were lost with K27M (Fig. 4d, e right). K27M ablation of tissue-of-origin developmental patterns includes genes differentially expressed between sequential timepoints in control cultures from each origin tissue (Supplementary Fig. 12a–d right).

**Fig. 5:**
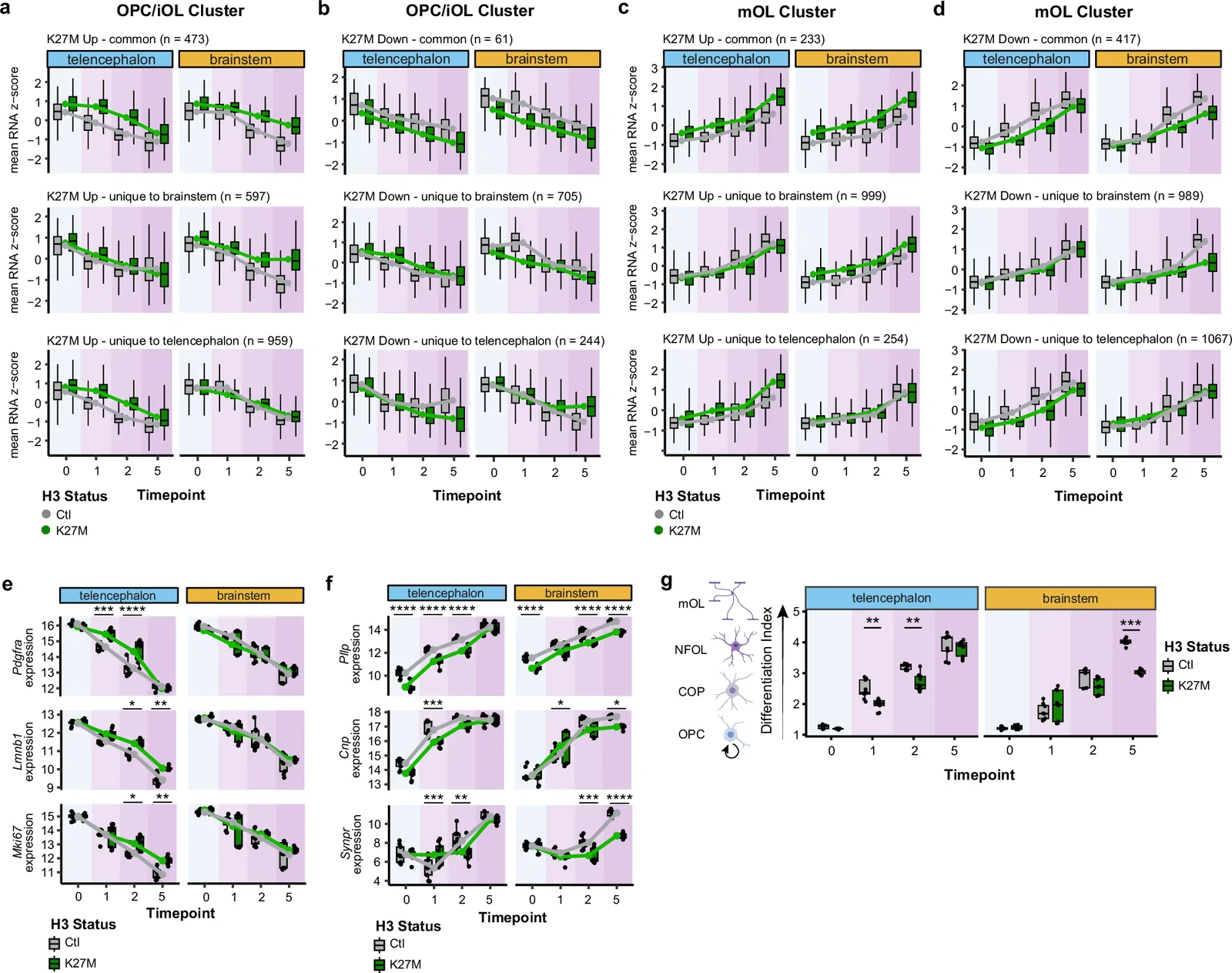
H3.3 K27M delays differentiation of telencephalon OPCs and restrains full differentiation in brainstem OPCs in vitro. **a**, **b** Boxplots showing the average expression z-score for genes in OPC/immature OL cluster of differentiation-correlated WGCNA modules (from Fig. 4c and Supplementary Data 7) up-regulated (**a**) or down-regulated (**b**) by K27M. **c** Same as in (**a**), but for the mOL cluster of differentiation-correlated WGCNA modules. **d** Same as in (**b**), but for the mOL cluster of differentiation-correlated WGCNA modules. Common (top), brainstem-specific (middle), or telencephalon-specific (bottom) gene sets. Telencephalon- (left) or brainstem-derived (right) OPC cultures. **e**,** f** RNA-seq boxplots for expression of representative OPC and proliferation genes (**e**) or OL differentiation genes (**f**) in telencephalon- and brainstem-derived K27M and Ctl OPC cultures across differentiation timepoints. The average for each group is connected via a line plot. Expression values plotted are log2(normalized counts + 1). Differentially expressed genes between genotypes at each timepoint indicated by asterisks. **g** Boxplots displaying differentiation index derived from cell type proportions for OPC cultures split by location across differentiation timepoints as determined via MuSiC2 using our developing mouse brainstem single-cell dataset as reference (Supplementary Figs. 8 and 9, same experiment as Fig. 4f and Supplementary Data 11). Two-sided Mann-Whitney U tests were performed between genotypes at each timepoint and adjusted *P* values generated via Benjamini–Hochberg adjustment within each panel (telencephalon 1d *p* = 0.0016, 2d *p* = 0.0016; brainstem 5d = 0.00016). For **e**–**g**, *P*.adj < 0.05: *; *P*.adj < 0.01: **; *P*.adj < 0.001: ***; *P*.adj < 0.0001: ****; *n* = 9 per location/genotype/timepoint except brainstem K27M 2 day and brainstem Ctrl 1 day where *n* = 8. For boxplots, center line, median, box limits, upper and lower quartiles, whiskers and 1.5x interquartile range. Differential expression determined via two-tailed Wald test with Benjamini-Hochberg adjustment, *P*.adj < 0.05, and effect size testing threshold |FC| > 1.1, see Supplementary Data 8. Source data are provided as a Source Data file.

After identifying K27M disrupted development, we examined differentiation dynamics by comparing common and unique differentially expressed genes between origin tissue and H3 status in the differentiation modules (Fig. 4c). Notably, K27M up-regulated OPC/iOL genes are most robust at 1- and 2-day timepoints in telencephalon-derived OPC cultures and the 5-day timepoint in the brainstem-derived OPC cultures (Fig. 5a). Conversely, OPC/iOL genes uniquely down-regulated in each K27M tissue arise from the early timepoints in brainstem-derived OPC cultures (Fig. 5b middle) and the 5-day timepoint in telencephalon-derived OPC cultures (Fig. 5b bottom).

mOL genes from K27M telencephalon-derived cultures were more impacted at the 1- and 2-day timepoints, suggesting an initial delay of differentiation that resolves (Fig. 5c, d top and bottom). This coincides with up-regulated proliferation and primitive OPC markers in K27M telencephalon (Fig. 5e), with delayed differentiation markers resolving by 5 days. In contrast, brainstem-derived OPCs with K27M failed to downregulate genes normally suppressed (Fig. 5d top and middle) or upregulate differentiated OL markers (Fig. 5f) later in differentiation.

These findings reveal that regional identity determines how K27M disrupts the transcriptional programs reflecting OPC-state and differentiation. In the telencephalon-derived OL-lineage, K27M dysregulates OPC/iOL and mOL programs mid-differentiation, which then resolve, indicating only a delay in differentiation. In the brainstem-derived OL-lineage, K27M dysregulates OPC/iOL and mOL signatures at later timepoints, consistent with preserving the OPC-state and preventing full OL maturation.

We determined the proportion of cell types at each timepoint by origin tissue and H3 status via deconvolution to derive a differentiation index (Fig. 5g and Supplementary Data 11). In line with previous observations, K27M telencephalon-derived OPC cultures had significantly lower indices at the 1- and 2-day timepoints that resolved by the 5-day timepoint, indicating a differentiation delay. Conversely, K27M brainstem-derived OPC cultures displayed significantly lower indices at the 5-day timepoint as compared to controls. We, therefore, propose that K27M dysregulates intrinsic region-specific transcriptional programs differently, resulting in distinct regional changes in OPC differentiation.

### Selective H3K27me3 loss at bivalent promoters drives H3.3 K27M-mediated transcriptional changes

**Fig. 6:**
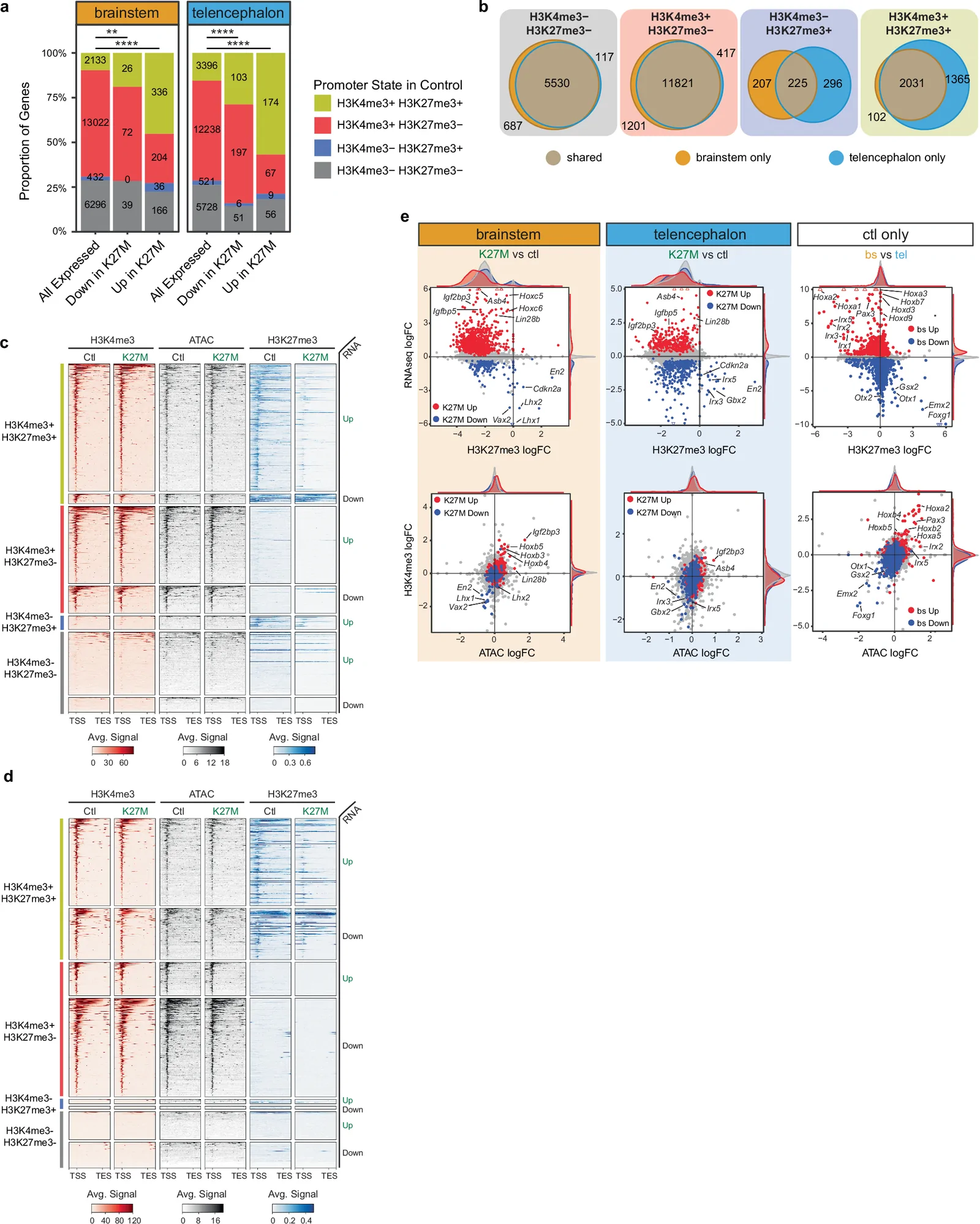
H3.3 K27M disproportionately affects bivalently marked genes. **a** A stacked bar plot showing the proportion of genes in each promoter state among all expressed genes, as well as genes up- or down-regulated in K27M relative to control, for brainstem and telencephalon. Numbers within bars indicate gene counts. Statistical significance of proportions was assessed using chi-square tests of independence in each tissue (two-tailed; brainstem down vs all *p* = 0.002859, up vs all *p* = 1.876E-215; telencephalon down vs all *p* = 7.297E-13, up vs all *p* = 1.778E-84; ns, not significant; *p* < 0.01: **; *p* < 0.0001: ****). **b** Venn diagrams showing overlap of promoter status of all genes in the brainstem and telencephalon. Shared, brainstem-specific, and telencephalon-specific genes are indicated. **c**,** d** Heatmaps of H3K4me3 ChIP-seq, ATAC-seq, and H3K27me3 ChIP-seq signals at gene bodies (TSS − 3 kb; TES + 3 kb) of K27M up- or down-regulated genes (two-tailed Wald test, Benjamini–Hochberg adjustment, p.adj < 0.05; effect size testing threshold |FC| > 1.1) for control and K27M conditions in brainstem (**c**) and telencephalon (**d**), grouped by promoter state and regulation direction. **e** Scatterplots of differential chromatin and transcriptional changes. Top: RNA-seq log_2_ fold change versus H3K27me3 log_2_ fold change in brainstem, telencephalon, and control-only comparison of brainstem versus telencephalon. Bottom: H3K4me3 log_2_ fold changes versus ATAC-seq log_2_ fold change. Select genes associated with K27M or tissue origin signatures are labeled. Densities of differentially expressed genes are shown as histograms on each axis. Source data are provided as a Source Data file.

We previously compared isogenic DMG models with and without K27M mutation and showed that K27M causes widespread loss of H3K27me3 at gene promoters, but a preferential transcriptional impact in mouse and human DMGs on genes with bivalent promoters marked by both H3K4me3 and H3K27me3 in wild-type controls^10,24^. To determine if K27M impacts gene expression through the same mechanism in OPCs in the developing brain, we performed ChIP-seq for H3K4me3 and H3K27me3 along with ATAC-seq in control and K27M brainstem- and telencephalon-derived OPC cultures to assess promoter state. We observed a significantly outsized proportion of bivalently marked genes when comparing promoter state proportions of K27M up- or down-regulated genes to all genes (Fig. 6a). Overlap analyses revealed that most genes with unmarked (H3K4me3-; H3K27me3-) and active (H3K4me3+; H3K27me3−) promoters were shared between control OPC cultures from each region while genes with repressed (H3K4me3−; H3K27me3+) or bivalent (H3K4me3+; H3K27me3+) promoters were shared less frequently between the regions (Fig. 6b).

The chromatin landscape at the transcription start sites of bivalent promoters demonstrates H3K27me3 loss in K27M up-regulated genes and H3K27me3 retention at K27M down-regulated genes in both brainstem- (Fig. 6c) and telencephalon-derived (Fig. 6d) OPC cultures. Both H3K4me3 and ATAC-seq were largely unaffected by H3 status. Importantly, many of the genes most strongly down-regulated by K27M in either tissue are key developmental and patterning transcription factors that retain or increase H3K27me3 at their promoters (e.g., *Irx* family members in telencephalon and *Lhx* members in brainstem; Fig. 6e). Together, this indicates that K27M drives transcriptional reprogramming in OPCs through depletion of H3K27me3 from bivalently marked promoters while maintaining H3K27me3-mediated repression at key developmental and tissue-identity genes.

### H3.3 K27M broadly sustains and up-regulates DMG-relevant developmental signaling pathways preferentially in brainstem-derived OL lineage

To further elucidate how K27M impacts OL lineage cell state regulation, we identified genes with uniquely or shared altered expression in K27M vs control cultures by origin tissue. We performed hypergeometric enrichment analyses on these gene sets, followed by semantic similarity analysis to summarize the impact of K27M on cultures from the two origin tissues. Shared K27M up-regulated gene sets include those for neuron/dendrite/axonal/synapse guidance and growth, early morphogenesis, cell adhesion, and WNT/MAPK signaling (Supplementary Fig. 13a). Many of these terms were highly enriched in early timepoints (1d, 2 d) during differentiation of the control cultures (Supplementary Data 10). Shared down-regulated pathways include many phospholipid, cholesterol, and catabolic synthesis pathways (Supplementary Fig. 13b), aligning with the reduced MBP seen in vivo with the H3.3 K27M mutation and likely accompanying decrease in myelination (Fig. 1).

Genes up-regulated by K27M uniquely in brainstem-derived OPC cultures were enriched for early morphogenesis, axonogenesis, gliogenesis, taxis/migration/adhesion/ECM, and synapse/neuronal projection terms (Supplementary Fig. 13c and Supplementary Data 10). In contrast, in telencephalon-derived OPC cultures, K27M uniquely up-regulated genes enriched for cell cycle, axon/synapse guidance, and neurogenesis (Supplementary Fig. 13d and Supplementary Data 10). While the timing and specific genes impacted by K27M differ between tissues, many similar biological processes are affected.

**Fig. 7:**
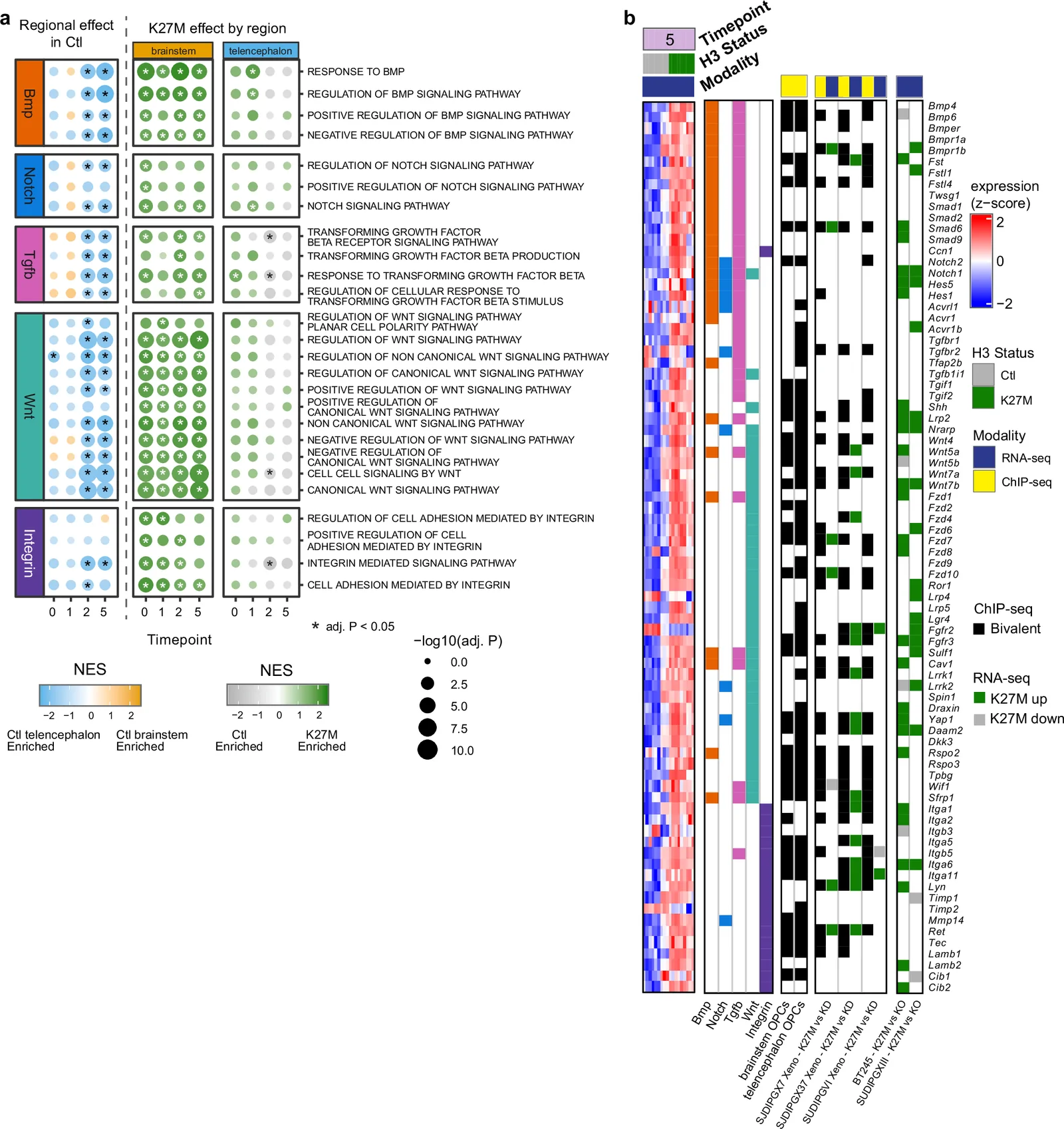
H3.3 K27M broadly dysregulates developmental signaling pathways preferentially in brainstem-derived OPC cultures. **a** Dotplot summary of pre-ranked GSEA results comparing control tissue region origin (left) and within-origin tissue comparisons of K27M and Ctl OPC cultures (right) for select developmental signaling GO biological process gene sets. Significant results (*P*.adj < 0.05) are marked with an asterisk. Adjusted *P* values (two-tailed) estimated from permutation testing followed by Benjamini-Hochberg adjustment. **b** 5-day expression heatmap of select genes in developmental signaling pathways that are up-regulated (two-tailed Wald test, Benjamini-Hochberg adjustment, *P*.adj < 0.05, effect size testing threshold FC > 1.1) by K27M in brainstem OPC cultures at one or more timepoints. Z-scores of normalized gene counts are shown. Row annotations include gene promoter bivalency (H3K4me3+/H3K27me3+) and differential expression in H3 K27M DMG xenografts with or without K27M shRNA from^24^, and H3 K27M DMG cell lines with or without K27M CRISPR-Cas9 from^42^. Source data are provided as a Source Data file.

However, developmental signaling pathways, such as Bmp, Notch, Wnt, Tgfb, and integrin, were broadly and strikingly up-regulated by K27M preferentially in brainstem-derived OPC cultures (Fig. 7a, b). At later stages of differentiation, expression of developmental signaling pathways is notably increased yet minimally affected by K27M in telencephalon-derived OPC cultures compared to brainstem-derived OPC cultures (Fig. 7a), again indicating that K27M ablates typical region-specific expression programs.

We next assessed bivalency status of these genes, finding 49% (39/80) and 68% (54/80) of the genes have bivalent promoters in control brainstem- or telencephalon-derived OPC cultures, respectively (Fig. 7b). To determine if these signaling pathway genes are impacted by K27M in DMG, we assessed their expression and promoter bivalency status in xenograft and cell line H3 K27M DMG models from previous studies where K27M was either knocked down via shRNA^24^ or knocked out via CRISPR-Cas9^23^, respectively (Fig. 7b). In total, we found that 73% (58/80) of the genes showed K27M-dependent up-regulation and/or promoter bivalency in at least one of the human DMG models (Fig. 7b and Supplementary Data 12).

K27M-mediated H3K27me3 loss activates bivalent genes with dual roles in neurodevelopment and tumorigenesis preferentially in the brainstem, providing a compelling epigenetic mechanism for region-specific vulnerability to DMG.

## Discussion

Diffuse midline gliomas (DMG), H3K27-altered (K27M), are defined by broad H3K27me3 depletion, an OPC-like transcriptional state, and a striking predilection for the pons and other midline structures despite K27M reducing H3K27me3 in many cellular contexts^18,20–22,48^. Here, we use an endogenous K27M knock-in model to perform regionalized in vivo and in vitro investigations and demonstrate that K27M does not affect the oligodendrocyte lineage throughout the brain uniformly. Instead, K27M exploits pre-existing regional differences in oligodendrocyte development to selectively expand a proliferative, differentiation-restrained OPC pool in the brainstem and aberrantly activates bivalently poised developmental signaling programs that are also engaged in DMG.

**Fig. 8:**
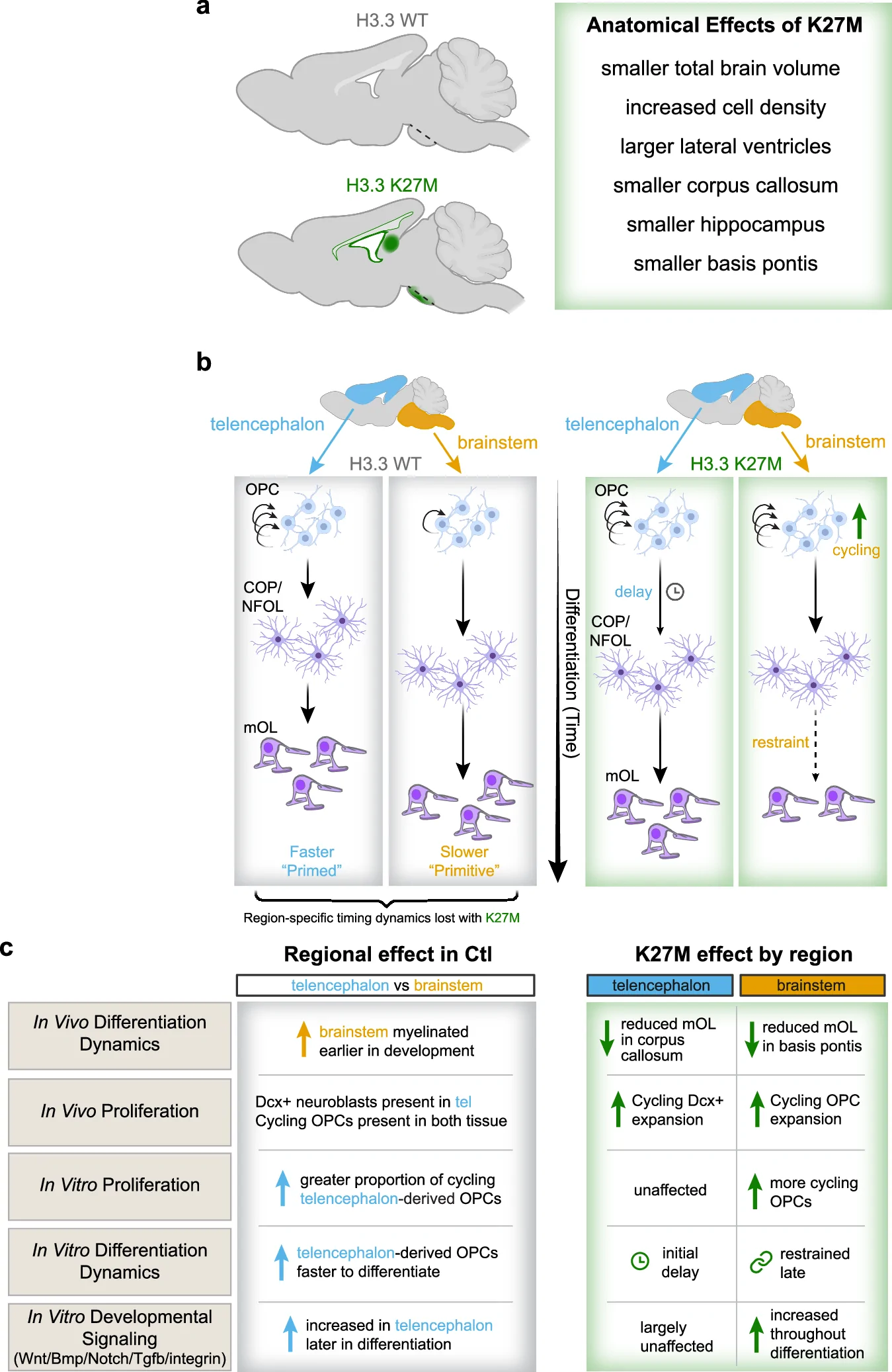
Schematic summary of the study. **a** An overview of K27M-driven anatomical changes. **b** Graphical summary of regional and K27M-driven differences in OPC differentiation of tissue-derived OPCs. Modified from a diagram created in BioRender. Dobson, C. (2026) https://BioRender.com/6ox3ppz. **c** Summary of in vivo and in vitro regional differences of the oligodendrocyte lineage and region-specific effects of K27M.

Whole-brain EdU labeling and light-sheet imaging demonstrate that K27M prolongs the window of progenitor proliferation beyond normal developmental timing with regional bias. K27M drives regional size differences, including dramatically decreased basis pontis size, increased lateral ventricle size, and increased cellular density throughout the brain (Fig. 8). These anatomical differences reflect shifts in lineage composition. K27M decreases mature oligodendrocytes and increases proliferating OPCs, with the largest proportional expansion of OPC density and proliferation in the ventral pons. Brainstem regions display robust expansion of OPCs and concurrent loss of NeuN+ neurons in the ventral pons, whereas the caudal corpus callosum saw a dramatic reduction in mature oligodendrocytes and an increase in Dcx+ neuroblasts not observed in the brainstem. Together, these in vivo data suggest that K27M reshapes postnatal gliogenesis in a region-restricted manner.

Recent single-nucleus RNA-seq analyses of adult human brain inferred lower OPC proliferation, slower oligodendrocyte differentiation, and reduced turnover of mature oligodendrocytes in the brainstem relative to telencephalon^49^. They hypothesized more frequent replenishment of mature oligodendrocytes in the telencephalon compared to the brainstem, aligning with our in vitro findings that wild-type brainstem OPCs proliferate and transition more slowly into committed oligodendrocyte progenitors than telencephalon OPCs, which appear “primed” for differentiation. In vivo, myelination and the rise of mature oligodendrocytes occur earlier in the brainstem than telencephalon^26,50^, and OPCs exhibit regional variation in differentiation capacity driven by local niche context (white versus gray matter) and age^27,28^. Our data suggest that both extrinsic cues and intrinsic OPC state, which K27M specifically perturbs, orchestrate these developmental timing differences.

K27M alters OPC proliferation and differentiation in a region-dependent manner, increasing brainstem OPC proliferation, slowing early differentiation of telencephalon OPCs, and restraining late-stage oligodendrocyte maturation in brainstem OPCs. We previously found that K27M increased in vitro proliferation of both forebrain and hindbrain neural stem cells (NSCs), indicating the acquisition of intrinsic properties during the NSC to OPC transition that result in regional differences in OPC response to K27M^10^.

Importantly, the K27M phenotype we observe is distinct from complete PRC2 loss induced by *Eed* deletion, which produces an OPC-to-astrocyte fate switch and broad lineage reprogramming in both cortex and brainstem^32,33^. Through single-cell sequencing as well as focused IF analysis of cell markers, we found no evidence of new cell types, cell fate shifts, or increased numbers of GFAP+ astrocytes, likely related to the relative extent of K27M-mediated disruption in PRC2-mediated methylation, which is dramatic but not equivalent to global PRC2 complete loss.

By integrating region-resolved in vivo imaging, single-cell and bulk transcriptomics, and epigenomic profiling with an in vitro OPC culture system, we mechanistically link regional developmental programs, bivalent chromatin, and the midline-restricted oncogenic potential of H3.3 K27M. At the chromatin level, K27M in DMG and neural stem cell models disproportionately affects genes whose promoters are bivalently marked by H3K4me3 and H3K27me3^10,14,24^. Consistent with these findings, here we show that K27M up-regulated genes in OPCs from both brainstem and telencephalon are enriched for bivalent promoters that lose H3K27me3 while retaining H3K4me3 and open chromatin at their transcription start sites. K27M simultaneously derepresses a subset of developmental programs while preserving repression of key patterning and identity transcription factors. The distribution of bivalently promoters is regionally patterned, suggesting that oncohistone-mediated re-programming will intersect distinct developmental networks in each region.

A central finding of our study is that K27M preferentially and robustly up-regulates multiple developmental signaling pathways in brainstem-derived OPCs. Bmp, Wnt, Notch, TGFβ, and integrin pathways play complex and varied roles in all stages of neurogenesis from stem cells through differentiation^51^. They are upregulated by K27M in brainstem-derived OPCs but intrinsically expressed throughout differentiation and not up-regulated by K27M in telencephalon-derived OPCs, reflecting context-dependent regional differences in developmental regulation. These regulators of signal transduction are collectively over-represented among K27M up-regulated, bivalently marked genes, and a majority of them show K27M-dependent up-regulation and/or promoter bivalency in human DMG models where K27M is knocked out or knocked down^24,42^. Bmp, Wnt, and Notch regulate glial lineage commitment, OPC proliferation, and myelination, and are linked to oncogenic behavior and invasion in DMG and other gliomas^18,22,52,53^. Genes within these pathways are also associated with super enhancers in patient-derived DMG cell lines^20^, highlighting the disease relevance of the K27M-induced signatures in the brainstem oligodendrocytic lineage.

Our data support a model in which K27M lifts PRC2 repression at a brainstem-biased set of bivalently expressed developmental genes, igniting aberrant, self-reinforcing signaling that sustains an immature, proliferative OPC-like state and may affect motility, adhesion, and axonal or synaptic interactions. Additional studies are necessary to better understand the regionally selective impacts of K27M on these pathways, how they relate to normal oligodendroglial development, and how they can be effectively targeted for therapeutic benefit in DMG.

DMG cells form neuron-glioma synapses, integrate into neural circuits, and exploit activity-dependent signaling to drive proliferation and invasion^54–56^, and genes associated with synapses and axon pathfinding are also associated with super-enhancers in DMG cells^20^. The observed cell-intrinsic K27M-dependent up-regulation of axon guidance, synapse, and neurite outgrowth programs, especially in brainstem-derived OPCs, provides a plausible developmental precursor to these malignant neuronal interactions. These K27M-driven changes align with prior observations of H3K27me3 deposition and PRC2 binding at neuronal genes during OPC differentiation^57,58^. Even before the acquisition of additional driver mutations, K27M OPCs in the brainstem may be biased towards heightened responsiveness to neuronal activity, trophic cues, and axonal scaffolding. The interplay between intrinsic regional OPC characteristics, K27M, and external cues contributing to pathogenesis warrants further investigation.

## Methods

### Animal studies

All experiments involving animals followed protocols approved by the Institutional Animal Care and Use Committee at St. Jude Children’s Research Hospital (Protocol # 278-100611) and were in compliance with institutional guidelines. Mice were maintained in a facility with a 12 h on/12 h off light cycle, temperature between 68–74 °F and humidity between 30–70%, automated watering with monitoring to ensure constant availability, a monitored ventilation system, daily welfare monitoring, and in barrier conditions. The day of birth is designated postnatal day 0 (P0). Male and female mice were utilized for all experiments and analyses. K27M-flag and WT-flag mice were described previously^10^. K27M mice without a tag were generated utilizing the same methods and constructs as the K27M-flag mice, except lacking the flag tag^10^. These mice were backcrossed onto the FVB background for at least 4 generations. hGFAP-Cre mice were obtained from the Jackson Laboratory (Strain #004600) and maintained on the FVB/N background. Mice were euthanized with Avertin and perfused with PBS.

#### Isolation and culture of mouse oligodendrocyte lineage cells

Primary OPCs were purified from P5-7 mice as previously described^37^ with minor modifications. Briefly, after euthanasia via Avertin and perfusion with PBS, brains were dissected from litters at P5-7 and the dorsal telencephalons (cortex, hippocampus, and corpus callosum) and brainstems were separated and pooled by region and genotype. Brain tissues were digested in papain buffer containing 1x EBSS supplemented with 1 mM MgSO_4_, 0.46% glucose, 2 mM EGTA, 26 mM NaHCO_3_, 20 units/ml papain, and 250 units/ml DNAse I under 5% CO_2_/95% O_2_ bubbling at 34 °C for 90 min. To make a single-cell suspension, tissues underwent multiple rounds of trituration with a P1000 pipette. The cells were resuspended in panning solution containing DPBS supplemented with 0.02% BSA and 5 µg/ml insulin and subsequently incubated on a BSL1-coated petri dish for 30 min to deplete endothelial cells and microglia. The remaining suspension was then incubated on a rat anti-PDGFRa-coated petri dish for 1 h to isolate OPCs. OPCs were trypsinized, spun at 220 × *g* for 15 min, and cultured on poly-D-lysine-coated plates in “proliferation medium” containing DMEM-SATO Base Growth Medium^37^ supplemented with 4.2 µg/ml forskolin, 10 ng/ml CNTF, 1 ng/ml neurotrophin-3, and 20 ng/ml PDGF in a 37 °C, 10% CO_2_ humidified incubator. OPCs were lifted with Accutase for passaging and subsequent assays. To differentiate OPCs, “proliferation medium” was replaced with “differentiation medium” containing DMEM-SATO Base Growth Medium supplemented with 4.2 µg /ml forskolin, 10 ng/ml CNTF, and 40 ng/ml thyroid hormone. Half of the culture medium was replaced every 2 days. Assays with control and H3.3 K27M region-specific primary OPC matched culture pairs were completed at the same time.

#### Histology, immunohistochemistry, and immunofluorescence staining

Mice were anaesthetized with Avertin and perfused with PBS. Brains were dissected out and fixed in 4% paraformaldehyde (PFA) for 48 h at 4 °C. NG2-GFP-expressing brains were fixed overnight, cryoprotected/embedded in OCT, and cut into 50 μm thick floating sections. Other brains were paraffin-embedded, and 5 μm thick sagittal sections were obtained. Hematoxylin and Eosin (H&E) staining (Thermo 7221, 7111) was performed according to the manufacturer’s instructions. Immunofluorescence staining and immunohistochemistry were performed utilizing heat-mediated antigen retrieval in a citric acid-based buffer (10 mM, pH 6.0) followed by blocking for 1 h at room temperature with 10% goat serum (Vector Labs) or 10% donkey serum (Jackson Immunoresearch) in PBS with 0.01% Tween-20 (PBST). The sections were incubated with diluted primary antibody in PBST with 2% goat serum or 2% donkey serum (antibody diluent) overnight at 4 °C, then washed 3 times in PBST. For immunofluorescence staining, the appropriate AlexaFluor Plus 488-, 555-, or 647-conjugated antibodies (Invitrogen; 1:500) and DAPI (Thermo; 62248; 1:500) in antibody diluent were added for 1 h at room temperature, washed 3 times in PBST, and mounted with ProLong Diamond Antifade Mountant (Invitrogen; P36961).

For immunohistochemistry, anti-rabbit biotinylated secondary antibody (Vector Labs; 1:1000) with horseradish peroxidase-conjugated streptavidin (VECTASTAIN Elite ABC Kit, Vector Labs, PK-6100). Staining was developed with DAB substrates (Vector Labs; SK-4100) and counterstained with hematoxylin (Vector Labs; H-3401). Sections were washed 3 times in PBST at room temperature and mounted with ProLong Diamond Antifade Mountant (Invitrogen; P36961). Images were taken on a Zeiss AxioVision, Keyence BZ-X800, Zeiss Axioscan, or Nikon C2 Confocal.

For Supplementary Fig. 10, samples were cryoprotected and embedded in OCT prior to sectioning of 50 µm sagittal free-floating sections. Free-floating IHC was performed with an InsituPro stainer (CEM) with images acquired with a Yokogawa CQ3000 confocal spinning disk microscope with a 20× objective. High-magnification images were obtained with a Leica Stellaris confocal microscope with a 63× objective.

Primary antibodies and dilutions:

**Table:** 

Name	Reactivity	Dilution	Company	Catalog #	Clone
OLIG2	Gt pAb	1:500	R&D	AF2418	
OLIG2	Ms mAb	1:1000	Millipore	MABN50	211F1.1
OLIG2	Rb pAb	1:200	Millipore	AB9610	
CC1 (APC)	Ms mAb	1:100	Calbiochem	OP80	Ab-7
MBP	Ms mAb	1:1000	Millipore	NE1019	SMI-99
MBP	Chicken pAb	1:500	Aves Labs	MBP	
GFP	Rb pAb	1:500	Abcam	Ab290	
CD31	Rat mAb	1:1000	BD Biosciences	553370	MEC13.3
CNPase	Rn pAb	1:1000	Encor	RPCA-CNP	
DCX	Ms mAb	1:100	Santa Cruz	SC271390	E-6
Ki67	Rb mAb	1:200	Cell Signaling	12202	D3B5
Ki67	Rb mAb	1:200	Abcam	Ab16667	SP6
H3K27me3	Rb mAb	1:500	Cell Signaling	9733	C36B11, lot 8
FLAG	Rb mAb	1:500	Cell Signaling	14793	D6W5B
FLAG	Ms mAb	1:500	Sigma	F1804	M2
NeuN	Ms mAb	1:200	Millipore	MAB377	A60
GFAP	Ms mAb	1:500	Cell Signaling	3670	GA5

#### Immunocytochemistry

5000–10000 OPCs were plated on poly-D-lysine-coated 12 mm coverslips in a 24-well plate in proliferation or differentiation media and incubated for 1–2 days for the OPC-state, or the specified number of days for the differentiation timepoint. Cells were fixed with 4% PFA for 10 min and permeabilized with 0.2% Triton-X in PBS for 20 min. Cells were blocked 1 h in 10% goat serum (Vector Labs) in PBS then incubated with primary antibody in 2% goat serum in PBS (antibody diluent) at 4 °C overnight. Coverslips were washed 3 times with PBS at room temperature and incubated with appropriate AlexaFluor Plus 488-, 555-, or 647-conjugated antibodies (Invitrogen; 1:500) and DAPI (Thermo; 62248; 1:500) in antibody diluent for 1 h at room temperature. Cells were washed 3 times in PBST at room temperature and mounted with ProLong Diamond Antifade Mountant (Invitrogen; P36961). Images were taken on a Zeiss AxioVision or Keyence BZ-X800. Primary antibodies and dilutions listed below:

**Table:** 

Name	Reactivity	Dilution	Company	Catalog #	Clone
CD140a	Rt mAb	1:200	BD Pharmigen	558774	APA5
NG2	Rb pAb	1:250	Millipore	AB5320	
CNPase	Ms mAb	1:1000	Millipore	MAB1326R	11-5B

### In vitro OPC growth curve

Early passage OPCs were plated at a density of 25,000 cells/T25 flask in proliferation medium in triplicate. Cells were serially counted and passaged in triplicate every 3–15 days, depending on when 80–90% confluency was reached in one or both flask groups. For statistical analysis, linear mixed effect models were fit, including intercept, Location, Genotype, Days in Culture, and their interaction terms. Sample IDs were treated as random effects. *P* values for specific model parameters are two-sided and based on Wald’s t-statistic with degrees of freedom estimated through the Satterthwaite approximation.

### In vitro OPC cell cycle analysis

Early passage OPCs were plated at a density of 100,000 cells/T25 flask in 3 mL proliferation medium and allowed to attach overnight. To label S-phase cells, 3 mL of proliferation medium containing 20 μM EdU was added to the OPCs (final concentration of 10 μM EdU) and incubated for 1 h. Cells were detached with Accutase and then fixed and permeabilized as noted above (Immunocytochemistry), and ClickIT reactions were performed with the Click-iT Plus EdU Alexa Fluor 488 Kit for Flow Cytometry per the kit protocol (Invitrogen). Cells were resuspended in 100 μl of FxCycle PI RNase Staining Solution (Invitrogen) and incubated on ice for at least 30 min prior to flow cytometry assessment on a Becton Dickinson Aria Fusion. Data was analyzed with BD FACSDiva v8.0.1. Gating was set on unstained and single stain samples, and >6500 cells were assessed per sample.

### In vitro OPC proliferation EdU Immunocytochemistry

5000 OPCs were plated on PDL-coated 12 mm coverslips in 500 μl of proliferation medium and allowed to attach overnight. To label proliferating cells, 500 μl of proliferation medium with 20 μM EdU was added to each well (final concentration of 10 μM of EdU) and incubated for 30 min. Cells were fixed and permeabilized as described above (Immunocytochemistry), and EdU was labeled with the Click-iT Plus EdU Alexa Fluor 647 Imaging kit per kit instructions (Invitrogen). Primary antibody staining was subsequently performed with CD140a (BD Pharmigen; 558774; 1:200) or NG2 (Millipore; AB5630; 1:250) antibodies, followed by secondary antibody and DAPI staining and mounting as described above. Images were taken on a Zeiss AxioVision or Keyence BZ-X800.

### In vitro OPC TUNEL staining

5000 OPCs were plated on PDL-coated 12 mm coverslips in 500μl of proliferation medium and allowed to attach overnight. Cells were fixed and permeabilized as noted above (Immunocytochemistry). To assess apoptotic cells, TUNEL reactions were performed with Click-iT Plus Alexa Fluor 647 Kits (Invitrogen) per kit instructions, followed by DAPI staining and mounting as noted above. Images were taken on a Zeiss AxioVision.

### Tissue section image quantification

Tissue section images were analyzed in Fiji (v1.54p)^59^ or QuPath (v0.6.0)^60^. The regions of interest (ROIs) to be quantified were manually segmented first for all images. For MBP and CNPase area quantifications in each ROI (Fig. 1c, d), a pixel thresholder was applied to label each pixel as positive or negative with manual verification of the resulting mask in QuPath. The percentage of positive pixels per ROI was then calculated.

For cell classification and quantification (Fig. 1e–g; Supplementary Fig. 3 and Supplementary Data 1), QuPath’s cell detection tool was first used on the DAPI channel using the full image resolution with a minimum area of 10 µm^2^, a maximum area of 200 µm^2^, and a cell expansion value of 3 µm to identify all nuclei and cell areas in each ROI. A composite training image was then generated using small regions of the brainstem and cortex from each image (24 total). Cells were manually annotated based on CC1, Olig2, and Ki67 staining as Olig2+/CC1−/Ki67−, Olig2+/CC1+/Ki67−, Olig2+/CC1−/KI67+, Olig2−/CC1−/Ki67+, or Olig2−/CC1−/Ki67− (at least 50 cells of each class). These cells were then used to train a random trees (RTrees) classifier in QuPath with features set to include only nuclear Ki67 signal, nuclear Olig2 signal, and both nuclear and cytoplasmic CC1 signal. The classifier was then applied to all cells in each ROI in every image. For Fig. 1c–g and Supplementary Fig. 3, two sections were imaged per mouse, and quantifications were averaged to a single value for each region.

For Cre activity quantifications (Supplementary Fig. 1c, d and Supplementary Fig. 2), Flag+ nuclei were semiautomatically segmented with Otsu’s threshold function on the greyscale Flag channel in Fiji with manual oversight. The Flag+ nuclei masks were placed onto the H3K27me3 or Olig2 channel, and the average intensity was measured within each nucleus. Positive versus negative nuclei were determined based on a fixed threshold.

For the Dcx+ area quantification (Fig. 2g, h), the Dcx+ area was manually segmented by adjusting the intensity threshold in Fiji until the area of Dcx positivity was covered, and the area was measured.

For %NeuN+ cells (Supplementary Fig. 6b, c), DAPI+ nuclei were semiautomatically segmented with Otsu’s threshold function within each ROI on the greyscale DAPI channel with manual oversight in Fiji. The DAPI+ nuclei masks were placed onto the NeuN channel, and the average intensity was measured within each nucleus. Positive versus negative nuclei were determined based on a fixed intensity threshold.

For average GFAP fluorescence intensity measurements (Supplementary Fig. 7a, b), the grayscale fluorescence intensity value per pixel within the ROI was measured and averaged over the ROI area.

In vitro OPC images were analyzed in Fiji^59^. For EdU quantifications (Fig. 3c, d), DAPI+ nuclei were semiautomatically segmented with Otsu’s threshold on the greyscale DAPI channel with manual oversight. The DAPI+ nuclei masks were placed onto the EdU channel, and the average intensity was measured within each nucleus. Positive versus negative nuclei were determined based on a fixed intensity threshold, and intact/live cells at the time of fixation were identified manually by CD140a+ or NG2+ staining. Apoptosis measurements (Fig. 3f) were obtained similarly, except on the TUNEL channel and without identification of intact/live cells.

### Whole-brain tissue clearing

#### Sample processing and image acquisition

Mice were injected with EdU (Sigma) at 50 mg/kg twice, with an interval of 2 h. Two hours after the second injection, mice were anesthetized with avertin (10 mg/25 g) and transcardially perfused with ice-cold PBS. The brain was carefully dissected and fixed with 4% PFA in PBS overnight at 4 °C. After PBS wash and cleaning up tissue surface under a dissecting microscope, the brain was dehydrated, delipidated, and bleached by the iDISCO+ protocol (http://www.idisco.info). The brain was further cleared by Shield and delipidation reagents from LifeCanvas Technologies (Cambridge, MA)^61^. Then, the brains were permeabilized by the iDISCO+ protocol, except using 0.5% Triton X-100 (Sigma) and incubating with 4 μM of SYTO16 (Thermo) for 5 days. The brain was washed with PBS three times and fixed with 4% PFA. After washing out with PBS, the brain was stained with Click-iT PLUS EdU Alexa 647 (Thermo) according to the manufacturer’s protocol, except using a half amount of copper supplement and incubating at 4 °C for 5 days. The brain was washed with PBS three times and dehydrated and index-matched by the iDISCO+ protocol.

For antibody staining, mice were perfused with ice-cold PBS and 4% PFA. The brains were cleared and stained with Olig2 antibody (R & D AF2418) or CNPase antibody (Encor RPCA-CNP) in the Smart Batch machine (Life Canvas, Cambridge, MA) according to the manufacturer’s instructions. Brains stained with Olig2 antibody were further stained with Click-iT PLUS EdU Alexa 555 (Thermo), index-matched with Easy Index (Life Canvas), and imaged by mesoSPIM microscope as described below. Brains stained with CNPase antibody were imaged using an Ultramicroscope II as described below.

Cleared brains were imaged using an Ultramicroscope II Gaussian light sheet system (Miltenyi Biotec) or mesoSPIM microscope^62^. This Ultramicroscope II is equipped with four laser lines at 405, 488, 532, and 639 nm with 100, 85, 100, and 70 mW input power, respectively and an MVX-10 objective mounted to a variable zoom body (Olympus) with magnification set to 0.63×. Chromatic focus correction in each channel is provided by an optomechanical lens unit prior to detection on a pco.edge 4.2 M camera (PCO). The excitation optics were set to the minimal NA of 0.008 to ensure that the Rayleigh length of the light sheet was commensurate with the width of the samples. For Ultramicroscope II imaging (Fig. 2a, b; Supplementary Fig. 5; Supplementary Videos 1, 2 and Supplementary Data 2), cleared samples were adhered to custom-printed pedestals using super glue and inserted into the microscope sample holder. The holder was submerged in DBE (Sigma Aldrich) in the imaging cuvette, and samples were scanned by moving the entire brain through the light sheet along the optical axis of the detection objective.

For mesoSPIM microscopy (Fig. 2c–e; Supplementary Fig. 4 and Supplementary Data 2), the samples were submersed in immersion oil (Cargille Laboratories, type A, RI = 1.52) within a primary glass cuvette (10 × 20 × 45 mm Hellma), mounted to a gantry holder and submersed in immersion oil again in a secondary cuvette (50 × 50 × 50 mm Hellma). The sample is illuminated from opposing sides with identical Gaussian light sheets shaped by a modified camera lens (Nikon AF-S 50 mm f/1.4 G) to create an approximately 5 μm thick light sheet that is axially-scanned (Optotune EL-16-40-TC-VIS-5D-1-C lens) and synchronized with a rolling shutter (Photometrics, Iris 15 sCMOS sensor) to form uniform light sheet thickness over the full field of view. Excitation was accomplished with a 561 nm and 640 nm laser source (Oxxius, L4Cc), and emission was filtered with a 50 nm bandpass at 600 nm and 700 longpass (Chroma), respectively. Imaging was performed using a Plan Apo 2X/0.055NA objective (Mitutoyo).

#### 3D brain registration and cell quantification

We made an end-to-end quantification pipeline that employed ilastik^63^, a machine learning-based segmentation software package, to detect EdU-positive or Olig2-positive nuclei throughout the entire brain. The images were then registered to a brain atlas optimized for light sheet fluorescence microscopy^31^ to quantify EdU or Olig2-positive cell counts per atlas region. First, the CLAHE plugin in Fiji^59^ was used to normalize the EdU channel. Using the normalized images, we trained an ilastik segmentation classifier on a representative set of full-resolution images to detect EdU-positive cells. This model was then applied to all other images. We also trained a separate ilastik segmentation classifier on either the Syto16 (Ultramicroscope II) or EdU (mesoSPIM) channel to distinguish the brain from the background. This model was trained on images downscaled by a factor of 0.2. The resulting segmentation mask was used to register the segmented template image of the atlas to each image using the affine method implemented in the ANTsX library^64^. We then up-scaled the brain mask and applied it to the segmented images to exclude regions outside the brain. The registration transform was used to map atlas regions to individual datasets. Finally, we counted EdU or Olig2-positive cells per atlas region and calculated the total count across all regions combined.

For more informative and interpretable comparisons, we collapsed the original high-resolution atlas into broad anatomical regions (Supplementary Data 2). Cell counts were normalized by region volume (mm^3^). To remove outlier counts for specific regions in the mesoSPIM cohort resulting from technical artifacts (internal shadows, etc), quantifications were done for each illumination separately, and a median absolute deviation (MAD) filter of +/− 4 was applied for each region per genotype. After filtering, counts from each illumination for each region were averaged to acquire a single count per region per sample. Statistical significance for K27M-flag versus Ctl comparisons for each region was assessed using the Mann-Whitney U test with Benjamini-Hochberg adjustment applied.

#### Basis pontis area quantification

For basis pontis area quantification (Supplementary Fig. 5), CNPase-stained cleared brain images were cropped to the basis pontis in Arivis Vision4D. Matched images were captured for the coronal, sagittal, and horizontal planes at the broadest/deepest point of the basis pontis for each sample. The basis pontis was then manually annotated in Fiji for the coronal and sagittal planes based on CNPase architecture, and the area of each was measured.

#### Visualization and rendering

Data from brains scanned with the Ultramicroscope II were visualized and rendered using Arivis Vision4D software (Zeiss) (Fig. 2a, b and Supplementary Videos 1, 2) and Fiji (v1.54p) (Supplementary Fig. 5). Broad region atlas heatmaps (Fig. 2c–e and Supplementary Fig. 4b, c) were generated with Paraview (v5.13.3)^65^.

### In vivo magnetic resonance imaging (MRI)

Neuroimaging was completed on a 7T Bruker ClinScan small animal MRI imager (Bruker Biospin MRI GmbH) at the Center for In Vivo Imaging and Therapeutics (CIVIT), which is equipped with an integrated animal handling system. DTI and T2w images were acquired. Prior to the imaging session, the mice were anesthetized in a chamber with 3% isoflurane in oxygen delivered at a rate of 0.5 L/min, and this anesthesia level was subsequently maintained through nose-cone delivery, with a mixture of 1–2% isoflurane in oxygen, delivered at the same flow rate. To ensure the animals’ well-being and stability, a heated bed with warm water circulation and a physiologic monitoring system were employed to monitor the respiratory rate. Volume measurements (brain, hippocampus, lateral ventricle, cortex, pons, cerebellum and 4^th^ ventricle) were obtained by manually segmenting the regions and computing volumes using OsiriX (v5.7, Pixmeo, Switzerland) or Fiji. White matter was segmented as described previously^66^. DTI was quantified as described previously^67^.

### RNA extraction

For in vitro OL-lineage cultures, 100,000-200,000 OPCs were plated in a 6-well plate in either proliferation or differentiation medium and collected at specified timepoints. 1 mL Trizol was added directly to each well, and RNA was extracted following the standard Trizol protocol except the final 75% EtOH wash was repeated three times. RNA was resuspended in 15 µl nuclease-free water. For snap-frozen brainstem and cortices, 1 mL of Trizol was added to the tissue and homogenized through trituration through a series of small-gauge needle syringes. RNA extraction was performed with the standard Trizol protocol, with the final 75% EtOH wash repeated three times and resuspended in 20–40 µl nuclease-free water.

### Quantitative reverse transcriptase PCR (qRT-PCR)

cDNA was generated from the standard Superscript III (Invitrogen) protocol with 100 ng of total RNA. 1 μl of each 20 μl cDNA reaction was utilized per qRT-PCR reaction. qPCR was performed with Sybr Green Master Mix (Applied Biosystems) and run on a CFX96 Real Time PCR System (BioRad). Gene expression was normalized to *Actb* mRNA expression for each sample. Primers used for qPCR reactions:

**Table:** 

Primer name	Sequence
Sybr ActB F	ACC CAC ACT GTG CCC ATC TAC
Sybr ActB R	AGC CAA GTC CAG ACG CAG G
Sybr Gfap F	GAC ATC GAG ATC GCC ACC TA
Sybr Gfap R	GTG CTT TTG CCC CCT CG
Sybr Aldh1l1 F	TTTGGCAAAGACCTGGGAGA
Sybr Aldh1l1 R	GCTAAGTCACCAGACAGGGT
Sybr Slc1a3 F	CGCTCGCTAAGCTGTTACTC
Sybr Slc1a3 R	TTGGTGTTAGAGAGGACAACTTTT
Sybr Rbfox3 F	ACGCTGAACCCGTTGAAAA
Sybr Rbfox3 R	CTGTTGGGGTGTCGTCTACT

### RNA sequencing

All library preparation and sequencing were performed by the Hartwell Center at St. Jude Children’s Research Hospital. RNA was quantified using the Quant-iT RiboGreen RNA assay (ThermoFisher) and quality checked by the 2100 Bioanalyzer RNA 6000 Nano assay (Agilent) or 4200 TapeStation High Sensitivity RNA ScreenTape assay (Agilent) prior to library generation. Libraries were prepared from total RNA with the TruSeq Stranded Total RNA Library Prep Kit according to the manufacturer’s instructions (Illumina, PN 20020599). Libraries were analyzed for insert size distribution using the 2100 BioAnalyzer High Sensitivity kit (Agilent), 4200 TapeStation D1000 ScreenTape assay (Agilent), or 5300 Fragment Analyzer NGS fragment kit (Agilent). Libraries were quantified using the Quant-iT PicoGreen dsDNA assay (ThermoFisher) or by low-pass sequencing with a MiSeq nano kit (Illumina). Paired-end 100-cycle sequencing was performed on a NovaSeq 6000 (Illumina).

### Bulk RNA-seq analyses

#### Quantification and differential expression

Transcript-level count estimates were generated with the nf-core RNA-seq pipeline (v3.11.2)^68^ using selective alignment via salmon (v1.10.1)^69^ with an mm10 decoy-aware transcriptome and Gencode vM25 annotations with the --seqBias and --gcBias parameters. Transcript counts were collapsed to the gene level with the tximport (v1.34.0) R package^70^. Differential expression analyses were performed with DESeq2 (v1.46.0)^71^ using an adjusted *P* value threshold of 0.05 and a fold change effect size threshold of 1.1 with culture ID included in the model to account for unwanted variation. Visualizations were created with the dittoSeq (v1.18.0)^72^ and ggplot2^73^ (v3.5.1) R packages using either log_2_ normalized counts or variance stabilization transformed counts as appropriate.

#### Enrichment analyses

Pre-ranked gene set enrichment analyses were performed with the fgsea (v1.32.0) R package^74^ and the MSigDB (v7.5.1) gene sets^75^ retrieved via the msigdbr R package or published/derived glioma-related gene sets (Supplementary Data 9) using an adjusted *P* value of 0.05 as a significance threshold. The signed test statistic was used to rank genes. Pathway and geneset hypergeometric enrichment analyses of differentially expressed genes were performed with the clusterProfiler (v4.14.4) R package^76^ using only genes that passed DESeq2’s independent filtering thresholds as background and an adjusted *P* value of 0.05 as a significance threshold. Semantic comparisons of GO terms were performed with the calculateSemMatrix function from the GOSemSim (v2.32.0) R package^77^ using “method = ‘Rel’” and reduced to clusters with the reduceSimMatrix function with a threshold of 0.7. For visualization of the clusters, the top -log_10_(adjusted *P* value) for the cluster was used as the score and the top 5 most frequent words in the term names (with common stopwords removed) in the cluster were used as labels.

#### Weighted gene correlation network analysis

Weighted gene correlation network analysis was performed with the WGCNA (v1.73) R package^78^. A power of 5 was used to generate an unsigned network with modules of at least 20 genes using “bicor” correlation. A mergeCutHeight of 0.25 was used to merge closely related modules. Modules were correlated with factorized categorical traits via Pearson correlation, from which Student asymptomatic *P* values were calculated for each correlation with the corPvalueStudent function. K-means clustering with *k* = 2 was used to split differentiation-associated modules into OPC/iOL-associated and NFOL/mOL-associated gene sets.

#### Cell type deconvolution and differentiation indexing

Cell type proportion deconvolution was performed with MuSiC2 (installed from commit f21fe67) using the control samples from the brainstem scRNA dataset generated from this study as a reference^79^. Only cells from the Pdgfra+ oligodendrocyte lineage were used. Default parameters were used. A differentiation index for each sample was derived by assigning a numerical index to each cell type (1: cycling OPC; 2: OPC; 3: COP; 4: NFOL; 5: mOL) and taking the sum of each index multiplied by its corresponding proportion. Indices were visualized with the dittoViz (v1.0.1) and ggpubr (v0.6.0) R packages. The Mann–Whitney U test was used to determine significant differences for the differentiation index between conditions at each timepoint. *P* values were adjusted with the Benjamini-Hochberg procedure for tests performed within each timepoint.

#### Overlap of genes with published studies

To annotate genes in Fig. 7b with the impact of K27M on their expression in human H3 K27M DMG models, published data from^24,42^ were used. Silveira et al., 2019 utilized H3 K27M DMG xenograft models with or without K27M knockdown via shRNA. Krug et al., 2019 utilized H3 K27M DMG cell lines with or without CRISPR-Cas9 deletion of K27M. Differentially expressed genes for each study were collected from either supplementary data (Supplementary Data 9^42^;) or the original analyses^24^. Differentially expressed genes were defined as those with an adjusted *P* value < 0.05. Gene promoter bivalency for^24^ was defined as described. Human to mouse ortholog conversion was performed with the babelgene (v22.9; https://cran.r-project.org/web/packages/babelgene/index.html) R package with at least 3 sources of supporting evidence.

### Single-cell RNA sequencing

P4, P14, and P21 hGFAP-Cre;K27M and littermate control mouse brainstems were dissociated into a single-cell suspension by enzymatic dissociation in a mixture of 1% activated Papain in Hibernate-A™ media with 0.16 mg/ml *N*-acetyl cysteine and 0.012 mg of DNase. Samples were agitated and/or gently pipetted every 5–10 min throughout the 15–30 min digestion at 37 °C. The dissociated cells were diluted further in Hibernate-A, filtered through a 40 µm strainer and centrifuged at 150–250 *g* for 5 min at 4 °C. Cell pellets were resuspended in fresh Hibernate-A with Calcein Red (1:1000; Invitrogen) and sorted for live (Calcein Red+) cells using an On-chip cell sorter into Hibernate-A. Cells were processed for single-cell RNA-seq using the Chromium Single Cell 3ʹ GEM, Library & Gel Bead Kit v3.1 (PN-1000121) following the protocol as written with a target recovery of 5000 cells per sample. Emulsions were generated on a Chromium Controller. All sequencing was carried out by the Hartwell Center at St. Jude Children’s Research Hospital. Libraries were sequenced on a NovaSeq 6000 (Illumina) according to manufacturer instructions for 3’ v3.1 libraries – 28 × 8 × 0 × 91.

### scRNA-seq analyses

#### Alignment, gene quantification, ambient RNA removal, QC, and normalization

FASTQs were aligned to mm10 with cellranger (v8.0.1) using default parameters and create-bam set to “true”^80^. Read counts were adjusted to remove those expected to stem from ambient RNA using cellbender (v0.3.0) with default parameters^81^. For each sample, per-cell QC metrics were calculated with the scuttle R package (v1.16.0)^82^. Outlier cells were identified as those over 3 median absolute deviations away from the median. Outlier cells and those with >10% mitochondrial reads, fewer than 500 genes detected, or fewer than 1500 total counts were removed. Doublets were detected and removed for each sample with scDblFinder (v1.20.0) with default parameters^83^. Genes detected in fewer than 10 cells were removed. Log-transformed expression values normalized for library size for each cell were calculated with scuttle.

#### Dimensionality reduction & integration

PCA on the top 3000 most variable genes was performed with the scater (v1.34.0) R package^82^. Samples were integrated using PCA embeddings with the harmony (v1.2.1) R package^84^ using the sample identifier and assigned bins for mitochondrial read percentage and total count as covariates to be removed. UMAP on the harmony embeddings was performed with the uwot (v0.2.2) R package with *k* = 30 and min.dist = 0.3^85^.

#### Cell type annotations

Cell cycle phase classification was performed with the cyclone function from the scran (v1.34.0) R package^86^. To assign cell types with a high level of granularity, cells were overclustered via walktrap clustering with *k* = 5 performed on the integrated harmony embeddings. Markers for each cluster were identified with scran’s scoreMarkers function with a log-fold change threshold of 0.1. Cell types were assigned to each cluster based on known markers. Visualizations were created with the dittoSeq (v1.18.0), dittoViz (v1.0.1), and VizModules (v0.1.1) R packages^72,87^.

#### Cell type marker identification

For comparison of cell type signatures between genotypes, the top 100 markers for each cell type in the control samples were identified with the scoreMarkers function from the scran R package, blocking for sample differences, and ranking by the mean AUC for each cell type (Supplementary Data 4).

#### H3.3 K27M induction assessment

Genotyping for H3.3 K27M in each cell was performed with vartrix (v1.1.22; https://github.com/10XGenomics/vartrix) with default parameters.

#### Differential expression

Differential expression analysis was performed using pseudobulk profiles for each sample for each cell type at each age, generated with the aggregateAcrossCells function from the scuttle (v1.16.0) R package. Pseudobulk samples generated from 10 or fewer cells were removed. Pseudobulk profiles between the H3.3 control and H3.3 K27M conditions for each age and cell type were then compared with the pseudoBulkDGE function from the scran (v1.34.0) R package^86^ using empirical Bayes quasi-likelihood F-tests after fitting a quasi-likelihood negative binomial generalized log-linear model. *P* values were adjusted using the Benjamini-Hochberg method for multiple test correction. A robust H3.3 K27M signature was derived by collecting all H3.3 K27M up-regulated genes (FDR < 0.05; logFC > 0.2) for each comparison and counting the frequency of their occurrence across cell types and ages. Those that occurred three or more times (*n* = 156; Supplementary Data 6) were retained in the signature. *H3-3a* and *Prm1* were removed from the signature due to their artifactual inclusion because of the *loxP*-STOP-*loxP* cassette (expression from only 1 allele in littermate controls (no Cre)) and Cre construct (harbors *Prm1* promoter), respectively.

#### Gene signature scoring and comparison

Gene signature scores were generated with AddModuleScore with default parameters from the Seurat (v5.1.0) R package^88^. Scores were averaged for each cell type, and sample with samples with fewer than 10 cells of a given cell type were dropped. The *t* test was used to determine significant differences in average scores between conditions for each cell type at each timepoint with the ggpubr (v0.6.0) R package (https://rpkgs.datanovia.com/ggpubr/). *P* values were adjusted with the Benjamini-Hochberg procedure for tests performed within each timepoint.

#### Pseudotime ordering and trajectory analysis

Pseudotime ordering was performed for the oligodendrocyte lineage with the slingshot (v2.12.0) R package^89^ with preOPCs used as the start cluster and mOLs used as the end cluster, and parameters extend = “n”, stretch = 0, shrink = FALSE, dist.method = “mnn”. Differential testing over pseudotime between genotypes for the oligodendrocyte lineage was performed with the tradeSeq (v1.18.0) R package^90^. A generalized additive model was fit to the 5000 most variable genes in the oligodendrocyte lineage cells with the fitGAM function with nknots = 6 and conditions set to H3 status in each cell (K27M or ctl). The conditionTest function was then used to determine differential expression between conditions along pseudotime with l2fc = log_2_(1.25) to minimize very low effect size differences. *P* values were adjusted for multiple testing using Benjamini-Hochberg adjustment. Genes with an adjusted *P* value < 0.05 were considered significantly differentially expressed (Supplementary Data 5).

#### Differential abundance testing

Differences in cell type abundance were performed with edgeR (v4.4.0)^91^. Samples were limited to cells in the neuronal, glial, or progenitor lineages prior to calculating cell type proportions in each sample to limit the composition effects of variable inclusion of fibroblast cells stemming from meninges. These proportions were then tested for significant differences between genotypes for each tissue at each timepoint using empirical Bayes quasi-likelihood F-tests after fitting a quasi-likelihood negative binomial generalized log-linear model. *P* values were adjusted using the Benjamini-Hochberg method for multiple test correction.

### ChIP-seq

ChIP-seq was carried out as in^10^ with some modifications. Briefly, control and K27M OPC cultures were harvested and snap frozen as pellets. Frozen pellets were resuspended and fixed at room temperature 1% paraformaldehyde in PBS for 10 min. Fixation was quenched, nuclei were isolated and then sheared in a Covaris M220 ultrasonicator (Microtube, 75 W, 5% duty cycle, 200 cycles/burst, 7 min). Sheared chromatin from Drosophila melanogaster S2 cells (ATCC, CRL-1963) was added to the mammalian chromatin prior to ChIP and chromatin containing spike-in was kept as an input control. ChIP reactions were carried out exactly as detailed in^10^ using H3K27me3 (Cell Signaling, Cat# 9733, Clone# C36B11, lot 8) and H3K4me3 (Cell Signaling, Cat# 9751, Clone# C42D8, lot 8) antibodies. Libraries were generated using up to 5 ng of chromatin following a modified Kapa Hyper Prep protocol in which Illumina TruSeq DNA UD Index amounts were scaled to the starting material (1.5 µM index for up to 5 ng input, 0.75 µM for less than 2.5 ng and the number of cycles for amplification for each sample was determined with a real-time PCR pretest). Single-end 100-cycle sequencing was performed on a NovaSeq 6000 (Illumina).

### ATAC sequencing

ATAC-seq was carried out following the Omni-ATAC protocol^92^ with the following modifications. Prior to the transposition reaction, control and K27M OPC cultures were harvested and snap frozen as pellets and nuclei were isolated using Protocol 2 in the 10X Genomics Demonstrated Protocol (*CG000212_SingleCellATAC_Nuclei_Isolation_MouseBrain_DemonstratedProtocol_RevB)*. Following final amplification, the column cleanup was eliminated and replaced with SPRISelect bead cleanup (1.6×) with final elution in 20 ul of Qiagen Buffer EB. Paired-end 75-cycle sequencing was performed on a NextSeq 2000 (Illumina).

### ChIP-seq and ATAC-seq analyses

#### Alignment and peak calling

Alignment to mm10 (and dm6 for H3K27me3 samples), QC, and peak calling were performed with the nf-core ChIP-seq (v2.0.0) or ATAC-seq (v2.1.2) pipelines^68^ using the ENCODE mm10 v2 blacklist^93^ to remove reads overlapping problematic genomic regions. Broad or narrow peak calling via MACS2^94^ (v2.2.7.1) was used as appropriate after alignment with bwa^95^ (v0.7.17-r1188) with default parameters.

#### Peak filtering and consensus set generation

Consensus peaks for each histone mark or ATAC were derived for replicates of each genotype and origin tissue using rmspc^96^ (v1.12.0) with replicateType = “Biological”, c = 2, and keep = FALSE. For H3K4me3 and ATAC-seq, stringencyThreshold = 1e-10 and weakThreshold = 1e-6, while H3K27me3 used stringencyThreshold = 1e-8 and weakThreshold = 1e-4.

#### Promoter status assignment

Promoter regions (2000 bp upstream, 1000 bp downstream) were retrieved for each transcript from the Gencode M25 annotations, also used for RNA-seq analyses. Promoters were limited to those of expressed genes in the undifferentiated samples (0-day timepoint) from the in vitro differentiation RNA-seq data. Promoter status was determined via consensus H3K4me3/H3K27me3 peak overlap for each brain region from control samples only, given broad global depletion of H3K27me3 in K27M samples. To account for multiple TSS for any given gene, a priority assignment order was used: bivalent, H3K4me3 only, H3K27me3 only and negative. For example, if the promoter of any TSS for a gene was bivalent, the gene was assigned bivalent status. A chi-square test of independence was used to test observed versus expected frequencies for genes up- and down-regulated by K27M in samples from each origin tissue.

#### Differential binding/accessibility comparison at gene promoters

Differential comparisons of ChIP and ATAC-seq signal at promoter regions were performed with csaw^97^ (v1.40.0) and edgeR^98^ (v4.4.0). Briefly, read counts were quantified for gene promoters (2 kb upstream, 1 kb downstream of TSS). For ATAC-seq, duplicate reads and reads from fragments over 1 kb were discarded. Reads with mapping quality <20 were discarded. For H3K4me3 ChIP-seq and ATAC-seq, normalization offsets were calculated by performing a loess fit to normalize for trended biases and library size. For H3K27me3 comparisons between control and K27M samples, normalization factors were calculated as (# Drosophila reads in IP / total reads in IP) / (# Drosophila reads in input / total reads in input). For H3K27me3 comparisons between control samples from different origin tissues, a loess fit was used. Testing was performed using empirical Bayes quasi-likelihood F-tests after fitting a quasi-likelihood negative binomial generalized log-linear model, accounting for matched cultures between conditions where possible.

#### bigWig generation and signal visualization

bigWig files were generated for each sample using deeptools (v3.5.4) bamCoverage^99^. For ATAC-seq, the following parameters were used: --binSize 20, reads overlapping ENCODE blacklist regions ignored, --normalizeUsing CPM, --minMappingQuality 20, and --ignoreDuplicates. For H3K4me3, the following parameters were used: --binSize 20, reads overlapping ENCODE blacklist regions ignored, --normalizeUsing CPM, --minMappingQuality 20, and –extendReads 250. For H3K27me3, the same parameters as H3K4me3 were used, but an additional scaling factor was applied, calculated as the ratio of the read percentages assigned to Drosophila by bbsplit in the input vs IP for each sample. All the scaling factors were then divided by the largest to scale them all relative to 1. Group-wise bigWigs were made for each mark, genotype, and origin tissue with deepTools’ bigwigAverage. Gene regions were visualized with deepTools’ computeMatrix scale-regions with each gene scaled to 10 kb length with 3 kb upstream of the TSS and downstream of the TES followed by deepTools’ plotHeatmap of the resulting matrix.

### Quantification and statistical analysis

All data and analyses, except for the -omics analyses and OPC growth curve analysis (see Method details), were completed using GraphPad Prism 10.0. The data, error bars, “n” numbers, and statistical tests for each experiment are specified in the text and/or figure legends.

### Reporting summary

Further information on research design is available in the Nature Portfolio Reporting Summary linked to this article.

## Supplementary information

Supplementary Information

Description of Additional Supplementary Files

Supplementary Video 1

Supplementary Video 2

Supplementary Data 1

Supplementary Data 2

Supplementary Data 3

Supplementary Data 4

Supplementary Data 5

Supplementary Data 6

Supplementary Data 7

Supplementary Data 8

Supplementary Data 9

Supplementary Data 10

Supplementary Data 11

Supplementary Data 12

Reporting Summary

Transparent Peer Review file

## Data Availability

All sequencing data generated for this project were deposited at GEO under accessions GSE290816, GSE290817, GSE310474, and GSE310472. H3 K27M DMG xenograft data used from^24^ are available at GEO under accession GSE115875. Primary human DMG data used from^43^ is available at EGA under accession EGAS00001000192. K27M-flag, WT-flag, and K27M mice without a tag are available upon request to the Baker lab. Due to the large size of cleared whole-brain imaging files (>250 GB per sample), full regional quantifications are provided as supplemental and source data, with full imaging datasets available on request. Source data are provided with this paper and on figshare at 10.6084/m9.figshare.31004440.

## References

[1] Khuong-Quang, D. A. et al. K27M mutation in histone H3.3 defines clinically and biologically distinct subgroups of pediatric diffuse intrinsic pontine gliomas. *Acta Neuropathol.* **124**, 439–447 (2012). 22661320 10.1007/s00401-012-0998-0 PMC3422615

[2] Wu, G. et al. Somatic histone H3 alterations in pediatric diffuse intrinsic pontine gliomas and non-brainstem glioblastomas. *Nat. Genet* **44**, 251–253 (2012). 22286216 10.1038/ng.1102 PMC3288377

[3] Ocasio, J. K., Budd, K. M., Roach, J. T., Andrews, J. M. & Baker, S. J. Oncohistones and disrupted development in pediatric-type diffuse high-grade glioma. *Cancer Metastasis Rev.* **42**, 367–388 (2023). 37119408 10.1007/s10555-023-10105-2 PMC10441521

[4] Hoffman, L. M. et al. Spatial genomic heterogeneity in diffuse intrinsic pontine and midline high-grade glioma: implications for diagnostic biopsy and targeted therapeutics. *Acta Neuropathol. Commun.* **4**, 1 (2016). 26727948 10.1186/s40478-015-0269-0 PMC4700584

[5] Nikbakht, H. et al. Spatial and temporal homogeneity of driver mutations in diffuse intrinsic pontine glioma. *Nat. Commun.* **7**, 11185 (2016). 27048880 10.1038/ncomms11185 PMC4823825

[6] Vinci, M. et al. Functional diversity and cooperativity between subclonal populations of pediatric glioblastoma and diffuse intrinsic pontine glioma cells. *Nat. Med.* **24**, 1204–1215 (2018). 29967352 10.1038/s41591-018-0086-7 PMC6086334

[7] Arunachalam, S. et al. Convergent evolution and multi-wave clonal invasion in H3 K27-altered diffuse midline gliomas treated with a PDGFR inhibitor. *Acta Neuropathol. Commun.* **10**, 80 (2022). 35642016 10.1186/s40478-022-01381-0 PMC9153212

[8] Cordero, F. J. et al. Histone H3.3K27M represses p16 to accelerate gliomagenesis in a murine model of DIPG. *Mol. Cancer Res.* **15**, 1243–1254 (2017). 28522693 10.1158/1541-7786.MCR-16-0389 PMC5581686

[9] Funato, K., Major, T., Lewis, P. W., Allis, C. D. & Tabar, V. Use of human embryonic stem cells to model pediatric gliomas with H3.3K27M histone mutation. *Science* **346**, 1529–1533 (2014). 25525250 10.1126/science.1253799 PMC4995593

[10] Larson, J. D. et al. Histone H3.3 K27M accelerates spontaneous brainstem glioma and drives restricted changes in bivalent gene expression. *Cancer Cell* **35**, 140–155 e147 (2019). 30595505 10.1016/j.ccell.2018.11.015 PMC6570409

[11] Mohammad, F. et al. EZH2 is a potential therapeutic target for H3K27M-mutant pediatric gliomas. *Nat. Med*. **23**, 483–492 (2017). 28263309 10.1038/nm.4293

[12] Patel, S. K. et al. Generation of diffuse intrinsic pontine glioma mouse models by brainstem-targeted in utero electroporation. *Neuro Oncol.* **22**, 381–392 (2020). 31638150 10.1093/neuonc/noz197 PMC7442382

[13] Pathania, M. et al. H3.3(K27M) cooperates with Trp53 loss and PDGFRA gain in mouse embryonic neural progenitor cells to induce invasive high-grade gliomas. *Cancer Cell* **32**, 684–700 e689 (2017). 29107533 10.1016/j.ccell.2017.09.014 PMC5687892

[14] Haag, D. et al. H3.3-K27M drives neural stem cell-specific gliomagenesis in a human iPSC-derived model. *Cancer Cell* **39**, 407–422 e413 (2021). 33545065 10.1016/j.ccell.2021.01.005

[15] Kim, G. B. et al. Rapid generation of somatic mouse mosaics with locus-specific, stably integrated transgenic elements. *Cell* **179**, 251–267 e224 (2019). 31539496 10.1016/j.cell.2019.08.013 PMC6934691

[16] Lindquist, R. A. et al. Identification of proliferative progenitors associated with prominent postnatal growth of the pons. *Nat. Commun.* **7**, 11628 (2016). 27188978 10.1038/ncomms11628 PMC4873968

[17] Tate, M. C. et al. Postnatal growth of the human pons: a morphometric and immunohistochemical analysis. *J. Comp. Neurol.* **523**, 449–462 (2015). 25307966 10.1002/cne.23690 PMC4270924

[18] Nagaraja, S. et al. Histone variant and cell context determine H3K27M reprogramming of the enhancer landscape and oncogenic state. *Mol. Cell* **76**, 965–980 e912 (2019). 31588023 10.1016/j.molcel.2019.08.030 PMC7104854

[19] Monje, M. et al. Hedgehog-responsive candidate cell of origin for diffuse intrinsic pontine glioma. *Proc. Natl. Acad. Sci. USA*. **108**, 4453–4458 (2011). 21368213 10.1073/pnas.1101657108 PMC3060250

[20] Nagaraja, S. et al. Transcriptional dependencies in diffuse intrinsic pontine glioma. *Cancer Cell* **31**, 635–652 e636 (2017). 28434841 10.1016/j.ccell.2017.03.011 PMC5462626

[21] Filbin, M. G. et al. Developmental and oncogenic programs in H3K27M gliomas dissected by single-cell RNA-seq. *Science* **360**, 331–335 (2018). 29674595 10.1126/science.aao4750 PMC5949869

[22] Jessa, S. et al. K27M in canonical and noncanonical H3 variants occurs in distinct oligodendroglial cell lineages in brain midline gliomas. *Nat. Genet.* **54**, 1865–1880 (2022). 36471070 10.1038/s41588-022-01205-w PMC9742294

[23] Harutyunyan, A. S. et al. H3K27M induces defective chromatin spread of PRC2-mediated repressive H3K27me2/me3 and is essential for glioma tumorigenesis. *Nat. Commun.* **10**, 1262 (2019). 30890717 10.1038/s41467-019-09140-x PMC6425035

[24] Silveira, A. B. et al. H3.3 K27M depletion increases differentiation and extends latency of diffuse intrinsic pontine glioma growth in vivo. *Acta Neuropathol.* **137**, 637–655 (2019). 30770999 10.1007/s00401-019-01975-4 PMC6546611

[25] Marques, S. et al. Transcriptional convergence of oligodendrocyte lineage progenitors during development. *Dev. Cell* **46**, 504–517 e507 (2018). 30078729 10.1016/j.devcel.2018.07.005 PMC6104814

[26] Knowles, J. K., Batra, A., Xu, H. & Monje, M. Adaptive and maladaptive myelination in health and disease. *Nat. Rev. Neurol.* **18**, 735–746 (2022). 36376595 10.1038/s41582-022-00737-3

[27] Vigano, F., Mobius, W., Gotz, M. & Dimou, L. Transplantation reveals regional differences in oligodendrocyte differentiation in the adult brain. *Nat. Neurosci.* **16**, 1370–1372 (2013). 23995069 10.1038/nn.3503

[28] Spitzer, S. O. et al. Oligodendrocyte progenitor cells become regionally diverse and heterogeneous with age. *Neuron* **101**, 459–471 e455 (2019). 30654924 10.1016/j.neuron.2018.12.020 PMC6372724

[29] Andrade, A. F., Chen, C. C. L. & Jabado, N. Oncohistones in brain tumors: the soil and seed. *Trends Cancer* **9**, 444–455 (2023). 36933956 10.1016/j.trecan.2023.02.003 PMC11075889

[30] Zhuo, L. et al. hGFAP-cre transgenic mice for manipulation of glial and neuronal function in vivo. *Genesis* **31**, 85–94 (2001). 11668683 10.1002/gene.10008

[31] Perens, J. et al. An optimized mouse brain atlas for automated mapping and quantification of neuronal activity using iDISCO+ and light sheet fluorescence microscopy. *Neuroinformatics* **19**, 433–446 (2021). 33063286 10.1007/s12021-020-09490-8 PMC8233272

[32] Wang, J. et al. EED-mediated histone methylation is critical for CNS myelination and remyelination by inhibiting WNT, BMP, and senescence pathways. *Sci. Adv.* **6**, eaaz6477 (2020). 32851157 10.1126/sciadv.aaz6477 PMC7423366

[33] Wang, W. et al. PRC2 acts as a critical timer that drives oligodendrocyte fate over astrocyte identity by repressing the notch pathway. *Cell Rep.* **32**, 108147 (2020). 32937136 10.1016/j.celrep.2020.108147 PMC8070886

[34] Foran, D. R. & Peterson, A. C. Myelin acquisition in the central nervous system of the mouse revealed by an MBP-Lac Z transgene. *J. Neurosci.* **12**, 4890–4897 (1992). 1281497 10.1523/JNEUROSCI.12-12-04890.1992 PMC6575777

[35] Sturrock, R. R. Myelination of the mouse corpus callosum. *Neuropathol. Appl. Neurobiol.* **6**, 415–420 (1980). 7453945 10.1111/j.1365-2990.1980.tb00219.x

[36] van Tilborg, E. et al. Origin and dynamics of oligodendrocytes in the developing brain: Implications for perinatal white matter injury. *Glia* **66**, 221–238 (2018). 29134703 10.1002/glia.23256 PMC5765410

[37] Emery, B. & Dugas, J. C. Purification of oligodendrocyte lineage cells from mouse cortices by immunopanning. *Cold Spring Harb. Protoc.* **2013**, 854–868 (2013). 24003195 10.1101/pdb.prot073973

[38] Bressan, R. B. et al. Regional identity of human neural stem cells determines oncogenic responses to histone H3.3 mutants. *Cell Stem Cell* **28**, 877–893 e879 (2021). 33631116 10.1016/j.stem.2021.01.016 PMC8110245

[39] Magni, M. et al. Brain regional identity and cell type specificity landscape of human cortical organoid models. *Int J. Mol. Sci.* **23**, 13159 (2022). 36361956 10.3390/ijms232113159 PMC9654943

[40] Philippidou, P. & Dasen, J. S. Hox genes: choreographers in neural development, architects of circuit organization. *Neuron* **80**, 12–34 (2013). 24094100 10.1016/j.neuron.2013.09.020 PMC3835187

[41] Boncinelli, E., Mallamaci, A. & Muzio, L. Genetic control of regional identity in the developing vertebrate forebrain. *Novartis Found. Symp.* **228**, 53–61 (2000). 10929316 10.1002/0470846631.ch5

[42] Krug, B. et al. Pervasive H3K27 acetylation leads to ERV expression and a therapeutic vulnerability in H3K27M gliomas. *Cancer Cell* **35**, 782–797 e788 (2019). 31085178 10.1016/j.ccell.2019.04.004 PMC6521975

[43] Wu, G. et al. The genomic landscape of diffuse intrinsic pontine glioma and pediatric non-brainstem high-grade glioma. *Nat. Genet* **46**, 444–450 (2014). 24705251 10.1038/ng.2938 PMC4056452

[44] Marangon, D., Boccazzi, M., Lecca, D. & Fumagalli, M. Regulation of oligodendrocyte functions: targeting lipid metabolism and extracellular matrix for myelin repair. *J. Clin. Med.* **9**, 470 (2020). 32046349 10.3390/jcm9020470 PMC7073561

[45] Khandker, L. et al. Cholesterol biosynthesis defines oligodendrocyte precursor heterogeneity between the brain and the spinal cord. *Cell Rep.* **38**, 110423 (2022). 35235799 10.1016/j.celrep.2022.110423 PMC8988216

[46] Lourenco, T. et al. Modulation of oligodendrocyte differentiation and maturation by combined biochemical and mechanical cues. *Sci. Rep.* **6**, 21563 (2016). 26879561 10.1038/srep21563 PMC4754901

[47] Yamada, M., Iwase, M., Sasaki, B. & Suzuki, N. The molecular regulation of oligodendrocyte development and CNS myelination by ECM proteins. *Front. Cell Dev. Biol.* **10**, 952135 (2022). 36147746 10.3389/fcell.2022.952135 PMC9488109

[48] Liu, I. et al. The landscape of tumor cell states and spatial organization in H3-K27M mutant diffuse midline glioma across age and location. *Nat. Genet.* **54**, 1881–1894 (2022). 36471067 10.1038/s41588-022-01236-3 PMC9729116

[49] Siletti, K. et al. Transcriptomic diversity of cell types across the adult human brain. *Science* **382**, eadd7046 (2023). 37824663 10.1126/science.add7046

[50] Jakovcevski, I., Filipovic, R., Mo, Z., Rakic, S. & Zecevic, N. Oligodendrocyte development and the onset of myelination in the human fetal brain. *Front. Neuroanat.* **3**, 5 (2009). 19521542 10.3389/neuro.05.005.2009 PMC2694674

[51] Choe, Y., Pleasure, S. J. & Mira, H. Control of adult neurogenesis by short-range morphogenic-signaling molecules. *Cold Spring Harb. Perspect. Biol.* **8**, a018887 (2015). 26637286 10.1101/cshperspect.a018887 PMC4772099

[52] Bruschi, M. et al. Diffuse midline glioma invasion and metastasis rely on cell-autonomous signaling. *Neuro Oncol.* **26**, 553–568 (2024). 37702430 10.1093/neuonc/noad161 PMC10912010

[53] Huchede, P. et al. BMP2 and BMP7 cooperate with H3.3K27M to promote quiescence and invasiveness in pediatric diffuse midline gliomas. *Elife* **12**, RP91313 (2024). 39373720 10.7554/eLife.91313 PMC11458179

[54] Barron, T. et al. GABAergic neuron-to-glioma synapses in diffuse midline gliomas. *Nature* **639**, 1060–1068 (2025). 39972132 10.1038/s41586-024-08579-3 PMC11946904

[55] Taylor, K. R. et al. Glioma synapses recruit mechanisms of adaptive plasticity. *Nature* **623**, 366–374 (2023). 37914930 10.1038/s41586-023-06678-1 PMC10632140

[56] Venkatesh, H. S. et al. Electrical and synaptic integration of glioma into neural circuits. *Nature* **573**, 539–545 (2019). 31534222 10.1038/s41586-019-1563-y PMC7038898

[57] Liu, J. et al. Chromatin landscape defined by repressive histone methylation during oligodendrocyte differentiation. *J. Neurosci.* **35**, 352–365 (2015). 25568127 10.1523/JNEUROSCI.2606-14.2015 PMC4287153

[58] Boshans, L. L. et al. The chromatin environment around interneuron genes in oligodendrocyte precursor cells and their potential for interneuron reprograming. *Front Neurosci.* **13**, 829 (2019). 31440130 10.3389/fnins.2019.00829 PMC6694778

[59] Schindelin, J. et al. Fiji: an open-source platform for biological-image analysis. *Nat. Methods* **9**, 676–682 (2012). 22743772 10.1038/nmeth.2019 PMC3855844

[60] Bankhead, P. et al. QuPath: open source software for digital pathology image analysis. *Sci. Rep.* **7**, 16878 (2017). 29203879 10.1038/s41598-017-17204-5 PMC5715110

[61] Park, Y. G. et al. Protection of tissue physicochemical properties using polyfunctional crosslinkers. *Nat. Biotechnol.* **37**, 73–83 (2018). 10.1038/nbt.4281 PMC6579717 30556815

[62] Vladimirov, N. et al. Benchtop mesoSPIM: a next-generation open-source light-sheet microscope for cleared samples. *Nat. Commun.* **15**, 2679 (2024). 38538644 10.1038/s41467-024-46770-2 PMC10973490

[63] Berg, S. et al. ilastik: interactive machine learning for (bio)image analysis. *Nat. Methods* **16**, 1226–1232 (2019). 31570887 10.1038/s41592-019-0582-9

[64] Tustison, N. J. et al. The ANTsX ecosystem for quantitative biological and medical imaging. *Sci. Rep.* **11**, 9068 (2021). 33907199 10.1038/s41598-021-87564-6 PMC8079440

[65] Ahrens, J., Geveci, B. & Law, C. in *Visualization Handbook* (eds Charles D. Hansen & Chris R. Johnson) 717-731 (Butterworth-Heinemann, 2005).

[66] Tang, T. T. et al. A comprehensive preclinical assessment of late-term imaging markers of radiation-induced brain injury. *Neurooncol. Adv.* **1**, vdz012 (2019). 31608330 10.1093/noajnl/vdz012 PMC6777502

[67] Song, R., Glass, J. O. & Reddick, W. E. Modified diffusion tensor image processing pipeline for archived studies of patients with leukoencephalopathy. *J. Magn. Reson. Imaging* **54**, 997–1008 (2021). 33856092 10.1002/jmri.27636 PMC8628478

[68] Ewels, P. A. et al. The nf-core framework for community-curated bioinformatics pipelines. *Nat. Biotechnol.* **38**, 276–278 (2020). 32055031 10.1038/s41587-020-0439-x

[69] Patro, R., Duggal, G., Love, M. I., Irizarry, R. A. & Kingsford, C. Salmon provides fast and bias-aware quantification of transcript expression. *Nat. Methods* **14**, 417–419 (2017). 28263959 10.1038/nmeth.4197 PMC5600148

[70] Soneson, C., Love, M. I. & Robinson, M. D. Differential analyses for RNA-seq: transcript-level estimates improve gene-level inferences. *F1000Res* **4**, 1521 (2015). 26925227 10.12688/f1000research.7563.1 PMC4712774

[71] Love, M. I., Huber, W. & Anders, S. Moderated estimation of fold change and dispersion for RNA-seq data with DESeq2. *Genome Biol.* **15**, 550 (2014). 25516281 10.1186/s13059-014-0550-8 PMC4302049

[72] Bunis, D. G., Andrews, J., Fragiadakis, G. K., Burt, T. D. & Sirota, M. dittoSeq: universal user-friendly single-cell and bulk RNA sequencing visualization toolkit. *Bioinformatics* **36**, 5535–5536 (2021). 33313640 10.1093/bioinformatics/btaa1011 PMC8016464

[73] Wickham, H. *ggplot2**:**Elegant Graphics for**Data Analysis*. (Springer International Publishing, 2016).

[74] Korotkevich, G. et al. Fast gene set enrichment analysis. Preprint at *bioRxiv*10.1101/060012 (2021).

[75] Liberzon, A. et al. Molecular signatures database (MSigDB) 3.0. *Bioinformatics* **27**, 1739–1740 (2011). 21546393 10.1093/bioinformatics/btr260 PMC3106198

[76] Yu, G., Wang, L. G., Han, Y. & He, Q. Y. ClusterProfiler: an R package for comparing biological themes among gene clusters. *OMICS* **16**, 284–287 (2012). 22455463 10.1089/omi.2011.0118 PMC3339379

[77] Yu, G. et al. GOSemSim: an R package for measuring semantic similarity among GO terms and gene products. *Bioinformatics* **26**, 976–978 (2010). 20179076 10.1093/bioinformatics/btq064

[78] Langfelder, P. & Horvath, S. WGCNA: an R package for weighted correlation network analysis. *BMC Bioinforma.* **9**, 559 (2008). 10.1186/1471-2105-9-559 PMC2631488 19114008

[79] Fan, J. et al. MuSiC2: cell-type deconvolution for multi-condition bulk RNA-seq data. *Brief. Bioinform* **23**, bbac430 (2022). 36208175 10.1093/bib/bbac430 PMC9677503

[80] Zheng, G. X. et al. Massively parallel digital transcriptional profiling of single cells. *Nat. Commun.* **8**, 14049 (2017). 28091601 10.1038/ncomms14049 PMC5241818

[81] Fleming, S. J. et al. Unsupervised removal of systematic background noise from droplet-based single-cell experiments using CellBender. *Nat. Methods* **20**, 1323–1335 (2023). 37550580 10.1038/s41592-023-01943-7

[82] McCarthy, D. J., Campbell, K. R., Lun, A. T. & Wills, Q. F. Scater: pre-processing, quality control, normalization and visualization of single-cell RNA-seq data in R. *Bioinformatics* **33**, 1179–1186 (2017). 28088763 10.1093/bioinformatics/btw777 PMC5408845

[83] Germain, P. L., Lun, A., Garcia Meixide, C., Macnair, W. & Robinson, M. D. Doublet identification in single-cell sequencing data using scDblFinder. *F1000Res* **10**, 979 (2021). 35814628 10.12688/f1000research.73600.1 PMC9204188

[84] Kramer, D. et al. A fast, sensitive and accurate high-resolution melting (HRM) technology-based assay to screen for common K-ras mutations. *Cell Oncol.* **31**, 161–167 (2009). 19478384 10.3233/CLO-2009-0466 PMC4618824

[85] McInnes, L., Healy, J., Saul, N. & Großerger, L. UMAP: uniform manifold approximation and projection. *J. Open Source Softw.*10.21105/joss.00861 (2018).

[86] Lun, A. T., Bach, K. & Marioni, J. C. Pooling across cells to normalize single-cell RNA sequencing data with many zero counts. *Genome Biol.* **17**, 75 (2016). 27122128 10.1186/s13059-016-0947-7 PMC4848819

[87] Andrews, J. & Martin, J. VizModules: flexible, interactive ‘shiny’ modules for almost any plot. 10.32614/CRAN.package.VizModules (2026).

[88] Andreatta, M. & Carmona, S. J. UCell and pyUCell: single-cell gene signature scoring for R and Python. *Bioinformatics* **42**, btag055 (2026). 41665985 10.1093/bioinformatics/btag055 PMC12925249

[89] Street, K. et al. Slingshot: cell lineage and pseudotime inference for single-cell transcriptomics. *BMC Genomics* **19**, 477 (2018). 29914354 10.1186/s12864-018-4772-0 PMC6007078

[90] Van den Berge, K. et al. Trajectory-based differential expression analysis for single-cell sequencing data. *Nat. Commun.* **11**, 1201 (2020). 32139671 10.1038/s41467-020-14766-3 PMC7058077

[91] Chen, Y., Lun, A. T. & Smyth, G. K. From reads to genes to pathways: differential expression analysis of RNA-Seq experiments using Rsubread and the edgeR quasi-likelihood pipeline. *F1000Res* **5**, 1438 (2016). 27508061 10.12688/f1000research.8987.1 PMC4934518

[92] Corces, M. R. et al. An improved ATAC-seq protocol reduces background and enables interrogation of frozen tissues. *Nat. Methods* **14**, 959–962 (2017). 28846090 10.1038/nmeth.4396 PMC5623106

[93] Amemiya, H. M., Kundaje, A. & Boyle, A. P. The ENCODE blacklist: identification of problematic regions of the genome. *Sci. Rep.* **9**, 9354 (2019). 31249361 10.1038/s41598-019-45839-z PMC6597582

[94] Zhang, Y. et al. Model-based analysis of ChIP-Seq (MACS). *Genome Biol.* **9**, R137 (2008). 18798982 10.1186/gb-2008-9-9-r137 PMC2592715

[95] Li, H. & Durbin, R. Fast and accurate short read alignment with Burrows-Wheeler transform. *Bioinformatics* **25**, 1754–1760 (2009). 19451168 10.1093/bioinformatics/btp324 PMC2705234

[96] Jalili, V., Matteucci, M., Masseroli, M. & Morelli, M. J. Using combined evidence from replicates to evaluate ChIP-seq peaks. *Bioinformatics* **31**, 2761–2769 (2015). 25957351 10.1093/bioinformatics/btv293

[97] Lun, A. T. & Smyth, G. K. csaw: a Bioconductor package for differential binding analysis of ChIP-seq data using sliding windows. *Nucleic Acids Res.* **44**, e45 (2016). 26578583 10.1093/nar/gkv1191 PMC4797262

[98] Chen, Y., Chen, L., Lun, A. T. L., Baldoni, P. L. & Smyth, G. K. edgeR v4: powerful differential analysis of sequencing data with expanded functionality and improved support for small counts and larger datasets. *Nucleic Acids Res.* **53**, gkaf018 (2025). 39844453 10.1093/nar/gkaf018 PMC11754124

[99] Ramirez, F. et al. deepTools2: a next-generation web server for deep-sequencing data analysis. *Nucleic Acids Res.* **44**, W160–165 (2016). 27079975 10.1093/nar/gkw257 PMC4987876

