# Supplementary material for: Cell autonomous regional differences in oligodendrocyte lineage development and responses to oncohistone H3.3 K27M
Source: Nat Commun. 2026 Aug 26;17:8811. doi: 10.1038/s41467-026-76468-6 (PMC13518833; doi:10.1038/s41467-026-76468-6)
Supplement: Supplementary file 1 [file 41467_2026_76468_MOESM17_ESM.pdf]

Corresponding author(s): Suzanne Baker

Last updated by author(s): 06/22/2026

Reporting Summary

Nature Portfolio wishes to improve the reproducibility of the work that we publish. This form provides structure for consistency and transparency in reporting. For further information on Nature Portfolio policies, see our Editorial Policies and the Editorial Policy Checklist.

Statistics

For all statistical analyses, confirm that the following items are present in the figure legend, table legend, main text, or Methods section.

n/a	Confirmed	
☐	☒	The exact sample size (n) for each experimental group/condition, given as a discrete number and unit of measurement
☐	☒	A statement on whether measurements were taken from distinct samples or whether the same sample was measured repeatedly
☐	☒	The statistical test(s) used AND whether they are one- or two-sided<br>*Only common tests should be described solely by name; describe more complex techniques in the Methods section.*
☐	☒	A description of all covariates tested
☐	☒	A description of any assumptions or corrections, such as tests of normality and adjustment for multiple comparisons
☐	☒	A full description of the statistical parameters including central tendency (e.g. means) or other basic estimates (e.g. regression coefficient) AND variation (e.g. standard deviation) or associated estimates of uncertainty (e.g. confidence intervals)
☐	☒	For null hypothesis testing, the test statistic (e.g. F, t, r) with confidence intervals, effect sizes, degrees of freedom and P value noted<br>*Give P values as exact values whenever suitable.*
☒	☐	For Bayesian analysis, information on the choice of priors and Markov chain Monte Carlo settings
☐	☒	For hierarchical and complex designs, identification of the appropriate level for tests and full reporting of outcomes
☐	☒	Estimates of effect sizes (e.g. Cohen's d, Pearson's r), indicating how they were calculated

Our web collection on statistics for biologists contains articles on many of the points above.

Software and code

Policy information about availability of computer code

Data collection	BD FACSDiva v8.0.1
Data analysis	BD FACSDiva v8.0.1<br>nf-core RNA-seq pipeline (v3.11.2)<br>salmon (v1.10.1)<br>tximport (v1.34.0)<br>DESeq2 (v1.46.0)<br>dittoSeq (v1.18.0)<br>ggplot2 (v3.5.1)<br>dittoViz (v1.0.1)<br>fgsea (v1.32.0)<br>MSigDB (v7.5.1)<br>clusterProfiler (v4.14.4)<br>GOSemSim (v2.32.0)<br>WGCNA (v1.73)<br>MuSiC2 (commit f21fe67)<br>ggpubr (v0.6.0)<br>babelgene (v22.9)<br>cellranger (v8.0.1)

cellbender (v0.3.0)
scuttle (v1.16.0)
scDblFinder (v1.20.0)
scater (v1.34.0)
scran (v1.34.0)
samtools (v1.15.1)
bedtools (v2.30.0)
deeptools (v3.5.1)
homer (v4.11)
picard (2.27.4-SNAPSHOT)
R (v4.4.1)
harmony (v1.2.1)
uwot (v0.2.2)
vartrix (v1.1.22)
Seurat (v5.1.0)
slingshot (v2.12.0)
tradeSeq (v1.18.0)
edgeR (v4.4.0)
nf-core ChIP-seq pipeline (v2.0.0)
nf-core ATAC-seq pipeline (v2.1.2)
MACS2 (v2.2.7.1)
bwa (v0.7.17-r1188)
VizModules (v0.1.1)
rmspc (v1.12.0)
csaw (v1.40.0)
deepTools (v3.5.4)
GraphPad Prism 10.0
QuPath (v0.6.0)
Paraview (v5.13.3)
Arivis Vision4D
ImageJ/Fiji (v1.54p)
OsiriX (v5.7)
R (v4.4.3)

For manuscripts utilizing custom algorithms or software that are central to the research but not yet described in published literature, software must be made available to editors and reviewers. We strongly encourage code deposition in a community repository (e.g. GitHub). See the Nature Portfolio guidelines for submitting code & software for further information.

Data

Policy information about availability of data

All manuscripts must include a data availability statement. This statement should provide the following information, where applicable:

- Accession codes, unique identifiers, or web links for publicly available datasets
- A description of any restrictions on data availability
- For clinical datasets or third party data, please ensure that the statement adheres to our policy

All sequencing data generated for this project were deposited at GEO under accessions GSE290816 [https://www.ncbi.nlm.nih.gov/geo/query/acc.cgi?acc=GSE290816], GSE290817 [https://www.ncbi.nlm.nih.gov/geo/query/acc.cgi?acc=GSE290817], GSE310474 [https://www.ncbi.nlm.nih.gov/geo/query/acc.cgi?acc=GSE310474], and GSE310472 [https://www.ncbi.nlm.nih.gov/geo/query/acc.cgi?acc=GSE310472]. H3 K27M DMG xenograft data used from 24 are available at GEO under accession GSE115875 [https://www.ncbi.nlm.nih.gov/geo/query/acc.cgi?acc=GSE115875]. Primary human DMG data used from 44 is available at EGA under accession EGAS00001000192 [https://ega-archive.org/studies/EGAS00001000192]. Source data are provided with this paper.

Research involving human participants, their data, or biological material

Policy information about studies with human participants or human data. See also policy information about sex, gender (identity/presentation), and sexual orientation and race, ethnicity and racism.

Reporting on sex and gender

Use the terms sex (biological attribute) and gender (shaped by social and cultural circumstances) carefully in order to avoid confusing both terms. Indicate if findings apply to only one sex or gender; describe whether sex and gender were considered in study design; whether sex and/or gender was determined based on self-reporting or assigned and methods used.
Provide in the source data disaggregated sex and gender data, where this information has been collected, and if consent has been obtained for sharing of individual-level data; provide overall numbers in this Reporting Summary. Please state if this information has not been collected.
Report sex- and gender-based analyses where performed, justify reasons for lack of sex- and gender-based analysis.

Reporting on race, ethnicity, or other socially relevant groupings

Please specify the socially constructed or socially relevant categorization variable(s) used in your manuscript and explain why they were used. Please note that such variables should not be used as proxies for other socially constructed/relevant variables (for example, race or ethnicity should not be used as a proxy for socioeconomic status).
Provide clear definitions of the relevant terms used, how they were provided (by the participants/respondents, the researchers, or third parties), and the method(s) used to classify people into the different categories (e.g. self-report, census or

	administrative data, social media data, etc.) *Please provide details about how you controlled for confounding variables in your analyses.*
Population characteristics	*Describe the covariate-relevant population characteristics of the human research participants (e.g. age, genotypic information, past and current diagnosis and treatment categories). If you filled out the behavioural & social sciences study design questions and have nothing to add here, write "See above."*
Recruitment	*Describe how participants were recruited. Outline any potential self-selection bias or other biases that may be present and how these are likely to impact results.*
Ethics oversight	*Identify the organization(s) that approved the study protocol.*

Note that full information on the approval of the study protocol must also be provided in the manuscript.

Field-specific reporting

Please select the one below that is the best fit for your research. If you are not sure, read the appropriate sections before making your selection.

[x] Life sciences [ ] Behavioural & social sciences [ ] Ecological, evolutionary & environmental sciences

For a reference copy of the document with all sections, see nature.com/documents/nr-reporting-summary-flat.pdf

Life sciences study design

All studies must disclose on these points even when the disclosure is negative.

Sample size	For immunofluorescence imaging experiments, sample size calculations were performed for Wilcoxon Mann-Whitney U Tests for an effect size of 1, standard deviation of 0.5, and desired 70% power This yielded 6 samples per group. For sequencing experiments, power is highly dependent on the dataset and sequencing depth. For RNA-seq, 9 samples per group were used, as this provides >80% power in several datasets (https://pmc.ncbi.nlm.nih.gov/articles/PMC4201821/). For ChIP and ATAC-seq, at least 2 samples per group were used, as recommended by ENCODE.
Data exclusions	ChIP-seq samples were excluded if they were of low quality (poor enrichment, few peaks, etc). Certain measurements for cleared whole brain quantifications were removed if outliers (+/- 4 MAD) due to technical artifacts (internal shadows).
Replication	Conclusions made by this study were supported by multiple orthogonal experimental approaches, e.g. in vivo imaging with in vitro differentiation experiments followed by RNA-seq and additional imaging. No non-replicable findings were included.
Randomization	Samples were allocated into experimental groups based on their genotype. Where possible, batch was provided as a covariate in model design to mitigate technical effects.
Blinding	Investigators were not blinded, as most imaging quantifications were performed objectively by uniformly applied computational methods.

Reporting for specific materials, systems and methods

We require information from authors about some types of materials, experimental systems and methods used in many studies. Here, indicate whether each material, system or method listed is relevant to your study. If you are not sure if a list item applies to your research, read the appropriate section before selecting a response.

Materials & experimental systems

n/a	Involved in the study	
[ ]	[x]	Antibodies
[ ]	[x]	Eukaryotic cell lines
[x]	[ ]	Palaeontology and archaeology
[ ]	[x]	Animals and other organisms
[x]	[ ]	Clinical data
[x]	[ ]	Dual use research of concern
[x]	[ ]	Plants

Methods

n/a	Involved in the study	
[ ]	[x]	ChIP-seq
[ ]	[x]	Flow cytometry
[ ]	[x]	MRI-based neuroimaging

Antibodies

Antibodies used	OLIG2 pAb, R&D Systems, Cat # AF2418, https://www.rndsystems.com/products/human-mouse-rat-olig2-antibody_af2418<br>OLIG2 mAb, Millipore, Cat # MABN50, clone 211F1.1, https://www.sigmaaldrich.com/US/en/product/mm/mabn50?srsltid=AfmBOopTcxtZyR_NUidvODPpYyeZnDJ6PtzJbyOIXapkEc0IHFLLNGks<br>OLIG2 pAb, Millipore, Cat # AB9610, https://www.sigmaaldrich.com/US/en/product/mm/ab9610?srsltid=AfmBOor11t2a7dSSHtlYS4qdeNx0DTqHbYGjlD-n3XgZdGoaheFv8CED<br>CC1 (APC) mAb, Calbiochem/MilliporeSigma, Cat # OP80, clone Ab-7, https://www.sigmaaldrich.com/US/en/product/mm/op80?

srsltid=AfmBOopX_8WIeRIMF3bxGgZtm3DviH8LlHAbsdh--CT7nbHVOTFqaQM_
MBP mAb, Millipore, Cat # NE1019, clone SMI-99, https://www.sigmaaldrich.com/US/en/product/mm/ne1019?srsltid=AfmBOopQAARnmzbSvHxlZQQojj6J0ScfCgRN-MUKHvyFuHdTW26mby1v
MBP pAb, Aves Labs, Cat # MBP, https://www.antibodiesinc.com/products/anti-myelin-basic-protein-antibody-mbp
GFP pAb, Abcam, Cat # Ab290, https://www.abcam.com/en-us/products/primary-antibodies/gfp-antibody-ab290
CD31 mAb, BD Biosciences, Cat # 553370, clone MEC13.3, https://www.bdbiosciences.com/en-us/products/reagents/flow-cytometry-reagents/research-reagents/single-color-antibodies-ruo/purified-rat-anti-mouse-cd31.553370?tab=product_details
CNPase pAb, EnCor, Cat # RPCA-CNP, https://encorbio.com/products/rabbit-polyclonal-antibody-to-cnp-cat-rpca-cnp
CD140a mAb, BD Pharmingen, Cat # 558774, Clone # APA5, https://www.bdbiosciences.com/en-at/products/reagents/western-blotting-and-molecular-reagents/western-blot-reagents/purified-rat-anti-mouse-cd140a.558774?tab=antibody_details
NG2 pAb, Millipore, Cat # AB5320, https://www.sigmaaldrich.com/US/en/product/mm/ab5320
CNPase mAb, Millipore, Cat # MAB326, Clone # 11-5B, https://www.sigmaaldrich.com/US/en/product/mm/mab326?srsltid=AfmBOood_ppSjJMz_SRqI0_Sp3or6n2PXQ-3nY6Vcdyb0GvoI5A-OfFu
H3K27me3 mAb , Cell Signaling, Cat # 9733, Clone # C36B11, lot 8, https://www.cellsignal.com/products/primary-antibodies/tri-methyl-histone-h3-lys27-c36b11-rabbit-monoclonal-antibody/9733
H3K4me3 mAb, Cell Signaling, Cat # 9751, Clone # C42D8, lot 8, https://www.cellsignal.com/products/primary-antibodies/tri-methyl-histone-h3-lys4-c42d8-rabbit-monoclonal-antibody/9751
DCX mAb, Santa Cruz, Cat # SC271390, Clone # E-6, https://www.scbt.com/p/doublecortin-antibody-e-6
Ki67 mAb, Cell Signaling, Cat # 12202, Clone # D3B5, https://www.cellsignal.com/products/primary-antibodies/ki-67-d3b5-rabbit-monoclonal-antibody-ihc-formulated/12202?srsltid=AfmBOoqmyxJQClmwIzYM69kf2IEPc0OnzliGmqrqYt1yNf77Q5yEH-2t
Ki67, mAb, Abcam, Cat # Ab16667, Clone # SP6, https://www.abcam.com/en-us/products/primary-antibodies/ki67-antibody-sp6-ab16667
Flag, mAb, Cell Signaling, Cat # 14793, Clone # D6W5B, https://www.cellsignal.com/products/primary-antibodies/dykddddk-tag-d6w5b-rabbit-monoclonal-antibody-binds-to-same-epitope-as-sigma-aldrich-anti-flag-m2-antibody/14793?srsltid=AfmBOoq1OAb40fLlr94j9yXOZa6Rjydjqv-1Eygtrb26duyl6HCo5eDb
Flag, mAb, Sigma, Cat # F1804, Clone # M2, https://www.sigmaaldrich.com/US/en/product/sigma/f1804?srsltid=AfmBOopIHSsqGy-_EIArVxBSvmKCh7S2xgDUwsDLnUh5BRPPvRDMuMKA
NeuN, mAb, Millipore, Cat # MAB377, Clone # A60, https://www.sigmaaldrich.com/US/en/product/mm/mab377?srsltid=AfmBOopgzb1tz1bfPj-t_snxK9_Wn2lDbu4AdhqzIcysIqfC0e3NJWE0
GFAP, mAb, Cell Signaling, Cat # 3670, Clone # GA5, https://www.cellsignal.com/products/primary-antibodies/gfap-ga5-mouse-monoclonal-antibody/3670?srsltid=AfmBOootvhCI3NTgwihh6gdsI1jJchT1zAI9xuPNT6ernL-QZJgRC_Qz

Validation	Manufacturer & published validation experiments and notes available via websites listed.

Eukaryotic cell lines

Policy information about cell lines and Sex and Gender in Research

Cell line source(s)	Drosophila D.MEL S2<br>(ATCC cat.# CRL-1963, Biovest part # OO.763 or OO.627)
Authentication	Lines were received directly as cross-linked cell pellets from Biovest. Authentication as Drosophila was confirmed via sequencing and subsequent alignment to the Drosophila genome.
Mycoplasma contamination	Mycoplasma was not detected in the source material (ATCC). No in-house culturing was performed.
Commonly misidentified lines<br>(See ICLAC register)	*Name any commonly misidentified cell lines used in the study and provide a rationale for their use.*

Animals and other research organisms

Policy information about studies involving animals; ARRIVE guidelines recommended for reporting animal research, and Sex and Gender in Research

Laboratory animals	P0, P4, P7, P14, P21, P35, and up to 60 week hGFAP-Cre K27M, K27M-flag, and WT-flag mus musculus with FVB background. hGFAP-Cre mice were obtained from the Jackson Laboratory (Strain #004600) and maintained on FVB/N background.
Wild animals	The study did not involve wild animals.
Reporting on sex	Male and female mice were utilized for all experiments and analyses.
Field-collected samples	The study did not involve samples collected from the field.
Ethics oversight	All experiments involving animals followed protocols (Protocol # 278-100611) approved by the Institutional Animal Care and Use Committee at St. Jude Children's Research Hospital and were in compliance with institutional guidelines.

Note that full information on the approval of the study protocol must also be provided in the manuscript.

Plants

Seed stocks	*Report on the source of all seed stocks or other plant material used. If applicable, state the seed stock centre and catalogue number. If plant specimens were collected from the field, describe the collection location, date and sampling procedures.*
Novel plant genotypes	*Describe the methods by which all novel plant genotypes were produced. This includes those generated by transgenic approaches, gene editing, chemical/radiation-based mutagenesis and hybridization. For transgenic lines, describe the transformation method, the number of independent lines analyzed and the generation upon which experiments were performed. For gene-edited lines, describe the editor used, the endogenous sequence targeted for editing, the targeting guide RNA sequence (if applicable) and how the editor was applied.*
Authentication	*Describe any authentication procedures for each seed stock used or novel genotype generated. Describe any experiments used to assess the effect of a mutation and, where applicable, how potential secondary effects (e.g. second site T-DNA insertions, mosiacism, off-target gene editing) were examined.*

ChIP-seq

Data deposition

☒ Confirm that both raw and final processed data have been deposited in a public database such as GEO.

☒ Confirm that you have deposited or provided access to graph files (e.g. BED files) for the called peaks.

Data access links
May remain private before publication.

https://www.ncbi.nlm.nih.gov/geo/query/acc.cgi?acc=GSE310474
Data has been made public.

Files in database submission

Raw reads (FASTQ), narrowPeak or broadPeak, and CPM (or spike-in normalized for H3K27me3) bigWig files provided for every sample.

OB59K_OPC-H3K27me3_S2_L001_R1_001.fastq.gz
OB59K_OPC-H3K27me3-INPUT_S49_L002_R1_001.fastq.gz
OB59K_OPC-H3K4me3_S30_L001_R1_001.fastq.gz
OB59K_OPC-H3K4me3-INPUT_S80_L002_R1_001.fastq.gz
OB59W_OPC-H3K27me3_S1_L001_R1_001.fastq.gz
OB59W_OPC-H3K27me3-INPUT_S47_L002_R1_001.fastq.gz
OB59W_OPC-H3K4me3_S28_L001_R1_001.fastq.gz
OB59W_OPC-H3K4me3-INPUT_S78_L002_R1_001.fastq.gz
OB61K_OPC-H3K27me3_S13_L001_R1_001.fastq.gz
OB61K_OPC-H3K27me3-INPUT_S61_L002_R1_001.fastq.gz
OB61K_OPC-H3K4me3_S41_L001_R1_001.fastq.gz
OB61K_OPC-H3K4me3-INPUT_S91_L002_R1_001.fastq.gz
OB61W_OPC-H3K27me3_S11_L001_R1_001.fastq.gz
OB61W_OPC-H3K27me3-INPUT_S59_L002_R1_001.fastq.gz
OB61W_OPC-H3K4me3_S39_L001_R1_001.fastq.gz
OB61W_OPC-H3K4me3-INPUT_S89_L002_R1_001.fastq.gz
OC59K_OPC-H3K27me3_S5_L001_R1_001.fastq.gz
OC59K_OPC-H3K27me3-INPUT_S6_L001_R1_001.fastq.gz
OC59K_OPC-H3K4me3_S21_L001_R1_001.fastq.gz
OC59K_OPC-H3K4me3-INPUT_S22_L001_R1_001.fastq.gz
OC59W_OPC-H3K27me3_S1_L001_R1_001.fastq.gz
OC59W_OPC-H3K27me3-INPUT_S2_L001_R1_001.fastq.gz
OC59W_OPC-H3K4me3_S17_L001_R1_001.fastq.gz
OC59W_OPC-H3K4me3-INPUT_S18_L001_R1_001.fastq.gz
OC60K_OPC-H3K27me3_S5_L001_R1_001.fastq.gz
OC60K_OPC-H3K27me3-INPUT_S53_L002_R1_001.fastq.gz
OC60K_OPC-H3K4me3_S33_L001_R1_001.fastq.gz
OC60K_OPC-H3K4me3-INPUT_S83_L002_R1_001.fastq.gz
OC60W_OPC-H3K27me3_S3_L001_R1_001.fastq.gz
OC60W_OPC-H3K27me3-INPUT_S51_L002_R1_001.fastq.gz
OC60W_OPC-H3K4me3_S32_L001_R1_001.fastq.gz
OC60W_OPC-H3K4me3-INPUT_S82_L002_R1_001.fastq.gz
OC61K_OPC-H3K27me3_S9_L001_R1_001.fastq.gz
OC61K_OPC-H3K27me3-INPUT_S57_L002_R1_001.fastq.gz
OC61K_OPC-H3K4me3_S37_L001_R1_001.fastq.gz
OC61K_OPC-H3K4me3-INPUT_S87_L002_R1_001.fastq.gz
OC61W_OPC-H3K27me3_S7_L001_R1_001.fastq.gz
OC61W_OPC-H3K27me3-INPUT_S55_L002_R1_001.fastq.gz
OC61W_OPC-H3K4me3_S35_L001_R1_001.fastq.gz
OC61W_OPC-H3K4me3-INPUT_S85_L002_R1_001.fastq.gz

OC59K_OPC-H3K27me3_S5_L002_R1_001.fastq.gz
OC59K_OPC-H3K27me3-INPUT_S6_L002_R1_001.fastq.gz
OC59K_OPC-H3K4me3_S21_L002_R1_001.fastq.gz
OC59K_OPC-H3K4me3-INPUT_S22_L002_R1_001.fastq.gz
OC59W_OPC-H3K27me3_S1_L002_R1_001.fastq.gz
OC59W_OPC-H3K27me3-INPUT_S2_L002_R1_001.fastq.gz
OC59W_OPC-H3K4me3_S17_L002_R1_001.fastq.gz
OC59W_OPC-H3K4me3-INPUT_S18_L002_R1_001.fastq.gz
OB59K_OPC-H3K27me3.broadPeak
OB59K_OPC-H3K4me3.narrowPeak
OB59W_OPC-H3K27me3.broadPeak
OB59W_OPC-H3K4me3.narrowPeak
OB61K_OPC-H3K27me3.broadPeak
OB61K_OPC-H3K4me3.narrowPeak
OB61W_OPC-H3K27me3.broadPeak
OB61W_OPC-H3K4me3.narrowPeak
OC59K_OPC-H3K27me3.broadPeak
OC59K_OPC-H3K4me3.narrowPeak
OC59W_OPC-H3K27me3.broadPeak
OC59W_OPC-H3K4me3.narrowPeak
OC60K_OPC-H3K27me3.broadPeak
OC60K_OPC-H3K4me3.narrowPeak
OC60W_OPC-H3K27me3.broadPeak
OC60W_OPC-H3K4me3.narrowPeak
OC61K_OPC-H3K27me3.broadPeak
OC61K_OPC-H3K4me3.narrowPeak
OC61W_OPC-H3K27me3.broadPeak
OC61W_OPC-H3K4me3.narrowPeak
OB59K_OPC-H3K27me3-AB4.scaled.bw
OB59K_OPC-H3K4me3-AB7.mLb.clN.sorted.bw
OB59W_OPC-H3K27me3-AB4.scaled.bw
OB59W_OPC-H3K4me3-AB7.mLb.clN.sorted.bw
OB61K_OPC-H3K27me3-AB4.scaled.bw
OB61K_OPC-H3K4me3-AB7.mLb.clN.sorted.bw
OB61W_OPC-H3K27me3-AB4.scaled.bw
OB61W_OPC-H3K4me3-AB7.mLb.clN.sorted.bw
OC59K_OPC-H3K27me3-AB4.scaled.bw
OC59K_OPC-H3K4me3-AB7.mLb.clN.sorted.bw
OC59W_OPC-H3K27me3-AB4.scaled.bw
OC59W_OPC-H3K4me3-AB7.mLb.clN.sorted.bw
OC60K_OPC-H3K27me3-AB4.scaled.bw
OC60K_OPC-H3K4me3-AB7.mLb.clN.sorted.bw
OC60W_OPC-H3K27me3-AB4.scaled.bw
OC60W_OPC-H3K4me3-AB7.mLb.clN.sorted.bw
OC61K_OPC-H3K27me3-AB4.scaled.bw
OC61K_OPC-H3K4me3-AB7.mLb.clN.sorted.bw
OC61W_OPC-H3K27me3-AB4.scaled.bw
OC61W_OPC-H3K4me3-AB7.mLb.clN.sorted.bw

Genome browser session
(e.g. UCSC)

No longer applicable, bigWig and peak files provided via GEO upload.

Methodology

Replicates

2-3 biological replicates per group (location + genotype).

Sequencing depth

100 bp single-end sequencing was performed on all samples.

Sample total_passed duplicates_passed
OC59W_OPC-H3K27me3-AB4 118024943 27582870
OC59W_OPC-H3K4me3-AB7 123930938 28928507
OC59W_OPC-INPUT 89784579 28931057
OC59K_OPC-H3K27me3-AB4 99731597 21390832
OC59K_OPC-H3K4me3-AB7 113204650 26999664
OC59K_OPC-INPUT 95295007 26430517
OB59W_OPC-H3K27me3-AB4 77210544 20474485
OB59W_OPC-H3K4me3-AB7 35253791 8400667
OB59W_OPC-INPUT 150094982 31292157
OB59K_OPC-H3K27me3-AB4 59055085 16133547

OB59K_OPC-H3K4me3-AB7 69481664 19040472
OB59K_OPC-INPUT 172714191 32596268
OC60W_OPC-H3K27me3-AB4 52387642 10953102
OC60W_OPC-H3K4me3-AB7 78749420 58667959
OC60W_OPC-INPUT 124640302 23602706
OC60K_OPC-H3K27me3-AB4 68432767 17871294
OC60K_OPC-H3K4me3-AB7 40797208 10253994
OC60K_OPC-INPUT 122148017 23412645
OC61W_OPC-H3K27me3-AB4 75505404 18581100
OC61W_OPC-H3K4me3-AB7 43600166 18319757
OC61W_OPC-INPUT 110278898 20492601
OC61K_OPC-H3K27me3-AB4 36203038 9173559
OC61K_OPC-H3K4me3-AB7 43240625 12350813
OC61K_OPC-INPUT 88207265 17095183
OB61W_OPC-H3K27me3-AB4 36087194 8663130
OB61W_OPC-H3K4me3-AB7 65929233 15953759
OB61W_OPC-INPUT 156914708 33711714
OB61K_OPC-H3K27me3-AB4 38514734 12637681
OB61K_OPC-H3K4me3-AB7 37191409 8877439
OB61K_OPC-INPUT 76430297 21244918

Antibodies

H3K27me3 mAb , Cell Signaling, Cat # 9733, Clone # C36B11, lot 8, https://www.cellsignal.com/products/primary-antibodies/tri-methyl-histone-h3-lys27-c36b11-rabbit-monoclonal-antibody/9733
H3K4me3 mAb, Cell Signaling, Cat # 9751, Clone # C42D8, lot 8, https://www.cellsignal.com/products/primary-antibodies/tri-methyl-histone-h3-lys4-c42d8-rabbit-monoclonal-antibody/9751

Peak calling parameters

Raw reads were aligned to mm10 with bwa mem (v0.7.17-r1188) with default parameters. Peaks were called with MACS2 (v2.2.7.1).

For broad peak calling, the following parameters were used:
macs2 callpeak --keep-dup all --broad --broad-cutoff 0.1 --gsize 2652783500

For narrow peak calling, the following parameters were used:
macs2 callpeak --keep-dup all --gsize 2652783500

With input controls utilized in both cases.

Data quality

For quantification, reads with mapping quality < 20 or overlapping ENCODE blacklist regions were removed. Poor performing samples with <20000 (H3K4me3) or <4000 (H3K27me3) peaks were removed.

Software

Data was processed with the nf-core ChIP-seq pipeline (v2.0.0), which utilizes:
bwa mem (v0.7.17-r1188)
MACS2 (v2.2.7.1)
samtools (v1.15.1)
bedtools (v2.30.0)
deeptools (v3.5.1)
homer (v4.11)
picard (2.27.4-SNAPSHOT)

Downstream analyses utilized:
R (v4.4.1)
edgeR (v4.4.0)
csaw (v1.40.0)
rmspc (1.12.0)
deeptools (v3.5.4)
clusterProfiler (v4.14.4)

Flow Cytometry

Plots

Confirm that:

- [x] The axis labels state the marker and fluorochrome used (e.g. CD4-FITC).
- [x] The axis scales are clearly visible. Include numbers along axes only for bottom left plot of group (a 'group' is an analysis of identical markers).
- [x] All plots are contour plots with outliers or pseudocolor plots.
- [x] A numerical value for number of cells or percentage (with statistics) is provided.

Methodology

Sample preparation: Early passage OPCs were plated at a density of 100,000 cells/T25 flask in 3 mL proliferation medium and allowed to attach overnight. To label S-phase cells, 3 mL of proliferation medium containing 20 uM EdU was added to the OPCs (final concentration of 10 uM EdU) and incubated for 1 hr. Cells were detached with Accutase and then fixed and permeabilized as noted above (Immunocytochemistry), and ClickIT reactions performed with the Click-iT Plus EdU Alexa Fluor 488 Kit for Flow Cytometry per the kit protocol (Invitrogen). Cells were resuspended in 100 ul of FxCycle PI RNase Staining Solution (Invitrogen) and incubated on ice for at least 30 minutes prior to flow cytometry assessment on a Becton Dickinson Aria Fusion.

Instrument: Becton Dickinson Aria Fusion

Software: BD FACSDiva 8.0.1

Cell population abundance: Cell populations in G0/G1, G2/M, or S phase of the cell cycle were assessed by propium iodide and EdU staining. Abundances varied by sample, but a typical proportion was ~55-60% G0/G1, ~15-20% G2/M, and ~20-30% S phase.

Gating strategy: Cells were first gated on propium iodide-A and -W to select live, single cells. Cells were then gated by EdU Alexa488-A and propium iodide-A to label cells as S phase (EdU+), G0/G1 (PI-lo; EdU-), or G2/M (PI-hi; EdU-) with gates set with unstained and single stain samples. Examples are provided in the Source Data.

☒ Tick this box to confirm that a figure exemplifying the gating strategy is provided in the Supplementary Information.

Magnetic resonance imaging

Experimental design

Design type: Resting state

Design specifications: Single scan

Behavioral performance measures: None

Acquisition

Imaging type(s): Structural

Field strength: 9.4T

Sequence & imaging parameters: T2-weighted Rapid Acquisition with Refocused Echoes (RARE) sequences in the coronal (TR/TE = 2000/20.4 ms, matrix size = 256 x 256, field of view = 20 mm x 20 mm, slice thickness = 0.5 mm, number of slices = 16) and axial (TR/TE = 2500/23 ms, matrix size = 256 x 256, field of view = 20 mm x 20 mm, slice thickness = 0.5 mm, number of slices = 32) orientations.

Area of acquisition: Brain.

Diffusion MRI: ☐ Used ☒ Not used

Preprocessing

Preprocessing software: No preprocessing software was used.

Normalization: Data were not normalized.

Normalization template: No normalization template was used.

Noise and artifact removal: No noise and artifact removal was used.

Volume censoring: No volume censoring was used.

Statistical modeling & inference

Model type and settings: No models were used.

Effect(s) tested: No effects were tested.

Specify type of analysis: ☒ Whole brain ☐ ROI-based ☐ Both

Statistic type for inference (See Eklund et al. 2016): No inference was used.

Correction: No corrections were made.

Models & analysis

n/a	Involved in the study
☒	☐ Functional and/or effective connectivity
☒	☐ Graph analysis
☒	☐ Multivariate modeling or predictive analysis
