# Supplementary material for: Cell autonomous regional differences in oligodendrocyte lineage development and responses to oncohistone H3.3 K27M
Source: Nat Commun. 2026 Aug 26;17:8811. doi: 10.1038/s41467-026-76468-6 (PMC13518833; doi:10.1038/s41467-026-76468-6)
Supplement: Supplementary file 2 [file 41467_2026_76468_MOESM18_ESM.pdf]

nature portfolio

Peer Review File

Cell autonomous regional differences in oligodendrocyte lineage development and responses to oncohistone H3.3 K27M

Corresponding Author: Professor Suzanne Baker

This file contains all reviewer reports in order by version, followed by all author rebuttals in order by version.

Version 0:

Reviewer comments:

Reviewer #1

(Remarks to the Author)
Diffuse midline glioma (DMG), H3K27-altered, is a near-universally fatal tumor arising in midline brain structures, most commonly in the pons during childhood. Multiple lines of evidence over the past 14 years suggest that H3K27M DMG arises from early precursor cells in the oligodendroglial lineage. However, the CNS region-specific susceptibility to K27M remains unclear.

An exciting manuscript by Budd et al. demonstrates how H3.3 K27M mutation disrupts region-specific oligodendrocyte development, contributing to DMG pathogenesis. Using a conditional knock-in mouse model, the authors show that H3 K27M selectively impairs oligodendrocyte differentiation in the brainstem, increasing OPC proliferation and disrupting intrinsic transcriptional programs. Single-cell RNA sequencing and in vitro differentiation assays reveal that wild type brainstem OPCs proliferate more slowly and differentiate later than telencephalon OPCs, but H3 K27M selectively accelerates brainstem OPC proliferation while restraining telencephalon OPC maturation. Mechanistically, H3 K27M-mediated epigenetic dysregulation upregulates key developmental and oncogenic pathways, including WNT, BMP, TGFB, and Notch, in the brainstem oligodendrocyte lineage. These findings highlight how H3 K27M disrupts regional OPC behavior, contributing to brainstem-selective gliomagenesis in DMG.

Overall, this study represents a major contribution to the field by demonstrating the region-specific and cell-type-specific effects of H3 K27M, addressing the mystery of why DMG arises exclusively in the midline. Addressing the following suggestions would further strengthen this excellent paper.

Major suggestions:
1. Figure 1: Assessment of myelination requires electron microscopy (as done in Figure 2). Decreased CNPase indicates a reduction in mature oligodendrocytes, but EM is required to claim a reduction in myelination, and whether this reflects thinner myelin sheaths, decreased myelinated axons, or both. EM is done is Figure 2, but language about the data in Figure 1 should reflect what the data shown, which illustrates a decrease in oligodendrocytes. Please adjust the language around the CNPase results to reflect an oligodendrocyte deficit, not a myelin deficit which is not shown until Figure 2.
2. What are “ectopic oligodendrocytes”? Oligodendrocytes are present throughout the brain, there is not region to which they are “ectopic”. I understand that there is an unusual density of mOLs in the caudal corpus callosum region, but “ectopic” is not quite the right wording and should be changed throughout.
3. Figure S8 – the Allen brain atlas is very useful, but ISH for MBP mRNA does not really show stages of developmental myelination. Better would be to immunostain a sagittal section of a mouse brain for PDGFRa (OPCs) and MBP protein (myelin), or use fluoromyelin to show myelin content.

4. The authors should clearly specify the exact location of the "dorsal telencephalon" in this paper. Does the term include the gray matter of the cerebral cortex? If so, the authors should perform additional analyses, including %CC1+Olig2+/Total Olig2+, Olig2+/mm², and %Olig2+Ki67+/Ki67+ in the cortex, given that Figure 3a shows a robust increase in EdU+ cells throughout the brain including cortex.

5. Overall, the small sample size in the animal experiments limits data interpretation, as several comparisons appear underpowered
a. In Figure 1e, the relative Cnp expression in the telencephalon shows a similar degree of change between the ctl and H3K27M groups across P7, P14, and P21. However, only the P7 data show statistical significance, likely due to underpowered sample size.
b. Figure 1g-1h has same issue and appears underpowered.
c. The authors should tone down the statement, “The proportion of proliferating cells that were OL-lineage (Olig2+;Ki67+/total Ki67+) increased in the VP but decreased in the cCC of K27M mice (Fig. 3e-f),” as the increase in Olig2+;Ki67+/total Ki67+ in the VP is not statistically significant. Alternatively, the authors could increase the sample size ('n') to strengthen their findings.
d. Same issue in this statement, “In accordance with these findings, Dcx, mRNA expression was increased in the telencephalon tissue (Fig 3h)”. The authors should tone down or increase the sample size (‘n’).

6. Figure 6h, the author should present the quantification of OL lineage morphology in 5-day differentiated data.

7. The authors used the same control data in Figures 5h and 6h without indicating that they are identical. To ensure clarity and transparency, the authors should either repeat the experiment to generate independent datasets or explicitly state that the same control data were used in both figures.

8. PMID: 28434841 should be cited as one of the early studies to point to an oligodendroglial lineage origin for DIPG/DMG, indicated by super-enhancer analyses highlighting oligodendroglial lineage identity genes. This paper is particularly important to discuss because the gene expression changes highlighted in this manuscript with introduction of H3K27M in OPCs were also found to be associated with super-enhancers in the PMID: 28434841 study of patient-derived H3K27M+ DIPG, including NOTCH, TGFb, integrin, Wnt, as well as synapse-related and axon pathfinding genes. It is important to make that connection.

Minor suggestions:
1. There is a literature on regional differences in OPCs – mostly focusing on neocortical vs white matter OPCs - that should be cited and discussed. See for example PMID: 30654924, PMID: 23995069
2. In Supplementary Figures 1c and 2a, the positions of H3K27me3 (green) and Flag (magenta) are swapped. The authors should correct the order to maintain consistency.
3. The authors should correct typo. For examples,
a. In abstract, the author should correct "Edu" to "EdU".
b. In the legend for Supplementary Figures 1c and 1d, "in WT-fl and K27M-fl mice at P14" should be corrected to "in WT-flag and K27M-flag mice at P14".
c. In the figure 5f legend, (Supplementary Figs. 2-3) should be corrected to (Supplementary Figs. 3-4).
4. Please increase and standardize the thickness of the scale bars.

5. The authors should standardize the format of Figure 4f and should align the graph of Figure 4f.

6. “incurable” is perhaps not the most accurate word. “lethal” or “typically fatal” is a fair description, but there have been rare complete responses to newer therapies for DMG…so “incurable” is inaccurate if there have in fact been – albeit rare – apparent cures.

7. Non-standard abbreviations are used throughout the paper to describe anatomical regions, which reads like alphabet soup. Please use actual anatomical names for clarity on the text, e.g. just call “BP” the basis pontis, and cCC the caudal corpus callosum. The abbreviations are distracting and don’t save many words. Similarly, “t-cOPC” or “b-cOPC” is unnecessarily complicated. Just use words instead of letters.

8. The observed upregulation in synapse-associated gene signatures in OPCs with the H3K27M mutation is very interesting and should be at least briefly discussed in the context of the known formation of pathogenic synapses between neurons and DMG cells (PMIDs: 31534222, 37914930, 39972132)

Reviewer #2

(Remarks to the Author)
Gliomas are a lethal brain tumor occurring in adults and children. While the distribution of the adult form is characteristically supra-tentorial, the pediatric form is characterized by a diffuse midline pontine location and the presence of an initiating somatic point mutation in the histone variant 3.3, where the lysine in position 27 is substituted by methionine. Previous work from several groups, had shown that this mutation is not sufficient for gliomagenesis. However, in combination with other mutations, it drives glioma growth in the brainstem (bs). The reasons responsible for the regionalizing effect of H3.3.K27M remain unclear and this manuscript addresses this interesting and puzzling question in the field.
As such, the work is of high significance in the field.
Using a conditional knock-in mouse line (expressing the mutant H3.3 K27M after deletion of a floxed STOP cassette from the endogenous H3.3 locus, using the human hGFAP promoter), the authors adopt a series of approaches, to characterize

the transcriptome and the developmental time course of oligodendrocyte progenitor differentiation in the brain stem and in the cortex. Most notably they generate tissue-of-origin specific cultures of OPCs, which they use to characterize proliferation, timing of differentiation and differential gene expression. The conclusion is that cultured bs-OPCs with wt H3.3:
- are less proliferative than their telencephalic counterpart (data in Figure 4), although the in vivo data are not statistically significant (Figure 3)
- tend to be slower to differentiate at day 1 and 2 in culture (Figure 5f), although this appears at odds with the well-established caudal to rostral timing of myelination.
They also report that the expression of the mutant H3.3.K27M blunts these differences and renders bs-OPC more similar to the telencephalic derived ones, in terms of proliferation, while blunting their differentiation potential, due to higher expression of genes related to WNT, NOTCH, BMP pathway.
Overall, there are a number of interesting concepts presented here. However, due to the choice of the experimental animal model and lack of a deep mechanistic understanding, the manuscript does not quite meet the expectation set by the introductory chapter. These issues, together with questionable data interpretation and not adequate consideration of previous reports on the PRC2 role in OPC, weaken the overall enthusiasm for the paper.

MAJOR CONCERNS

- It is well established that DMGs share the transcriptional signature of OPCs, as such it is surprising that the authors chose to use a knock-in model relying on the use of the human hGFAP promoter, which targets NPCs as well as astrocytes. While the authors mention that 91% of the OLIG2 cells expresses the mutant, the concern is the lack of consideration of several previous studies reported on the importance of:
- PRC2 activity in the transition from stem cells to OPC (https://doi.org/10.1371/journal.pone.0040399; https://doi.org/10.1634/stemcells.2008-0121)
- and in suppressing neuronal marker and synaptic protein expression in OPC (https://doi.org/10.1523/JNEUROSCI.2606-14.2015; https://doi.org/10.3389/fnins.2019.00829
- and in the oligo/astrocytic fate (references 47 and 48 cited, but not adequately taken into consideration for data analysis and discussion).

- The effects reported in mice could be consequent to a very different epigenetic landscape induced by H3.3.K27M in stem cells and or the possible interreference with genes mis-expressed in astrocytes and neural stem cells.

- The data on myelination are not convincing, as the effect on differentiation in mice is modest (Fig. 1), the myelin defects (Fig. 2) are questionable and they appear to be mostly driven by the poor quality of control tissue (likely due to poor fixation and processing)

- The data in figures 4 to 7 are more directly linked to the question initially posed by the manuscript and, while the authors convincingly show the existence of some differences, they fail to explain why or how H3.3K27M is responsible for them. Having ATAC seq, ChiP Seq data in wt and mutant OPCs from the two different brain regions could have helped in deciphering the puzzle.

- It is surprising that the authors do not attempt to carefully evaluate the wealth of resources on PRC2 targets in oligodendrocyte lineage cells available in the literature. This is a missed opportunity, especially since the pathways mentioned as upregulated in the H3.3. mutant OPCs, are in common with the ones reported un the EED and EZH2 conditional knockout mice. The dismissal of these and other important papers in oligodendrocyte lineage cells, is not warranted and a more careful evaluation of available datasets could have helped with the data interpretation, especially given the limitations of the animal model used in this study.

- While the authors ascribe some of the issues with differentiation to the upregulation of the WNT, NOTCH, BMP pathways, there is no attempt to rescue the phenotype with specific inhibitors.

Reviewer #3

(Remarks to the Author)
Budd et al. use a conditional H3.3K27M knock-in mouse model and cel culture systems to study the effects of the H3.3K27M mutation on the development of the oligodendriocyte cell lineage. The manuscript utilizes a battery of experimental techniques, ranging from imaging to single cel transcriptomics. The authors find that the K27M mutation causes an increase in proliferation and delays oligodendrocyte progenitor (OP) differentiation. This differentiation is region-specific and particularly affects the brainstem OPCs. The mutation does not appear to create a new cell state, and the authors conclude that it is the delayed development of brainstem OPCs that allows DM gliomagenesis.

I feel that this manuscript advances our knowledge of the early molecular events that lead to the development of DMGs. It merits publication and should enable further progress in follow up studies.

The two general weaknesses of the manuscript are: 1) Although a lot of work has been done, and the manuscript provides

some level of characterization of the molecular pathways that are dysregulated by the K27M mutation, the insights into why this happens in the brainstem as opposed to cortical or other brain regions is limited. 2) While the manuscript is well structured and thoughtfully argued, I found it to be heavy on the jargon, long, and difficult to understand. As a reader I would have appreciated a simpler language accessible to a wider audience.

As a reviewer, I find that this type of manuscript is difficult to properly review in two weeks. It is quite long with a great deal of supplementary data. Because of the range of techniques used, it needs to be reviewed by experts from several fields. Given the time constraints and my area of expertise, while I tried to follow most of the logic, I focused on the genomics. I am overall satisfied that the authors know what they are doing and have carried out appropriate analyses. I have only one minor comment:

Differential abundance testing, Figure S3. How was this done, what method was used? Intuitively, looking at figure S3E it seems like there are substantial differences at P4, for example in neuroblasts. Please provide information on the methodology and statistics, also justifying the choice of method. There are many "abundance testing" approaches and they do not always agree.

Version 1:

Reviewer comments:

Reviewer #1

(Remarks to the Author)
the authors have addressed all my comments. This is an exciting manuscript that represents an important contribution to the field.

My only minor comment relates to the wording of one sentence in the introduction: adaptive myelination continues throughout life and contributes to learning, memory and motor functions. I recommend rewording lines 88-89 from "…adaptive myelination in response to learning, memory, and motor function continuing into young adulthood" to more accurately read, "…adaptive myelination in response to neuronal activity continuing into adulthood and contributing to learning, memory, and motor functions."

Reviewer #3

(Remarks to the Author)
This is a re-review of a revised version of the original manuscript. I appreciate that the authors have taken the time to address my comments. I have no further concerns and recommend the manuscript for publication.

Open Access This Peer Review File is licensed under a Creative Commons Attribution 4.0 International License, which

permits use, sharing, adaptation, distribution and reproduction in any medium or format, as long as you give appropriate credit to the original author(s) and the source, provide a link to the Creative Commons license, and indicate if changes were made.
In cases where reviewers are anonymous, credit should be given to 'Anonymous Referee' and the source.
The images or other third party material in this Peer Review File are included in the article's Creative Commons license, unless indicated otherwise in a credit line to the material. If material is not included in the article's Creative Commons license and your intended use is not permitted by statutory regulation or exceeds the permitted use, you will need to obtain permission directly from the copyright holder.
To view a copy of this license, visit https://creativecommons.org/licenses/by/4.0/

Response to Reviewers

We thank the reviewers for their thoughtful and constructive feedback. We have strengthened the manuscript significantly by providing additional data to address reviewer comments.

Major changes in the revised manuscript include:

(1) Addition of new, immunofluorescence datasets with larger sample size to increase power to detect differences in updated Figures 1 and S3, and additional immunofluorescence and EdU detection in cleared whole brain images to increase power and enable more granular regional quantifications in updated Figure 2c-e, S4 and S5.

(2) Generation of new ChIP-seq and ATAC-seq data to investigate epigenetic mechanisms underlying K27M-driven transcriptional changes in a new Figure 6 and updated Figure 7.

(3) New immunofluorescence data in new Figure S10 replacing Allen Brain Atlas in situ hybridization images.

(4) Extensive revision to simplify language, remove jargon, and improve narrative flow.

(5) A complete revision of the Discussion to better frame our insights in relation to highly relevant literature to contextualize the significance of our work.

(6) Associated omics data has been uploaded to GEO (https://www.ncbi.nlm.nih.gov/geo/query/acc.cgi?acc=GSE310482) and is available for review with the following token:
adariwoerxuzvwd

Supportive Reviewer Comments:

We are pleased that all three reviewers noted the significance of our work that directly addresses an important unanswered question in brain tumor research connecting DMG pathogenesis and H3K27M effects in regional brain development:

Reviewer #1 noted "*Overall, this study represents a major contribution to the field by demonstrating the region-specific and cell-type-specific effects of H3 K27M, addressing the mystery of why DMG arises exclusively in the midline.*"

Reviewer #2 noted *"The reasons responsible for the regionalizing effect of H3.3.K27M remain unclear and this manuscript addresses this interesting and puzzling question in the field. As such, the work is of high significance in the field."*

Reviewer #3 noted *"I feel that this manuscript advances our knowledge of the early molecular events that lead to the development of DMGs. It merits publication and should enable further progress in follow up studies."*

We thank the reviewers for their helpful comments and suggestions. Our responses to each of the comments from their reviews are shown in red below.

REVIEWER COMMENTS

Reviewer #1:

Major suggestions:

***1**. Figure 1: Assessment of myelination requires electron microscopy (as done in Figure 2). Decreased CNPase indicates a reduction in mature oligodendrocytes, but EM is required to claim a reduction in myelination, and whether this reflects thinner myelin sheaths, decreased myelinated axons, or both. EM is done is Figure 2, but language about the data in Figure 1 should reflect what the data shown, which illustrates a decrease in oligodendrocytes. Please adjust the language around the CNPase results to reflect an oligodendrocyte deficit, not a myelin deficit which is not shown until Figure 2.*

We agree with the reviewer's comments and extensively modified Figure 1 with new data. We removed qRT-PCR and generated new datasets with increased sample size and rigorous anatomical matching. We evaluated immunofluorescence analysis of CNPase, a marker of mature oligodendrocytes, and myelin basic protein (MBP), a major component of myelin. We increased sample size to n=5-6 per group and adjusted our language around these results to reflect the regional reduction in CNPase+ or MBP+ staining (**Fig. 1c-d**) (Results page 6, lines 146-157). The electron microscopy data in original Fig. 2 had few replicates, was difficult to match regions, and had variable quality. Therefore, we elected to remove the EM data from original Fig. 2 and focus our interpretation on the protein expression patterns of these markers of mature oligodendrocytes and the MBP component of myelin.

2. *What are "ectopic oligodendrocytes"? Oligodendrocytes are present throughout the brain, there is not region to which they are "ectopic". I understand that there is an unusual density of mOLs in the caudal corpus callosum region, but "ectopic" is not quite the right wording and should be changed throughout.*

As the reviewer correctly states, oligodendrocytes are present throughout the brain and the term "ectopic" is not appropriate in this instance. We expanded the number of samples and immunofluorescence images for greater power and additional regional quantification. As we carefully reviewed the data with increased sample size and improved our methods to confirm a potential regional increase in mOL density, we found that increased Mbp signal and mature oligodendrocytes dorsal of the caudal corpus callosum was variable. Our prior observation may have related to the technical challenges of matching tissue for specific regions given the anatomical differences observed in control and K27M brains (**Supplementary Fig. 1e-i**). We therefore removed this statement to focus on robust and reproducible differences in staining for Mbp, CNPase, and the oligodendrocyte lineage markers (Olig2/CC1/Ki67) in specific brain regions analyzed in larger sample sizes (Revised **Fig. 1, Supplementary Fig. 3**), (Results page 6, lines 146-171).

3. *Figure S8 – the Allen brain atlas is very useful, but ISH for MBP mRNA does not really show stages of developmental myelination. Better would be to immunostain a sagittal section of a*

mouse brain for PDGFRa (OPCs) and MBP protein (myelin) or use fluoromyelin to show myelin content.

As suggested, we have replaced the original **Supplementary Fig. 8** with new immunofluorescence data for NG2-GFP (an OPC marker with more robust expression than PDGFRA) and Mbp in P0, P4, and P14 mouse brains (now **Supplementary Fig. 10**). These images show that Mbp is present in the brainstem at P0 while absent from the telencephalon. At P4, Mbp can be seen sparsely in the telencephalon, while at P14, Mbp is pervasive throughout the brain. NG2-EGFP positive cells can be seen throughout the brain at all timepoints, with CD31 staining also performed to distinguish from pericytes which also express NG2. Fluoromyelin gave a very bright signal when tested on control adult sections but was significantly weaker in the earlier developmental ages studied in this paper. The fluoromeyelin signal was consistent with the Mbp immunfluorescent patterns, but difficult to see in the low magnification images of whole brain.

The text has been edited (Results page 10, lines 270-271) to indicate this new data, though the original conclusions remain the same.

4. *The authors should clearly specify the exact location of the "dorsal telencephalon" in this paper. Does the term include the gray matter of the cerebral cortex? If so, the authors should perform additional analyses, including %CC1+Olig2+/Total Olig2+, Olig2+/mm², and %Olig2+Ki67+/Ki67+ in the cortex, given that Figure 3a shows a robust increase in EdU+ cells throughout the brain including cortex*.

We recognize the potential confusion. We have listed the broad anatomical regions composing the "dorsal telencephalon" in the Methods on page 21, lines 617-619:

"Briefly, brains were dissected from litters at P5-7 and the dorsal telencephalons (cortex, hippocampus, and corpus callosum) and brainstems were separated and pooled by region and genotype."

This does include the gray matter of the cerebral cortex. We incorporated quantifications of a new dataset of Olig2/CC1/Ki67 immunofluorescence (n=6 for each genotype) in **Figs. 1e-i and Supplementary Fig. 3** including the specific analyses requested by Reviewer 1. These new data provide additional insights into the oligodendrocyte lineage composition, proportion of proliferating cells, and cellular density in each region, including the cerebral cortex. A new **Supplementary Fig. 3a** shows a representative example of the areas quantified for additional clarity.

As summarized in the Results on page 6, lines 158-171:

"Based on region-specific reduction in CNPase and MBP, we hypothesized that K27M may alter oligodendrocyte maturation. Thus, we co-immunostained for Olig2 (a pan-OL lineage marker), CC1 (a mOL marker), and Ki67 (a proliferation marker) at P14 (**Fig. 1e**; **Supplementary Fig. 3**; **Supplementary Table 1**). The total proportion of oligodendrocyte lineage cells (Olig2+) was not significantly changed by K27M. However, we found a significant K27M-dependent reduction in the proportion of CC1+ mOLs and a corresponding increase in the proportion of proliferating

OPCs (Olig2+;CC1-;Ki67+) as compared to all Olig2+ cells across both brainstem and telencephalon regions (**Fig. 1f-g**). The proportion of OPCs proliferating increased in the basis pontis, pontine tegmentum, and the rostral cortex (**Fig. 1h**), but the proportion of all proliferating cells that were OPCs only increased significantly in the basis pontis (**Supplementary Fig. 3c**). Notably, K27M increased total cell density in all regions assessed (**Supplementary Fig. 3d**) but only significantly increased Olig2+ cell density in the pontine tegmentum and rostral cortex (**Supplemental Fig. 3e**)."

5. *Overall, the small sample size in the animal experiments limits data interpretation, as several comparisons appear underpowered*
a*. In Figure 1e, the relative Cnp expression in the telencephalon shows a similar degree of change between the ctl and H3K27M groups across P7, P14, and P21. However, only the P7 data show statistical significance, likely due to underpowered sample size.*

b*. Figure 1g-1h has same issue and appears underpowered.*

We addressed the reviewer's concern by greatly expanding these studies. As mentioned in the response to Reviewer major suggestion #1, **Original Fig. 1e, g-h** has been replaced with quantifications of a new, larger CNPase/MBP tissue section IF dataset (**Fig. 1c-d**) and a new, larger Olig2/CC1/Ki67 tissue section IF dataset with cortical regions also quantified (**Fig. 1e-g; Supplementary Fig. 3**), (Results, page 6-7, lines 146-177). We appreciate the reviewer's suggestions and are please to include these more robust data sets.

c. *The authors should tone down the statement, "The proportion of proliferating cells that were OL-lineage (Olig2+;Ki67+/total Ki67+) increased in the VP but decreased in the cCC of K27M mice (Fig. 3e-f)," as the increase in Olig2+;Ki67+/total Ki67+ in the VP is not statistically significant. Alternatively, the authors could increase the sample size ('n') to strengthen their findings.*

We replaced the **Original Fig. 3e-f** with the new, larger Olig2/CC1/Ki67 tissue section IF dataset with cortical regions also quantified in **Fig. 1e-g; Supplementary Fig. 3.** We strengthened our findings with increased sample size and re-evaluated our interpretations as discussed in the response to Reviewer comment #4 above.

We also generated new, more granular cleared whole brain imaging datasets with EdU and Olig2 staining (**Fig. 2c-e; Supplementary Fig. 4**). This comprehensive brain-wide view showed a broad increase in proliferating cells throughout the brain, with only modest, selective increase in total Olig2-expressing cells (Results, page 7, lines 181-202).

d*. Same issue in this statement, "In accordance with these findings, Dcx, mRNA expression was increased in the telencephalon tissue (Fig 3h)". The authors should tone down or increase the sample size ('n').*

We agree with the reviewer and have removed **Original Fig. 3h** in favor of the more robust IF data for Dcx (**Fig. 2g-h**) (Results, page 8, lines 203-208).

6. *Figure 6h, the author should present the quantification of OL lineage morphology in 5-day differentiated data.*

See response to Reviewer comment #7 below.

7. *The authors used the same control data in Figures 5h and 6h without indicating that they are identical. To ensure clarity and transparency, the authors should either repeat the experiment to generate independent datasets or explicitly state that the same control data were used in both figures.*

We thank the reviewer for this comment. The same control data was used in both figures and was originally presented separately for clarity to narrate differences between brain regions in normal controls before considering effects of the K27M mutation. Due to technical difficulties, the morphology studies had low sample number and scoring morphology is much less objective than the assessment of RNA and protein expression. For both of these reasons, we removed the morphology experiment from the Figures and Results (**original Figs. 5h; 6h**).

For a more objective and sufficiently powered approach to demonstrate shifts in differentiation dynamics, we re-analyzed our deconvolution results from bulk RNA-seq of the OPC cultures to derive a differentiation index for each culture. This more rigorous statistic has replaced the original subjective categorical scoring for pair-wise testing between genotypes and tissue origin (**Figs. 4f; 5g**) (Results, page 13, lines 366-375 and pages 14-15, lines 415-423).

8. *PMID: 28434841 should be cited as one of the early studies to point to an oligodendroglial lineage origin for DIPG/DMG, indicated by super-enhancer analyses highlighting oligodendroglial lineage identity genes. This paper is particularly important to discuss because the gene expression changes highlighted in this manuscript with introduction of H3K27M in OPCs were also found to be associated with super-enhancers in the PMID: 28434841 study of patient-derived H3K27M+ DIPG, including NOTCH, TGFb, integrin, Wnt, as well as synapse-related and axon pathfinding genes. It is important to make that connection.*

We agree that this is an important reference and have included it (ref. 20) in the Introduction and Discussion. Specifically, in the Introduction on page 3, lines 72-74, we state:

"the temporal incidence of DMG and the super-enhancer landscape of DMG support oligodendrocyte progenitor cells (OPC) as likely DMG candidate cells of origin[16-20]."

In the Discussion on page 20, lines 572-576, we state:

"Bmp, Wnt, and Notch regulate glial lineage commitment, OPC proliferation, and myelination, and are linked to oncogenic behavior and invasion in DMG and other gliomas[18,22,53,54]. Genes within these pathways are also associated with super enhancers in patient-derived DMG cell

lines[20], highlighting the disease relevance of the K27M-induced signatures in the brainstem oligodendrocytic lineage."

Minor suggestions:

***1**. There is a literature on regional differences in OPCs – mostly focusing on neocortical vs white matter OPCs - that should be cited and discussed. See for example PMID: 30654924, PMID: 23995069*

We agree with the reviewer that intrinsic regional effects on OPC development are an important aspect of our study and have included these publications (refs. 27, 28) in our revised Introduction on page 4, lines 92-98:

"Transplantation studies have demonstrated cell-extrinsic and -intrinsic regional effects on OPC maturation comparing adult neocortical gray matter and underlying white matter. The white matter was a more conducive environment for OPC differentiation and produced OPCs that differentiated more efficiently than gray matter OPCs regardless of the region of transplantation[27]. The heterogeneous electrophysiological properties of OPCs also influence their functional states and show both age-dependent and regional variation[28]."

And in the Discussion on page 18, lines 523-534:

"Recent single-nucleus RNA-seq analyses of adult human brain inferred lower OPC proliferation, slower oligodendrocyte differentiation, and reduced turnover of mature oligodendrocytes in brainstem relative to telencephalon[50]. They hypothesized more frequent replenishment of mature oligodendrocytes in the telencephalon compared to brainstem, aligning with our *in vitro* findings that wild-type brainstem OPCs proliferate and transition more slowly into committed oligodendrocyte progenitors than telencephalon OPCs which appear "primed" for differentiation. *In vivo*, myelination and the rise of mature oligodendrocytes occur earlier in the brainstem than telencephalon[26,51], and OPCs exhibit regional variation in differentiation capacity driven by local niche context (white versus gray matter) and age[27,28]. Our data suggest both extrinsic cues and intrinsic OPC state, which K27M specifically perturbs, orchestrate these developmental timing differences."

***2**. In Supplementary Figures 1c and 2a, the positions of H3K27me3 (green) and Flag (magenta) are swapped. The authors should correct the order to maintain consistency.*

These figures have been adjusted to maintain consistent ordering.

***3**. The authors should correct typos. For examples,*
a. In abstract, the author should correct "Edu" to "EdU".
b. In the legend for Supplementary Figures 1c and 1d, "in WT-fl and K27M-fl mice at P14" should be corrected to "in WT-flag and K27M-flag mice at P14".
c. In the figure 5f legend, (Supplementary Figs. 2-3) should be corrected to (Supplementary Figs. 3-4)

These mistakes have all been corrected.

4. *Please increase and standardize the thickness of the scale bars*.

The thickness of the scalebars has been increased and made consistent within images.

5. *The authors should standardize the format of Figure 4f and should align the graph of Figure 4f.*

This figure has been adjusted for better alignment.

6. *"incurable" is perhaps not the most accurate word. "lethal" or "typically fatal" is a fair description, but there have been rare complete responses to newer therapies for DMG…so "incurable" is inaccurate if there have in fact been – albeit rare – apparent cures.*

"incurable" has been changed to "lethal" throughout the text.

7. *Non-standard abbreviations are used throughout the paper to describe anatomical regions, which reads like alphabet soup. Please use actual anatomical names for clarity on the text, e.g. just call "BP" the basis pontis, and cCC the caudal corpus callosum. The abbreviations are distracting and don't save many words. Similarly, "t-cOPC" or "b-cOPC" is unnecessarily complicated. Just use words instead of letters.*

Text has been de-"soupified", by replacing jargon and abbreviations with clearer language as recommended.

8. *The observed upregulation in synapse-associated gene signatures in OPCs with the H3K27M mutation is very interesting and should be at least briefly discussed in the context of the known formation of pathogenic synapses between neurons and DMG cells (PMIDs: 31534222, 37914930, 39972132)*

We agree with the reviewer's important recommendation and have added a paragraph to the discussion on page 20, lines 584-596, to emphasize its significance and tie in with the suggested publications (refs. 55-57):

"DMG cells form neuron-glioma synapses, integrate into neural circuits, and exploit activity-dependent signaling to drive proliferation and invasion[55-57], and genes associated with synapses and axon pathfinding are also associated with super-enhancers in DMG cells[20]. The observed cell intrinsic K27M-dependent up-regulation of axon guidance, synapse, and neurite outgrowth programs, especially in brainstem-derived OPCs, provides a plausible developmental precursor to these malignant neuronal interactions. These K27M-driven changes align with prior observations of H3K27me3 deposition and PRC2 binding at neuronal genes during OPC differentiation[58,59]. Even before acquisition of additional driver mutations, K27M OPCs in the

brainstem may be biased towards heightened responsiveness to neuronal activity, trophic cues, and axonal scaffolding. The interplay between intrinsic regional OPC characteristics, K27M, and external cues contributing to pathogenesis warrants further investigation."

Reviewer #2:

We appreciate the Reviewer's recognition of the significance of our study:

"The reasons responsible for the regionalizing effect of H3.3.K27M remain unclear and this manuscript addresses this interesting and puzzling question in the field.
As such, the work is of high significance in the field."

We will first address the Reviewer's comments in their summary overview about differences between *in vivo* and *in vitro* results in our manuscript.

"Most notably they generate tissue-of-origin specific cultures of OPCs, which they use to characterize proliferation, timing of differentiation and differential gene expression. The conclusion is that cultured bs-OPCs with wt H3.3:

- are less proliferative than their telencephalic counterpart (data in Figure 4), although the in vivo data are not statistically significant (Figure 3)

Results from *in vitro* and *in vivo* studies are not necessarily expected to be identical. As stated in the manuscript, *in vitro* conditions allow us to examine cell autonomous properties while *in vivo* conditions incorporate both cell intrinsic properties and cell extrinsic cues associated with interactions with other cell types and the microenvironment that could certainly influence *in vivo* proliferation. Additionally, because of the small size of the developing brain structures, it was necessary to establish cultures from pooled littermates using entire brainstem and telencephalon from developing pups to generate sufficient material for *in vitro* experiments.

Our prior *in vivo* analyses of OPC proliferation (prior **Fig. 3**) specifically evaluated OPC proliferation in the ventral pons and the caudal corpus callosum. As discussed in the response to Reviewer 1, point #4, we generated a new data set with larger sample size comparing proliferating and mature oligodendrocytes in control vs K27M brain using triple labeling of Olig2 (all cells in the oligo lineage), CC1 (mature oligodendrocytes) and Ki67 (n=5-6 for each genotype) (Results, pages 6-7, lines 146-177). We expanded the number of regions quantified for oligodendrocyte lineage composition, evaluating additional regions within the pons and telencephalon (new **Fig. 1** and **Supplementary Fig. 3**). We find regional differences within the pons and within the telencephalon in the proportion of proliferating OPCs in control brains (**Fig. 1h**). Results from cultures derived from pooled regions would not be expected to perfectly match *in vivo* findings showing differences in subregions. Given the contribution of cell intrinsic and cell extrinsic effects on proliferation *in vivo,* and the *in vivo* regional variations in OPC proliferation rates that cannot be precisely deconvoluted in pooled OPC cultures, we do not believe that *in vitro* and *in vivo* differences are problematic.

- tend to be slower to differentiate at day 1 and 2 in culture (Figure 5f), although this appears at odds with the well-established caudal to rostral timing of myelination.

The slower differentiation rate of wild-type brainstem OPCs is not at odds with the caudal to rostral timing of myelination. We address this in the Discussion on page 18, line 523-534:

"Recent single-nucleus RNA-seq analyses of adult human brain inferred lower OPC proliferation, slower oligodendrocyte differentiation, and reduced turnover of mature oligodendrocytes in brainstem relative to telencephalon[50]. They hypothesized more frequent replenishment of mature oligodendrocytes in the telencephalon compared to brainstem, aligning with our *in vitro* findings that wild-type brainstem OPCs proliferate and transition more slowly into committed oligodendrocyte progenitors than telencephalon OPCs which appear "primed" for differentiation. *In vivo*, myelination and the rise of mature oligodendrocytes occur earlier in the brainstem than telencephalon[26,51], and OPCs exhibit regional variation in differentiation capacity driven by local niche context (white versus gray matter) and age[27,28]. Our data suggest both extrinsic cues and intrinsic OPC state, which K27M specifically perturbs, orchestrate these developmental timing differences."

Additional overall comments are addressed in the Major Concern points below as noted here:

"Overall, there are a number of interesting concepts presented here. However, due to the choice of the experimental animal model (addressed below in Major Concern 1) *and lack of a deep mechanistic understanding* (addressed below in Major Concern 5), *the manuscript does not quite meet the expectation set by the introductory chapter. These issues, together with questionable data interpretation* (addressed below in Major Concern 4) *and not adequate consideration of previous reports on the PRC2 role in OPC* (addressed below in Major Concern 2), *weaken the overall enthusiasm for the paper."*

We thank the reviewer for their feedback and suggestions. We have numbered their listed concerns to aid reference to specific points elsewhere in our response.

MAJOR CONCERNS

Relating to the Reviewer's comment above regarding choice of experimental animal model:

1. "*It is well established that DMGs share the transcriptional signature of OPCs, as such it is surprising that the authors chose to use a knock-in model relying on the use of the human hGFAP promoter, which targets NPCs as well as astrocytes.*"

Of relevance to our choice of hGFAP-Cre, we first established the Cre-inducible H3.3 K27M knock-in mouse to model H3.3 K27M oncogenic activity in cooperation with p53 loss and PDGFRA activation in neural stem/progenitor cells. Inducing all three of these alterations in neural stem/progenitor cells resulted in DMG selectively arising in the brainstem, with highly significant recapitulation of transcriptional and epigenetic signatures and histopathological features of human DMG (Larson et al, Cancer Cell 2019). Thus, we have robustly and directly demonstrated that inducing H3K27M mutation in neural stem/progenitor cells is highly relevant to understanding its contribution to DMG pathogenesis.

Although the transcriptional signature in the majority of DMG tumor cells most resembles OPCs, the cell of origin in which the K27M mutation first occurs, and that gives rise to human DMG is not necessarily restricted to OPCs. In fact, several model systems introduced H3K27M along with loss of function of TP53 and activation of PDGFRA in nestin-expressing neural stem/progenitor cells and generated DMG models that strongly recapitulate the mutational, transcriptional, and epigenetic signatures as well as histopathological features of H3K27M-mutant DMG. These include models using human ES cell-derived neural stem-progenitor cells (Funato et al, Science 2014), purified mouse neural stem cells (Mohammad et al, Nature Medicine 2017), in utero electroporated neural progenitor cells (Pathania et al, Cancer Cell 2017, Patel et al, Neuro Oncol 2020), and virally transduced mouse neural progenitor cells (Cordero et al, Mol Cancer Res 2017).

Engineering neural stem/progenitor cells to express H3K27M typically results in expression in all subsequent progeny. Notably, there is some evidence that NSCs have a greater propensity to generate K27M-mutant DMG compared to OPCs. Haag et al, Cancer Cell 2021 compared NSCs and OPCs induced from human iPSCs. They showed that induction of H3.3 K27M and TP53 loss of function in NSCs, but not in OPCs gave rise to DMG xenografts when engrafted in mice. Consistent with this conclusion, Tomita et al, Glia 2022 used retroviral transduction to directly compare DMG induced by combined H3.3 K27M, p53 deletion and PDGF overexpression in different cell types and found that H3.3K27M is less oncogenic in Olig2+ cells than in Nestin+ cells, only accelerating tumor cell proliferation and decreasing latency when introduced in Nestin+ cells.

All the above model systems generated Olig2+ tumor cells that demonstrated transcriptional signatures of the resulting tumors that strongly recapitulated signatures found in human DMG. It is possible that introducing the K27M mutation in neural stem/progenitor cells may confer a greater oncogenic activity in progeny OPC-like cells than if the mutation occurs directly in OPCs. This is an important concept in cancer, particularly those tumors arising in the context of development, and is nicely discussed, in Filbin and Monje, Nature Medicine 2019 (PMID: 30842674) considering the "cell of mutation" as potentially a neural stem/progenitor cell, that may then give rise to OPC progeny cells that serve as the cell of origin for a subsequent DMG. We include the "cell of mutation" concept on page 5, lines 118-120. While the precise timing of K27M mutation is not proven, especially considering variation across DMGs, abundant evidence from multiple models generated by different investigators supports the strong relevance of introducing K27M in neural stem/progenitor cells to generate DMG recapitulating the OPC-like phenotype found in the human disease. Therefore, we feel that our choice of model system is well-justified.

2. (Also relates to the Reviewer's comment in their summary overview "*not adequate consideration of previous reports on the PRC2 role in OPC*")

While the authors mention that 91% of the OLIG2 cells expresses the mutant, the concern is the lack of consideration of several previous studies reported on the importance of:

A. *PRC2 activity in the transition from stem cells to OPC (https://doi.org/10.1371/journal.pone.0040399;https://doi.org/10.1634/stemcells.2008-0121)*
B. *and in suppressing neuronal marker and synaptic protein expression in OPC (https://doi.org/10.1523/JNEUROSCI.2606-14.2015; https://doi.org/10.3389/fnins.2019.00829*

These studies provide interesting insights into some of the transcriptional changes we observe, so we have included them in the Discussion (see response to major concern #3 below).

C. *and in the oligo/astrocytic fate (references 47 and 48 cited, but not adequately taken into consideration for data analysis and discussion).*

We share the reviewer's interest in the impacts of K27M on other cell lineages and progenitors, which was part of the motivation for selecting a model that targets development from neural stem cells through both astrocytic and oligodendroglial lineages.

We found no evidence that H3K27M expression in OPCs induced the astrocytic fate switch observed in PRC2-null OPCs as seen in refs. 32-33. We found no evidence of significant increase in Gfap-expressing cells by immunofluorescence or RNA expression of astrocytic markers Gfap, Aldh1l1, or Slc1a3 (**Supplementary Fig. 7**), (discussed on page 8, lines 212-217) or in single cell sequencing data (**Supplementary Figs. 8 and 9**). Indeed, as reported in the Results section, page 9, lines 247-251 on single cell analysis:

"Cells in the glial lineages (astrocytes and oligodendrocytes) were most impacted, retaining signatures of immature OPCs and reduced differentiated cell state signatures (**Supplementary Table 4, Supplementary Fig. 9a**). K27M also drives intrinsic neuronal expression programs in both the astrocyte and oligodendrocyte lineages."

Therefore, we focused on the oligodendrocyte lineage due to the overriding OPC signatures in DMG cells.

We further clarified the distinction between K27M mutation and PRC2 loss of function in the Discussion on page 19, lines 542-548:

"Importantly, the K27M phenotype we observe is distinct from complete PRC2 loss induced by *Eed* deletion, which produces an OPC-to-astrocyte fate switch and broad lineage reprogramming in both cortex and brainstem[32,33]. Through single-cell sequencing as well as focused IF analysis of marker gene expression, we found no evidence of new cell types, cell fate shifts, or increased numbers of GFAP+ astrocytes, likely related to the relative extent of K27M-mediated disruption in PRC2-mediated methylation, which is dramatic but not equivalent to global PRC2 complete loss. "

Please also see response to major concern #6 below.

3. *The effects reported in mice could be consequent to a very different epigenetic landscape*

induced by H3.3.K27M in stem cells and or the possible interreference with genes mis-expressed in astrocytes and neural stem cells.

We agree that extrinsic cues affecting oligodendrocyte differentiation *in vivo* are likely to be important, but our synchronized *in vitro* differentiation of OPC cultures display intrinsic K27M-driven differences in differentiation dynamics (**Figs. 3-5**). This indicates the effects we report in mice are likely not due solely to interactions with other cell lineages or progenitors. We agree the interplay of oligodendrocytes with other cell lineages, particularly in the context of gliomagenesis, is an area worthy of additional study and have added the following section to the Discussion, page 20, lines 584-596 to emphasize this:

"DMG cells form neuron-glioma synapses, integrate into neural circuits, and exploit activity-dependent signaling to drive proliferation and invasion[55-57], and genes associated with synapses and axon pathfinding are also associated with super-enhancers in DMG cells[20]. The observed cell intrinsic K27M-dependent up-regulation of axon guidance, synapse, and neurite outgrowth programs, especially in brainstem-derived OPCs, provides a plausible developmental precursor to these malignant neuronal interactions. These K27M-driven changes align with prior observations of H3K27me3 deposition and PRC2 binding at neuronal genes during OPC differentiation[58,59]. Even before acquisition of additional driver mutations, K27M OPCs in the brainstem may be biased towards heightened responsiveness to neuronal activity, trophic cues, and axonal scaffolding. The interplay between intrinsic regional OPC characteristics, K27M, and external cues contributing to pathogenesis warrants further investigation.

4. *The data on myelination are not convincing, as the effect on differentiation in mice is modest (Fig. 1), the myelin defects (Fig. 2) are questionable and they appear to be mostly driven by the poor quality of control tissue (likely due to poor fixation and processing.)*

The main aim of our study is to evaluate how the K27M mutation impacts oligodendrocyte maturation, which may be relevant to DMG pathogenesis. As addressed in Major Suggestion 1 from Reviewer 1, due to variable quality, we removed the electron microscopy data from prior Fig. 2 and we generated much more robust larger sample sized immunofluorescence datasets with CNPase and MBP (**Fig. 1**), triple labeling for Olig2, CC1 and Ki67 (**Fig. 1 and Supplementary Fig. 3**) (Results, pages 6-7, lines 146-177), and tissue clearing data for Edu labeling and Olig2 labeling (**Fig. 2 and Supplementary Fig. 4**) (Results, page 7, lines 181-197) demonstrating that the effect of K27M on differentiation in mice is significant and variable by brain region (n=5-6 per group).

5. *The data in figures 4 to 7 are more directly linked to the question initially posed by the manuscript and, while the authors convincingly show the existence of some differences, they fail to explain why or how H3.3K27M is responsible for them. Having ATAC seq, ChiP Seq data in wt and mutant OPCs from the two different brain regions could have helped in deciphering the puzzle.*

We agree that this is an important consideration and have generated significant new data to address this point. These data (**new Fig. 6 and revised Fig. 7**) reveal that:

a. K27M has a preferential transcriptional impact on bivalent genes (**Fig. 6a**) (Results, page 15, lines 426-438).

b. Chromatin state at promoters is most variable between brainstem and telencephalon OPCs at bivalent (H3K4me3+/H3K27me3+) and repressed (H3K4me-/H3K27me3+) promoters (**Fig. 6b**) (Results, page 15, lines 438-442).

c. Regional patterning transcription factors maintain H3K27me3 despite global depletion, preserving regional identity (**Fig. 6e**) (Results, page 15-16, lines 447-450).

d. Developmental signaling genes (BMP, WNT, Notch, TGFβ, integrin) are bivalently marked in brainstem and telencephalon OPCs but are specifically upregulated by K27M in brainstem OPCs (**Fig. 7**) (Results, page 17, lines 479-497).

These findings are presented in the Results, pages 15-16, lines 426-453:

"Selective H3K27me3 loss at bivalent promoters drives H3.3 K27M-mediated transcriptional changes

We previously compared isogenic DMG models with and without K27M mutation and showed that K27M causes widespread loss of H3K27me3 at gene promoters, but a preferential transcriptional impact in mouse and human DMGs on genes with bivalent promoters marked by both H3K4me3 and H3K27me3 in wild-type controls[10,24]. To determine if K27M impacts gene expression through the same mechanism in OPCs in the developing brain, we performed ChIP-seq for H3K4me3 and H3K27me3 along with ATAC-seq in control and K27M brainstem- and telencephalon-derived OPC cultures to assess promoter state. We observed a significantly outsized proportion of bivalently marked genes when comparing promoter state proportions of K27M up- or down-regulated genes to all genes (**Fig. 6a**). Overlap analyses revealed that most genes with unmarked (H3K4me3-; H3K27me3-) and active (H3K4me3+; H3K27me3-) promoters were shared between control OPC cultures from each region while genes with repressed (H3K4me3-; H3K27me3+) or bivalent (H3K4me3+; H3K27me3+) promoters were shared less frequently between the regions (**Fig. 6b**).

The chromatin landscape at the transcription start sites of bivalent promoters demonstrates H3K27me3 loss in K27M up-regulated genes and H3K27me3 retention at K27M down-regulated genes in both brainstem- (**Fig. 6c**) and telencephalon-derived (**Fig. 6d**) OPC cultures. Both H3K4me3 and ATAC-seq were largely unaffected by H3 status. Importantly, many of the genes most strongly down-regulated by K27M in either tissue are key developmental and patterning transcription factors that retain or increase H3K27me3 at their promoters (e.g. *Irx* family members in telencephalon and *Lhx* members in brainstem; **Fig. 6e**). Together, this indicates that K27M drives transcriptional reprogramming in OPCs through depletion of H3K27me3 from bivalently marked promoters while maintaining H3K27me3-mediated repression at key developmental and tissue-identity genes."

Further, integration of the epigenetic changes with expression in developmental signaling pathways is discussed in Results, page 17, lines 479-497:

"However, developmental signaling pathways, such as Bmp, Notch, Wnt, Tgfb, and integrin, were broadly and strikingly up-regulated by K27M preferentially in brainstem-derived OPC cultures (**Fig. 7a-b**). At later stages of differentiation, developmental signaling pathway expression is notably increased yet minimally impacted by K27M in telencephalon-derived OPC cultures compared to brainstem-derived OPC cultures (**Fig. 7a**), again indicating that K27M ablates typical region-specific expression programs.

We next assessed bivalency status of these genes, finding 49% (39/80) and 68% (54/80) of the genes have bivalent promoters in control brainstem- or telencephalon-derived OPC cultures, respectively (**Fig. 7b**). To determine if these signaling pathway genes are impacted by K27M in DMG, we assessed their expression and promoter bivalency status in xenograft and cell line H3 K27M DMG models from previous studies where K27M was either knocked down via shRNA[24] or knocked out via CRISPR-Cas9[23], respectively (**Fig. 7b**). In total, we found that 73% (58/80) of the genes showed K27M-dependent up-regulation and/or promoter bivalency in at least one of the human DMG models (**Fig. 7b**; **Supplementary Table 12**).

K27M-mediated H3K27me3 loss activates bivalent genes with dual roles in neurodevelopment and tumorigenesis preferentially in the brain stem, providing a compelling epigenetic mechanism for region-specific vulnerability to DMG."

e. We expanded the Discussion to emphasize these new mechanistic insights, better contextualize them with existing DMG literature, and highlight this outstanding question. On pages 19-20, lines 549-596:

"By integrating region-resolved *in vivo* imaging, single-cell and bulk transcriptomics, and epigenomic profiling with an *in vitro* OPC culture system, we mechanistically link regional developmental programs, bivalent chromatin, and the midline-restricted oncogenic potential of H3.3 K27M. At the chromatin level, K27M in DMG and neural stem cell models disproportionately affects genes whose promoters are bivalently marked by H3K4me3 and H3K27me3[10,14,24]. Consistent with these findings, here we show that K27M up-regulated genes in OPCs from both brainstem and telencephalon are enriched for bivalent promoters that lose H3K27me3 while retaining H3K4me3 and open chromatin at their transcription start sites. K27M simultaneously derepresses a subset of developmental programs while preserving repression of key patterning and identity transcription factors. The distribution of bivalent promoters is regionally patterned, suggesting that oncohistone-mediated re-programming will intersect distinct developmental networks in each region.

A central finding of our study is that K27M preferentially and robustly up-regulates multiple developmental signaling pathways in brainstem-derived OPCs. Bmp, Wnt, Notch, TGFβ, and integrin pathways play complex and varied roles in all stages of neurogenesis from stem cells through differentiation[52], and are upregulated by K27M in brainstem-derived OPCs, but intrinsically expressed throughout differentiation and not up-regulated by K27M in telencephalon-derived OPCs reflecting context-dependent regional differences in developmental

regulation. These regulators of signal transduction are collectively over-represented among K27M up-regulated, bivalently marked genes, and a majority of them show K27M-dependent up-regulation and/or promoter bivalency in human DMG models where K27M is knocked out or knocked down[24,43]. Bmp, Wnt, and Notch regulate glial lineage commitment, OPC proliferation, and myelination, and are linked to oncogenic behavior and invasion in DMG and other gliomas[18,22,53,54]. Genes within these pathways are also associated with super enhancers in patient-derived DMG cell lines[20], highlighting the disease relevance of the K27M-induced signatures in the brainstem oligodendrocytic lineage.

Our data support a model in which K27M lifts PRC2 repression at a brainstem-biased set of bivalent developmental genes, igniting aberrant, self-reinforcing signaling that sustains an immature, proliferative OPC-like state and may affect motility, adhesion, and axonal or synaptic interactions. Additional studies are necessary to better understand the regionally selective impacts of K27M on these pathways, how they relate to normal oligodendroglial development, and how they can be effectively targeted for therapeutic benefit in DMG.

DMG cells form neuron-glioma synapses, integrate into neural circuits, and exploit activity-dependent signaling to drive proliferation and invasion[55-57], and genes associated with synapses and axon pathfinding are also associated with super-enhancers in DMG cells[20]. The observed cell intrinsic K27M-dependent up-regulation of axon guidance, synapse, and neurite outgrowth programs, especially in brainstem-derived OPCs, provides a plausible developmental precursor to these malignant neuronal interactions. These K27M-driven changes align with prior observations of H3K27me3 deposition and PRC2 binding at neuronal genes during OPC differentiation[58,59]. Even before acquisition of additional driver mutations, K27M OPCs in the brainstem may be biased towards heightened responsiveness to neuronal activity, trophic cues, and axonal scaffolding. The interplay between intrinsic regional OPC characteristics, K27M, and external cues contributing to pathogenesis warrants further investigation."

6. *"It is surprising that the authors do not attempt to carefully evaluate the wealth of resources on PRC2 targets in oligodendrocyte lineage cells available in the literature. This is a missed opportunity, especially since the pathways mentioned as upregulated in the H3.3. mutant OPCs, are in common with the ones reported in the EED and EZH2 conditional knockout mice. The dismissal of these and other important papers in oligodendrocyte lineage cells, is not warranted and a more careful evaluation of available datasets could have helped with the data interpretation, especially given the limitations of the animal model used in this study."*

We originally included comparisons with gene sets arising from EED models in our cultured OPC RNA-seq GSEA analyses. These gene sets were collected or derived from two studies (refs. 28-29) with conditional EED deletion in isolated mouse OPCs/iOLs and PRC2 targets derived from Eed/Suz12 ChIP-seq of P3 spinal cord (**Supplementary Table 9**).

As noted by the reviewer, genes upregulated by EED knockout are also highly enriched in H3 K27M versus H3 WT OPCs throughout differentiation, as are PRC2 targets. In response to this recommendation, we have added these pathways to the GSEA results shown in **Fig. 3h** and added the following to the Results, page 11, lines 312-322:

“We performed pre-ranked gene set enrichment analysis (GSEA) on differential expression analyses of the K27M and control OPC cultures at each timepoint split by origin tissue (**Supplementary Table 8**) using K27M up-regulated and PRC2 target gene sets taken or derived from published datasets of primary patient tumors, patient-derived orthotopic xenografts, mouse neural stem cell models, mouse OPCs, and mouse model tumors (**Fig. 3h**; **Supplementary Table 9**)[10,21,24,43]. These gene sets showed significant positive enrichment (p.adj < 0.05) in nearly all K27M versus control comparisons across timepoints and tissue origin. As expected, K27M up-regulated genes are positively enriched for PRC2 targets and genes up-regulated in PRC2-null OPCs and immature oligodendrocytes across timepoints and tissue origin.”

7. *While the authors ascribe some of the issues with differentiation to the upregulation of the WNT, NOTCH, BMP pathways, there is no attempt to rescue the phenotype with specific inhibitors.*

We agree that inhibition of these pathways would be of interest in future studies assessing potential vulnerabilities of K27M mutant DMG. However, it is noteworthy that there are simultaneous expression changes in subsets of the components of multiple developmental pathways. Each of the pathways has complex context-dependent roles in cell state regulation in different glial lineages, and there is extensive cross-talk among the pathways. The expression changes induced by K27M are not simple binary on/off alterations. As this study focuses on the K27M effects on oligodendrocyte lineage differentiation and development, we feel that the well-characterized and profound impact induced by very strong manipulation of these crucial developmental pathways in oligodendrocyte development and differentiation may mask K27M phenotypes in this setting. Identifying the relevant selective inhibitors and optimal concentrations to mimic reversal of the physiologically relevant changes in expression levels associated with K27M would be a significant endeavor of interest for future studies.

Reviewer #3 (Remarks to the Author):

I feel that this manuscript advances our knowledge of the early molecular events that lead to the development of DMGs. It merits publication and should enable further progress in follow up studies.

We thank the reviewer for this recognition of the novel contribution of our work.

The two general weaknesses of the manuscript are:

1) “*Although a lot of work has been done, and the manuscript provides some level of characterization of the molecular pathways that are dysregulated by the K27M mutation, the insights into why this happens in the brainstem as opposed to cortical or other brain regions is limited.”*

We thank the Reviewer for their candid feedback and honest assessment of the work. We agree that additional work is needed to completely understand the unknown reasons for the brainstem

preference of H3.3 K27M DMG. However, as pointed out by Reviewers 1 and 2, this is an unanswered mystery in the field. We note that this is the first demonstration of cell autonomous differences between brainstem and telencephalon OPCs in response to K27M, which we feel is a meaningful step in unraveling this mystery.

For deeper mechanistic understanding, we generated new ATAC-seq and ChIP-seq for H3K4me3 and H3K27me3 for control and K27M OPCs from brainstem and telencephalon as presented in revised **Figs. 6 and 7** (Results, pages 15-16, lines 432-453, and page 17, lines 479-497), with this data describing K27M-induced and regional variations in chromatin state and their impact on developmental signaling pathways. We found bivalently marked developmental signaling genes (BMP, WNT, Notch, TGFβ, integrin) are preferentially upregulated by K27M in brainstem OPCs (**Fig. 7**), providing a rational explanation for the exquisite sensitivity of this cell type and region for tumorigenesis with K27M mutations.

Additionally, we have contextualized this important finding in the Discussion, pages 19-20, lines 549-583:

"By integrating region-resolved *in vivo* imaging, single-cell and bulk transcriptomics, and epigenomic profiling with an *in vitro* OPC culture system, we mechanistically link regional developmental programs, bivalent chromatin, and the midline-restricted oncogenic potential of H3.3 K27M. At the chromatin level, K27M in DMG and neural stem cell models disproportionately affects genes whose promoters are bivalently marked by H3K4me3 and H3K27me3[10,14,24]. Consistent with these findings, here we show that K27M up-regulated genes in OPCs from both brainstem and telencephalon are enriched for bivalent promoters that lose H3K27me3 while retaining H3K4me3 and open chromatin at their transcription start sites. K27M simultaneously derepresses a subset of developmental programs while preserving repression of key patterning and identity transcription factors. The distribution of bivalent promoters is regionally patterned, suggesting that oncohistone-mediated re-programming will intersect distinct developmental networks in each region.

A central finding of our study is that K27M preferentially and robustly up-regulates multiple developmental signaling pathways in brainstem-derived OPCs. Bmp, Wnt, Notch, TGFβ, and integrin pathways play complex and varied roles in all stages of neurogenesis from stem cells through differentiation[52]. They are upregulated by K27M in brainstem-derived OPCs but intrinsically expressed throughout differentiation and not up-regulated by K27M in telencephalon-derived OPCs, reflecting context-dependent regional differences in developmental regulation. These regulators of signal transduction are collectively over-represented among K27M up-regulated, bivalently marked genes, and a majority of them show K27M-dependent up-regulation and/or promoter bivalency in human DMG models where K27M is knocked out or knocked down[24,43]. Bmp, Wnt, and Notch regulate glial lineage commitment, OPC proliferation, and myelination, and are linked to oncogenic behavior and invasion in DMG and other gliomas[18,22,53,54]. Genes within these pathways are also associated with super enhancers in patient-derived DMG cell lines[20], highlighting the disease relevance of the K27M-induced signatures in the brainstem oligodendrocytic lineage.

Our data support a model in which K27M lifts PRC2 repression at a brainstem-biased set of bivalent developmental genes, igniting aberrant, self-reinforcing signaling that sustains an immature, proliferative OPC-like state and may affect motility, adhesion, and axonal or synaptic interactions. Additional studies are necessary to better understand the regionally selective impacts of K27M on these pathways, how they relate to normal oligodendroglial development, and how they can be effectively targeted for therapeutic benefit in DMG."

2) While the manuscript is well structured and thoughtfully argued, I found it to be heavy on the jargon, long, and difficult to understand. As a reader I would have appreciated a simpler language accessible to a wider audience.

We have restructured for clearer narrative flow, simplified the language, and removed jargon throughout the manuscript. Abbreviations (e.g. t-cOPC and cCC) have been replaced with descriptive language (e.g. cultured OPCs from telencephalon and caudal corpus callosum, respectively) and redundant methodological descriptions removed from the Results. We hope these significant efforts improve reader accessibility.

As a reviewer, I find that this type of manuscript is difficult to properly review in two weeks. It is quite long with a great deal of supplementary data. Because of the range of techniques used, it needs to be reviewed by experts from several fields. Given the time constraints and my area of expertise, while I tried to follow most of the logic, I focused on the genomics. I am overall satisfied that the authors know what they are doing and have carried out appropriate analyses. I have only one minor comment:

Differential abundance testing, Figure S3. How was this done, what method was used? Intuitively, looking at figure S3E it seems like there are substantial differences at P4, for example in neuroblasts. Please provide information on the methodology and statistics, also justifying the choice of method. There are many "abundance testing" approaches and they do not always agree.

In brief, differential abundance testing was performed on the proportions of cell types in each sample using quasi-likelihood methods from the edgeR package. Testing was performed between H3 genotypes for each age individually and limited to cells of the neuronal, glial, or progenitor lineages in which the mutation was induced. This approach allows us to model overdispersed counts to better account for sample-to-sample variability within each cell type with limited replicates.

We agree that the differences in the original **Supplementary Fig. 3e** (now **Supplementary Fig. 8e**) can initially seem substantial. However, none of the comparisons within any timepoint yielded an FDR < 0.05. As only the average proportions for each group are plotted, the variability for the proportion of each cell type in each group is not immediately apparent, but this variability is considered during the fitting of the negative binomial generalized linear model used for testing. Differential abundance testing with single cell data is fraught with confounding factors, including composition effects, low counts of rare populations, and sampling variation.

To elaborate, dissection of specific tissues or regions can be technically difficult and result in variable inclusion of cell types that are not of interest, e.g. dura or meninges in brain dissections. In addition, certain cell types may be more susceptible to loss or damage during tissue dissociation, and this may vary across development particularly with whole cell isolation protocols such as those used here. These purely technical changes in composition coupled with the use of total cell number during normalization can yield misleading results. We focused only on lineages of interest for these analyses to mitigate this confounding factor, but the small numbers of cells for certain populations, e.g. preOPCs, coupled with relatively low sample size in each group (n=3-4) results in modest power for heterogeneous samples with subtle differences such as those in this study.

In sum, these caveats emphasize that scRNA-seq is an unreliable tool to assess small or subtle shifts in cell type compositions unless these confounding factors are mitigated by many replicates, bulk whole tissue dissociation to minimize technical sampling variation, and an iterative analysis approach to deal with composition effects stemming from real changes. As such, we relied on orthogonal methods such as immunofluorescence imaging of matched tissue and cleared whole brains and careful analyses of bulk RNA-seq of synchronized OPC cultures to more accurately assess *in vivo* shifts in cellular composition (**Figs. 1-2**) and *in vitro* changes in differentiation dynamics (**Figs. 4, 5, Supplementary 12**), respectively.

The details for this analysis were mistakenly left out of the methods section in our original submission. They have been added to a new section titled “Differential abundance testing” (Methods, page 37, lines 1075-1083), and we apologize for the oversight.

Response to Reviewers

We again thank the reviewers for their thoughtful and constructive feedback. The manuscript was significantly improved by incorporating their suggestions.

REVIEWERS' COMMENTS

Reviewer #1 (Remarks to the Author):

the authors have addressed all my comments. This is an exciting manuscript that represents an important contribution to the field.

My only minor comment relates to the wording of one sentence in the introduction: adaptive myelination continues throughout life and contributes to learning, memory and motor functions. I recommend rewording lines 88-89 from "…adaptive myelination in response to learning, memory, and motor function continuing into young adulthood" to more accurately read, "…adaptive myelination in response to neuronal activity continuing into adulthood and contributing to learning, memory, and motor functions."

This sentence has been adjusted as suggested.

Reviewer #3 (Remarks to the Author):

This is a re-review of a revised version of the original manuscript. I appreciate that the authors have taken the time to address my comments. I have no further concerns and recommend the manuscript for publication.
