# Supplementary material for: Cell autonomous regional differences in oligodendrocyte lineage development and responses to oncohistone H3.3 K27M
Source: Nat Commun. 2026 Aug 26;17:8811. doi: 10.1038/s41467-026-76468-6 (PMC13518833; doi:10.1038/s41467-026-76468-6)
Supplement: Supplementary file 3 [file 41467_2026_76468_MOESM1_ESM.pdf]

Cell autonomous regional differences in oligodendrocyte lineage development and responses to oncohistone H3.3 K27M

Supplementary Information

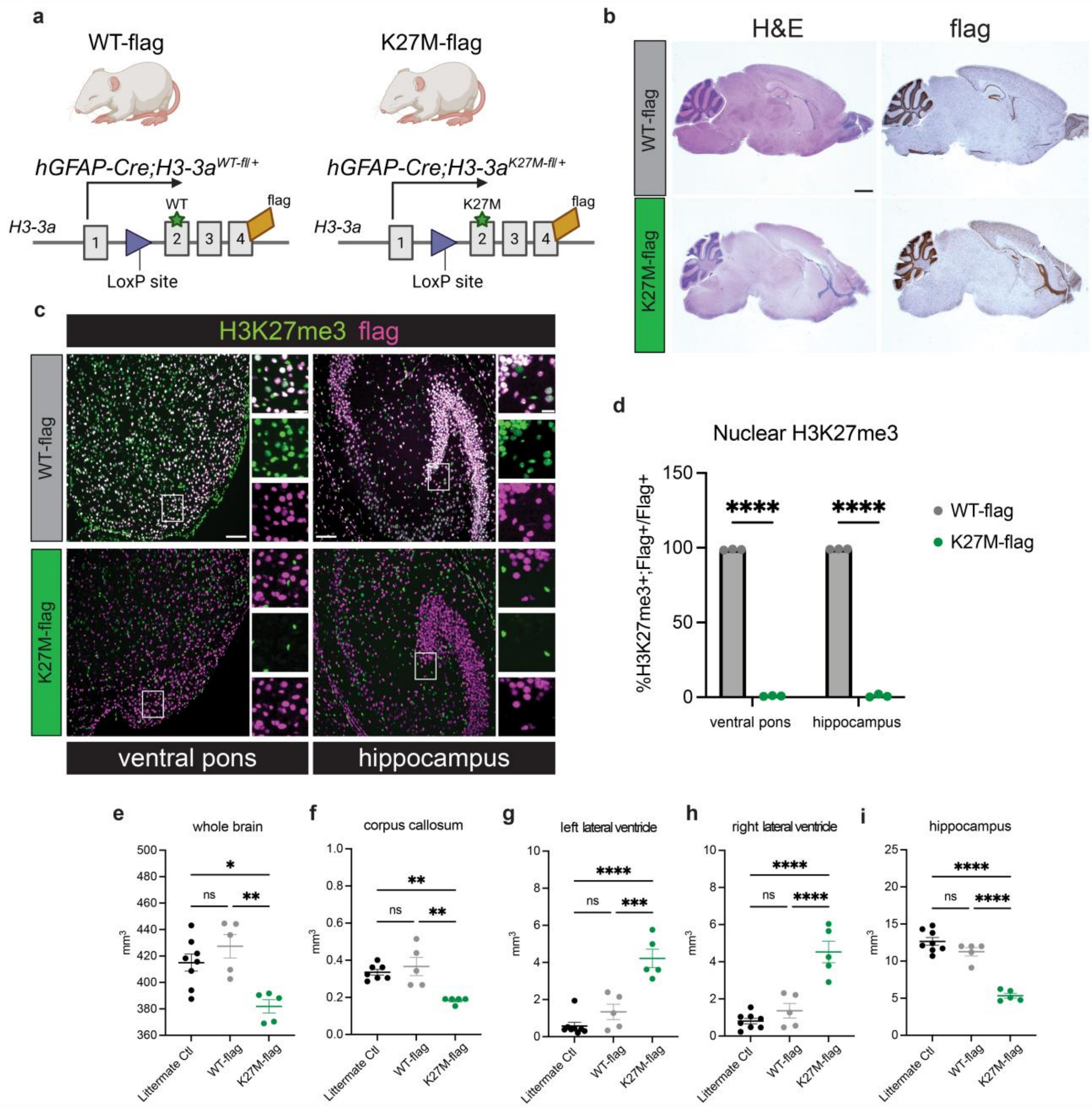

Supplementary Figure 1. Expression of H3.3 K27M in embryonic NSPCs leads to distinct regional neurodevelopmental defects. a Diagram of knock-in *H3-3a$^{WT\text{-}fl/+}$* (WT-flag) and *H3-3a$^{K27M\text{-}fl/+}$* (K27M-flag) mice utilized to assess hGFAP-Cre activity. Diagram adapted from Larson et al, 2019. Created in BioRender. Dobson, C. (2026) https://BioRender.com/ctnq88r. **b** Representative H&E (left) and Flag immunohistochemistry (right) images in WT-flag and K27M-flag mice at P21. Scale bar is 500μm. **c** Representative immunofluorescence images for Flag and H3K27me3 in the ventral pons (left) and hippocampus (right) in WT-flag and K27M-flag mice at P14. High magnification insets show overlay, H3K27me3 and Flag signals in boxed areas. Scale bar is 100μm, and 20μm in high magnification insets. **d** Quantification of percentage of Flag+ cells that are H3K27me3+ in the ventral pons and hippocampus in WT-flag and K27M-flag mice at P14 (n=3 per group; Student's unpaired, two-tailed t-test. $p < 0.0001$: ****). **e**-**i** MRI measurements of the volume of the whole brain (**e**), corpus callosum (**f**), left lateral ventricle (LV; **g**), right LV (**h**), and hippocampus (**i**) from P21 K27M-flag (n=5), WT-flag mice (n=5), and littermate controls (n=8, except corpus callosum where n=7). One-way ANOVA with Tukey Test. ns=not significant, $^{**}p < 0.005$: **, $p < 0.0005$: ***, $p < 0.0001$: ****. For (**d**-**i**), each point represents a measurement from one mouse with error bars showing mean±SEM. Source data are provided as a Source Data file.

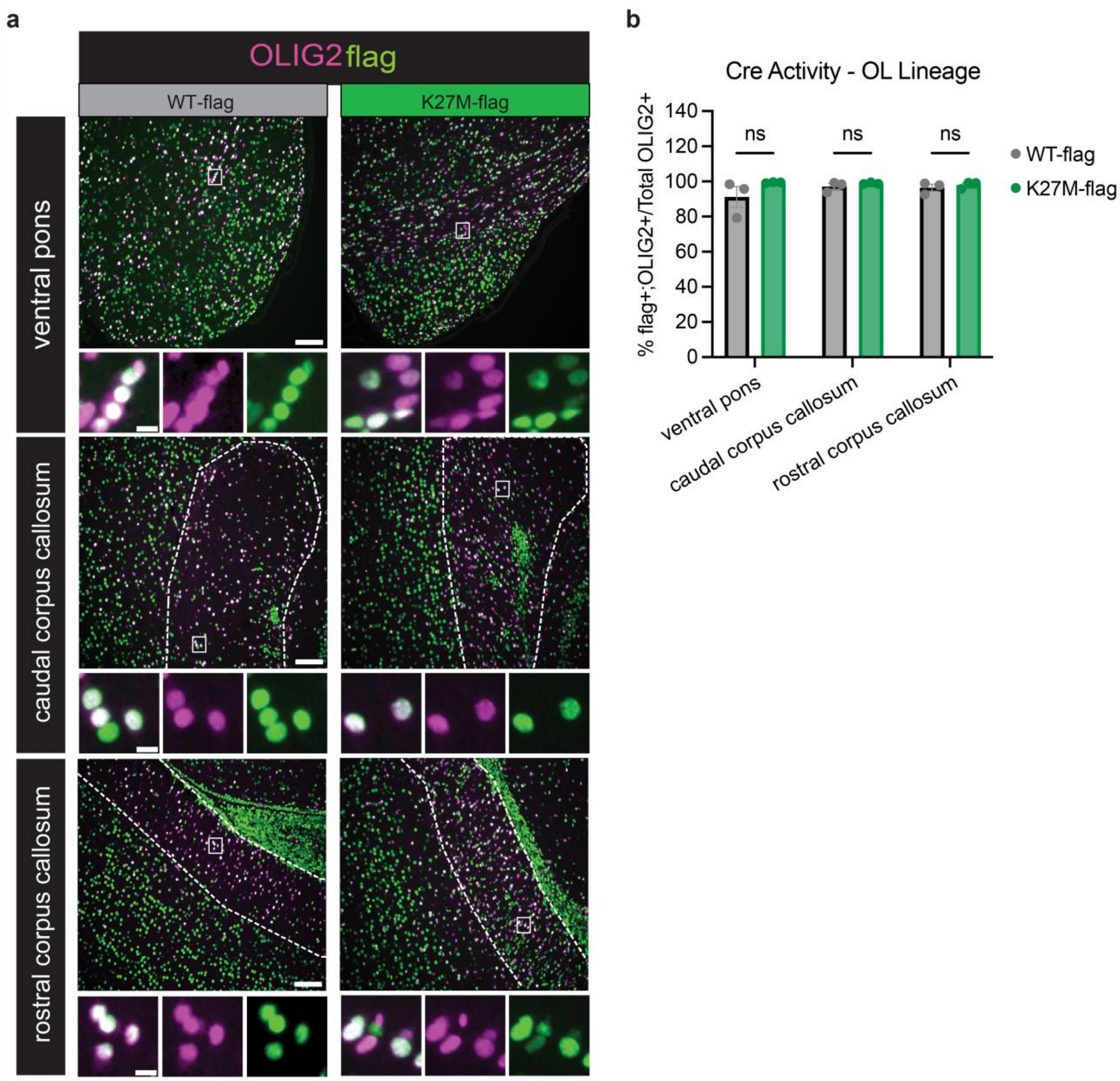

Supplementary Figure 2. hGFAP-Cre results in Cre recombination in OLIG2+ oligodendrocyte (OL) lineage. a Representative IF images for flag and OLIG2 in the ventral pons, caudal corpus callosum, and rostral corpus callosum in WT-flag and K27M-flag mice at P14. Scale bars are 100μm, or 10 μm for high magnification insets of boxed regions. **b** Quantification of the OL-lineage Cre activity (percentage of OLIG2+ cells that are flag+) in the ventral pons, caudal corpus callosum, and rostral corpus callosum in WT-flag and K27M-flag mice at P14 (n=3). Each point on the graph represents a single mouse with error bars showing mean ± SEM. Student's unpaired, two-sided t-test. ns = not significant. Source data are provided as a Source Data file.

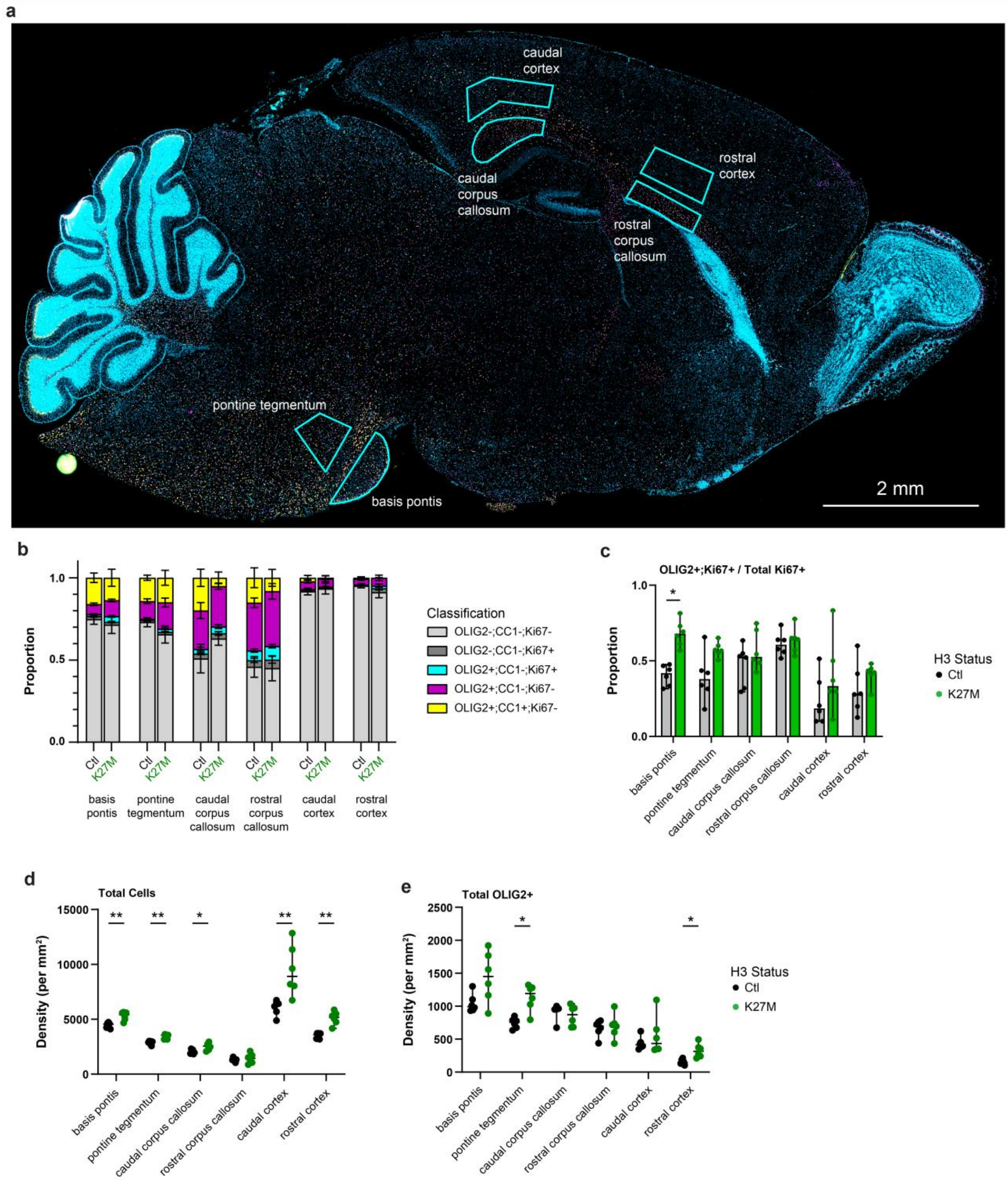

Supplementary Figure 3. H3.3 K27M alters oligodendrocyte lineage (OL) composition and cell density. a Representative OLIG2/CC1/Ki67/DAPI immunofluorescence image of P14 control mouse displaying quantified areas. **b** Average proportions of each cell classification for each genotype and region. Error bars indicate mean ± 95% CI. **c** Quantification of proportion of proliferating OPCs in total proliferating cells in different brain regions in Ctl and K27M mice at P14 (n=6; two-sided Mann-Whitney U tests with Benjamini-Hochberg adjustment). **d-e** Density of total cells (**d**) or OLIG2+ cells (**e**) across brain regions from Ctl and K27M mice at P14 (n=6; two-sided Mann-Whitney U tests with Benjamini-Hochberg adjustment). For **c-e,** each point represents average for 2 sequential slices from a mouse with error bars indicating median ± 95% CI; p.adj < 0.05: *; p.adj < 0.01: **; p.adj < 0.001: ***; full data and statistics in **Supplementary Data 1**. Source data are provided as a Source Data file.

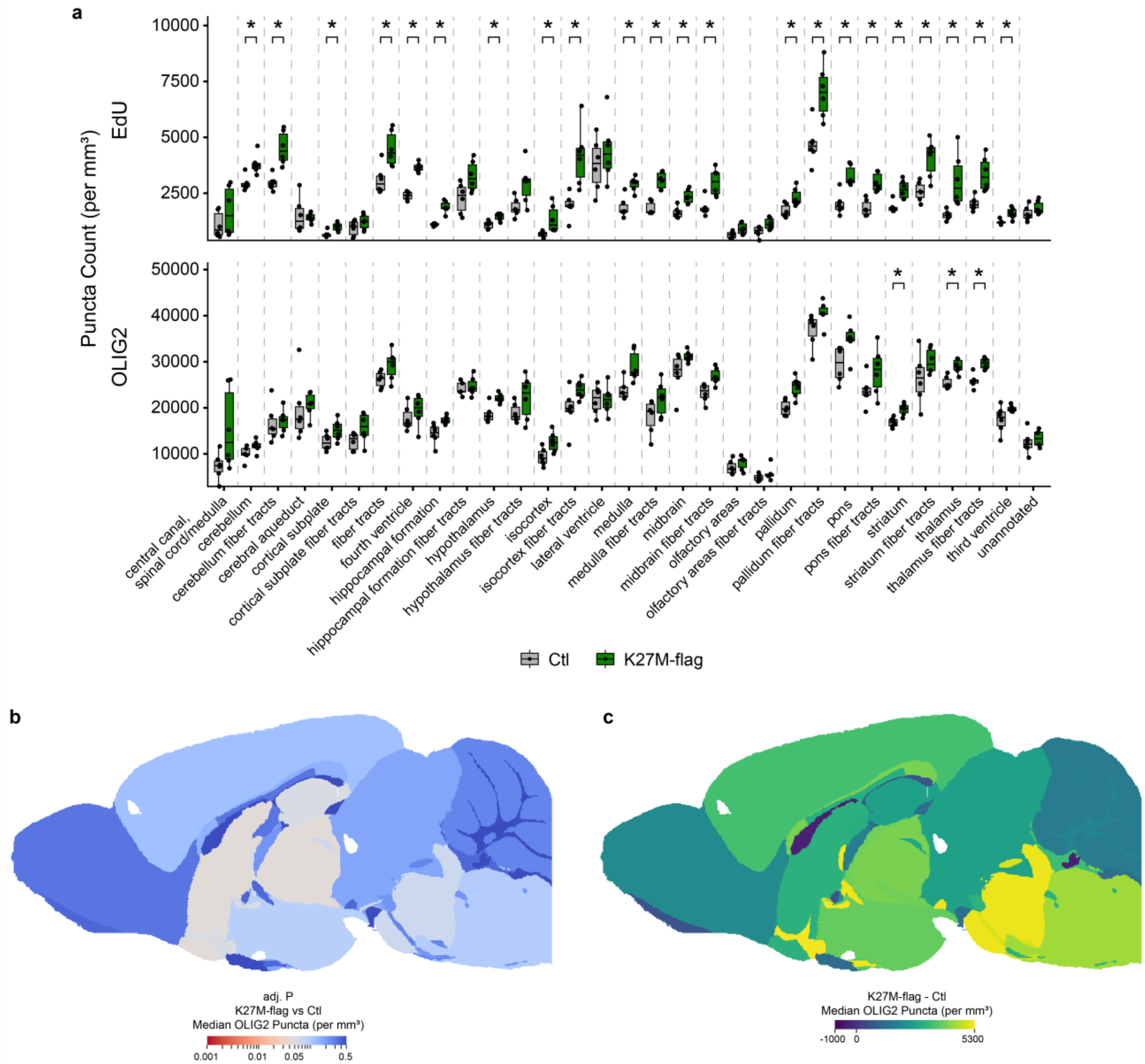

Supplementary Figure 4. H3.3 K27M does not broadly expand the oligodendrocyte lineage. a Edu and Olig2 puncta quantifications for whole cleared brains from P14 K27M-flag and Ctl mice (n=6 per group) per region. Two-sided Mann-Whitney U test with Benjamini-Hochberg adjustment; p.adj < 0.05: *. For boxplots, center line, median; box limits, upper and lower quartiles; whiskers, 1.5x interquartile range. **b** Per region adjusted p-values comparing volume-normalized Olig2 puncta counts for P14 K27M-flag and Ctl (n=6 per genotype). **c** Per region difference in median volume-normalized Olig2 puncta count between K27M-flag and Ctl mice**.** Source data are provided as a Source Data file.

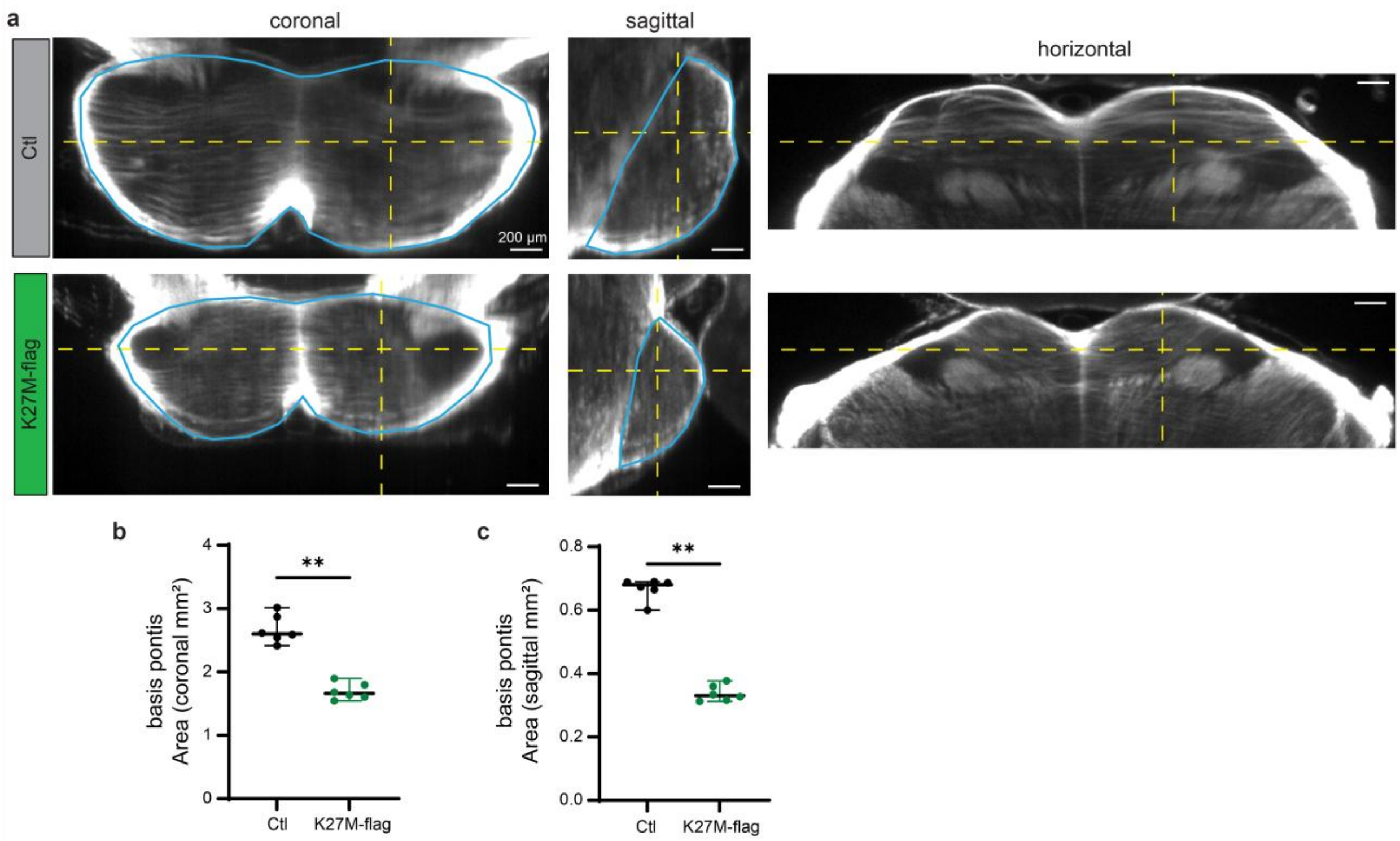

Supplementary Figure 5. H3.3 K27M reduces basis pontis size. a Representative images of CNPase architecture of the basis pontis from cleared whole brain imaging with CNPase staining of Ctl and K27M-flag P14 mice. Dotted yellow lines indicate planes of images while blue outlines indicate quantified areas. Scale bar is 200 µm. **b-c** Quantification of the area of the basis pontis determined by CNPase+ architecture in P14 K27M-flag and Ctl mice (n=6) from a coronal (**b**) or sagittal (**c**) plane. For **b-c**, each point represents basis pontis area with error bars indicating median $\pm$ 95% CI; Two-sided Mann-Whitney U test; **b** and **c** p=0.0022. Source data are provided as a Source Data file.

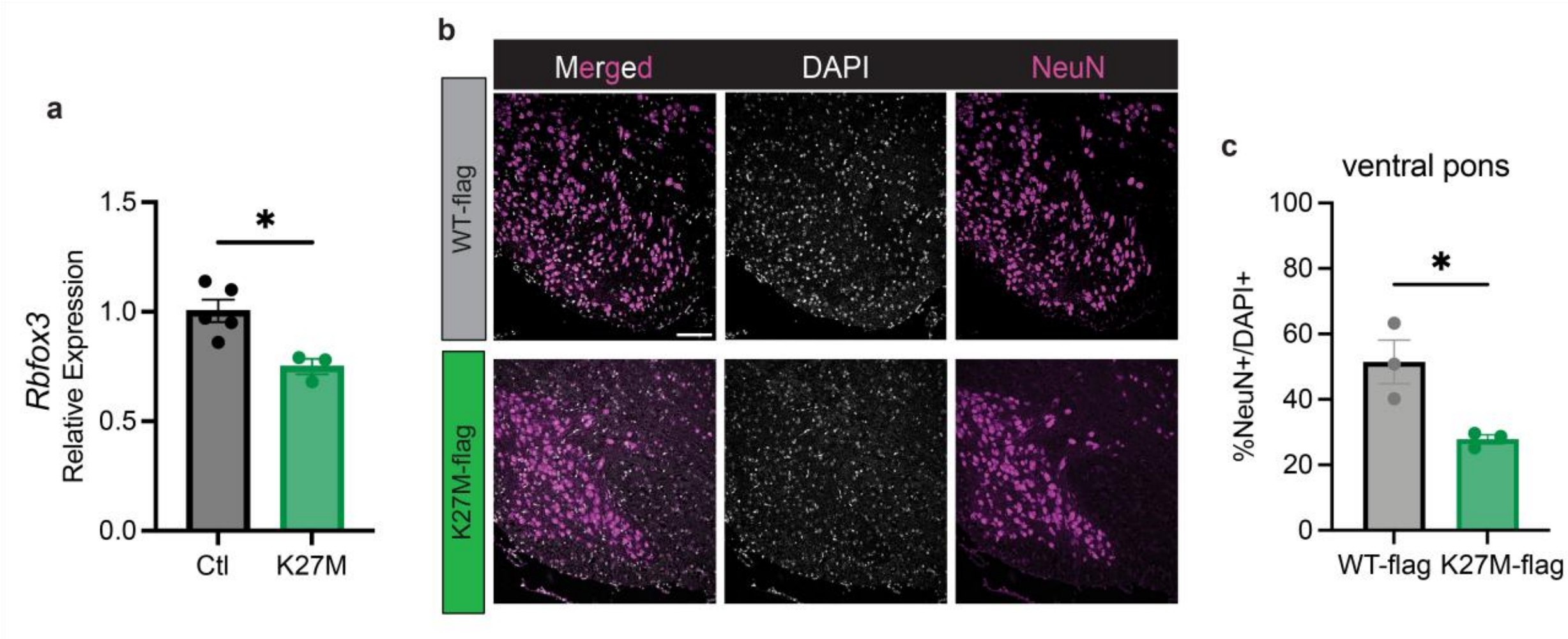

Supplementary Figure 6. H3.3 K27M reduces NeuN+ neurons in the basis pontis. a qRT-PCR of *Rbfox3* (NeuN gene) mRNA expression in P14 brainstem tissue of Ctl (n=5) and K27M mice (n=3). Student's unpaired, two-sided t-test, p=0.0131. **b** Representative IF images of DAPI and NeuN in the ventral pons of WT-flag and K27M-flag mice at P21. Scale bar is 1000 µm. **c** Blinded quantification of percentage of DAPI nuclei that are NeuN+ in the ventral pons in WT-flag and K27M-flag mice at P21 (n=3). Student's unpaired, two-sided t-test, p=0.0258. For **a** and **c**, each point on the graphs represents a measurement from one mouse with error bars showing mean ± SEM; $p < 0.05$: *. Source data are provided as a Source Data file.

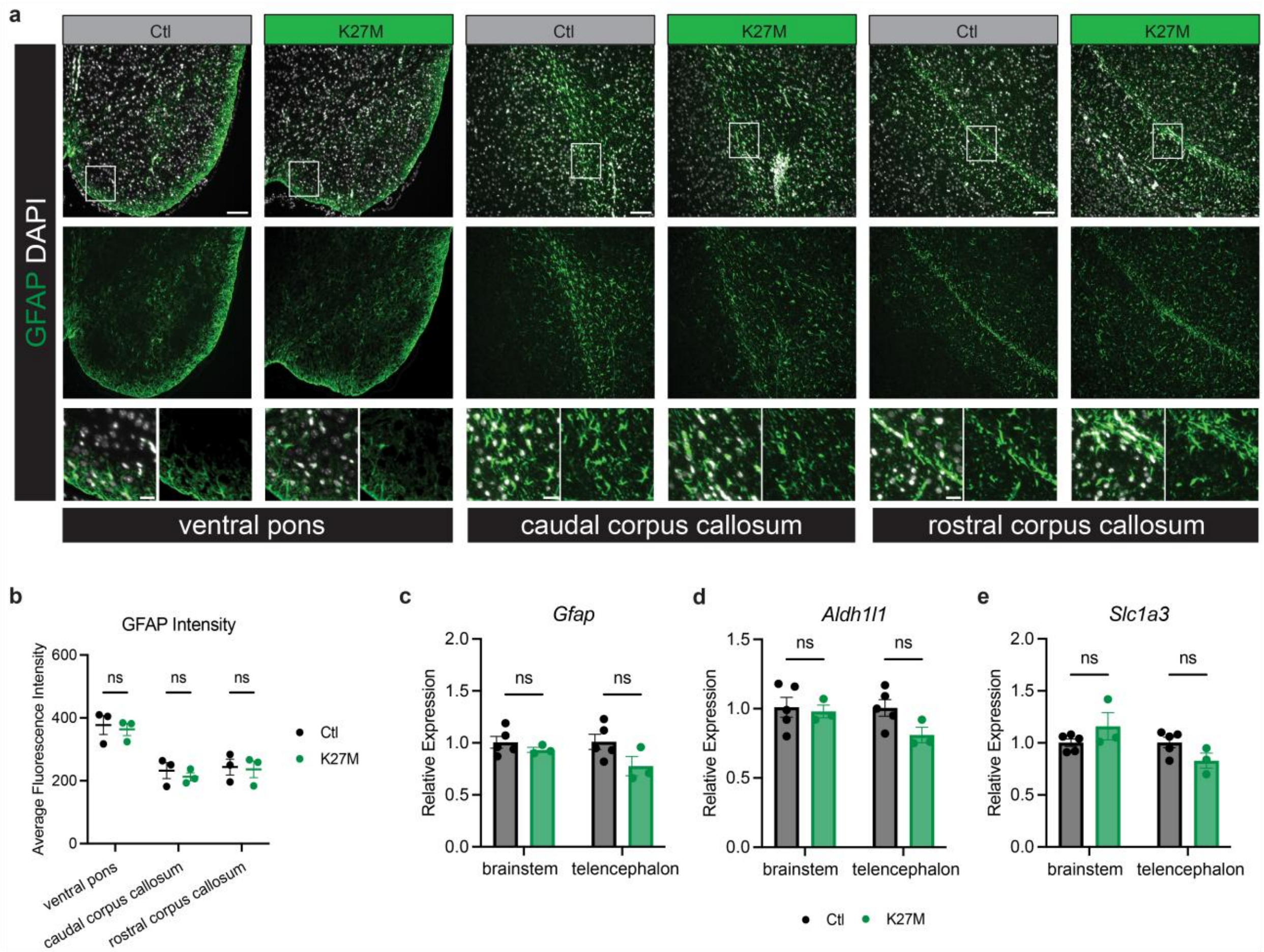

Supplementary Figure 7. H3.3 K27M does not alter early postnatal expression of astrocyte markers. a Representative IF images of DAPI (grey) and GFAP (green) in the ventral pons, and caudal and rostral corpus callosum in Ctl and K27M mice at P14. Scale bars are 100 µm, or 20 µm for the boxed high magnification insets. **b** Quantification of average GFAP fluorescence intensity in the ventral pons, caudal corpus callosum, and rostral caudal callosum region (n=3). **c**-**e** qRT-PCR of *Gfap* (**c**), *Aldh1l1* (**d**) and *Slc1a3* (**e**) mRNA expression in Ctl (n=5) and K27M (n=3) brainstem and telencephalon tissues at P14. For (**b**-**e**), each point on the graph represents a measurement from one mouse with error bars showing mean ± SEM. Student's unpaired, two-sided t-test. ns = not significant. Source data are provided as a Source Data file.

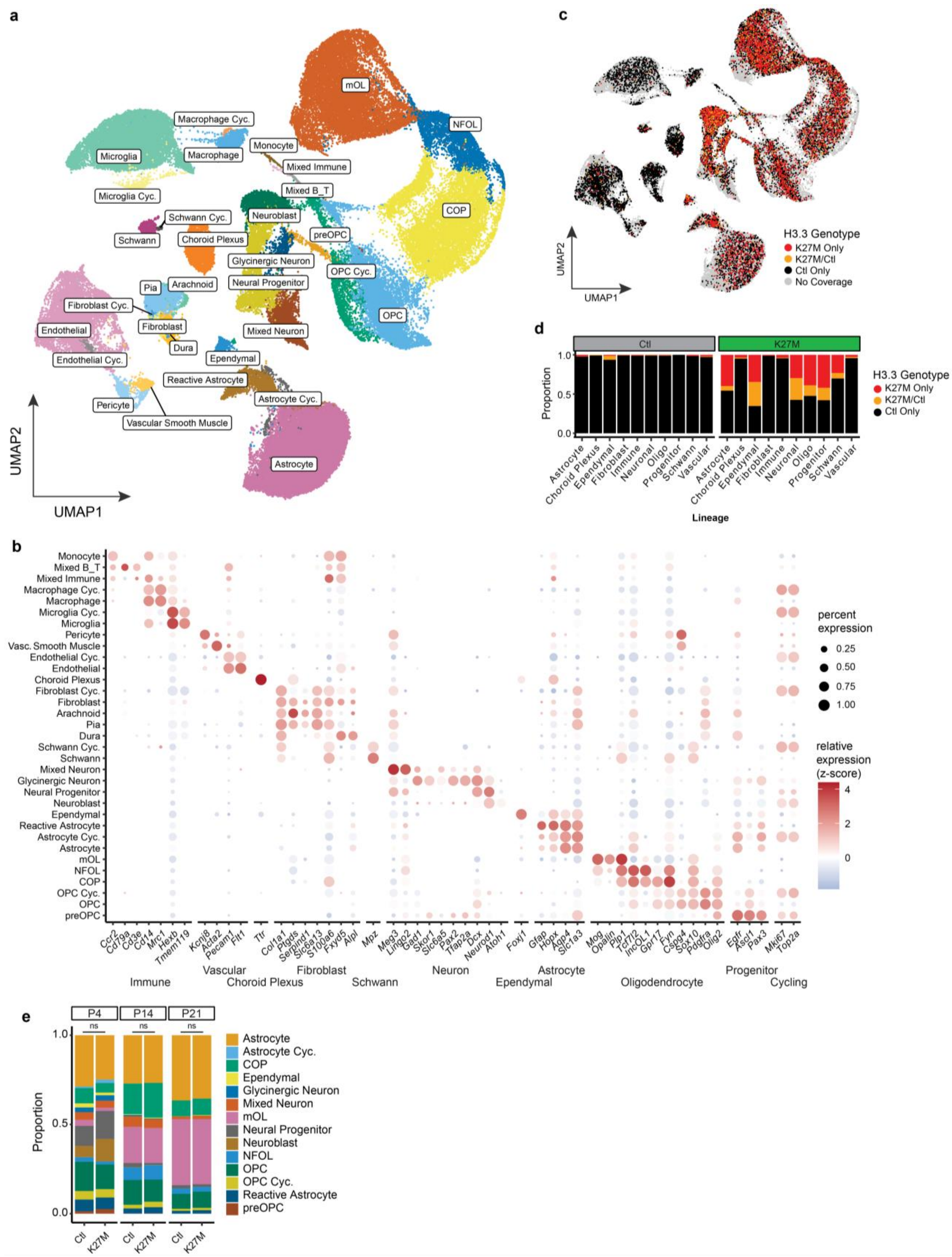

Supplementary Figure 8. H3.3 K27M does not generate unique cell populations or broadly skew cell proportions in the brainstem. a UMAP of cells from P4, P14, and P21 mouse brainstems (n=4 (P14, P21) or n=3 (P4) per age per genotype) with or without H3.3 K27M with cell type annotations shown. Pre-oligodendrocyte

precursor cell (pre-OPC), oligodendrocyte precursor cell (OPC), committed oligodendrocyte precursor (COP), newly formed oligodendrocyte (NFOL), and mature oligodendrocyte (mOL). **b** Dotplot displaying cell type markers across cell types. **c** UMAP displaying genotypes for the H3.3 K27M allele in each cell. **d** Stacked barplots showing the proportion of cells with reference and/or alternate alleles at the H3.3 K27 position across cell types and sample ages. Only cells with coverage at the allele were included. **e** Stacked barplots displaying the average proportions of cell types for the astrocytic, oligodendritic, and neuronal lineages across ages and genotypes. Differential abundance testing performed between genotypes for each age (two-tailed F-test with quasi-likelihood methods). ns = not significant. Source data are provided as a Source Data file.

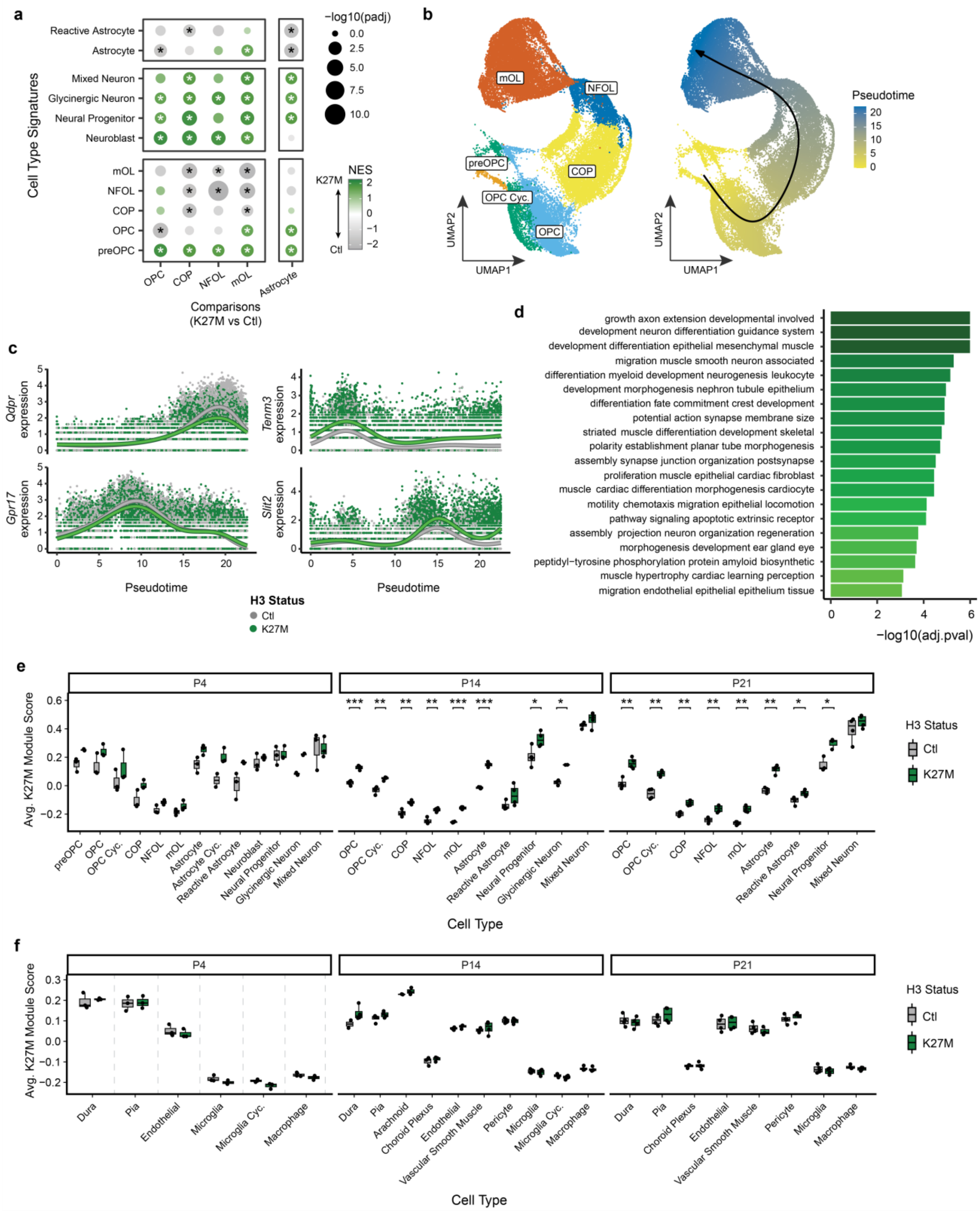

Supplementary Figure 9. H3.3 K27M glial cells retain transcriptional reflections of prior cell states and intrinsically drives neurogenic programs in the brainstem. a Summary dot plot of GSEA results from K27M vs control comparisons using control cell type marker gene sets (**Supplementary Data 4**). In order from least to most differentiated: pre-oligodendrocyte precursor cell (pre-OPC), oligodendrocyte precursor cell (OPC), committed oligodendrocyte precursor (COP), newly formed oligodendrocyte (NFOL) and mature oligodendrocyte (mOL). **b** UMAPs of oligodendritic lineage from preOPCs to mOLs (left) and pseudotime (right) along the differentiation

trajectory. **c** Scatter plots showing expression of representative oligodendrocyte differentiation (*Gpr17* adj.p=0, *Qdpr* adj.p=0) and neuronal genes (*Tenm3* adj.p=0, *Slit2* adj.p=0) identified as significant (Two-tailed Wald test with Benjamini-Hochberg adjustment; p.adj < 0.05; effect size threshold of log2(1.25); **Supplementary Data 5**) in K27M vs control in oligodendrocyte-lineage cells along pseudotime. Cells colored by H3 status. Smoothers are plotted for each group fit to 100 points along pseudotime. **d** Summary bar chart of 5 most frequent words in GO biological process terms from one-tailed hypergeometric enrichment test with Benjamini-Hochberg adjustment of 156 genes up-regulated by K27M (quasi-likelihood F-tests with Benjamini-Hochberg adjustment; p.adj < 0.05; log2 FC > 0.2; 3 different comparisons; **Supplementary Data 3,6**). GO terms clustered by semantic similarity with a threshold of 0.7 and the top -log10(adjusted p-value) for cluster members plotted. **e** Boxplots showing the per-sample average module scores of the robust K27M up-regulated gene set between genotypes in K27M-induced cell types at indicated timepoint (see **Supplementary Fig. 8c-d**; 156 genes; **Supplementary Data 6**). **f** Same as in (**e**) but for cell types in which K27M was not induced. Two-sided Student's t-tests used between genotypes for each cell type and adjusted p-values generated via Benjamini-Hochberg adjustment within each timepoint panel. p.adj < 0.05: *; p.adj < 0.01: **; p.adj < 0.001: ***; p.adj < 0.0001: ****. For boxplots, center line, median; box limits, upper and lower quartiles; whiskers, 1.5x interquartile range. Source data are provided as a Source Data file.

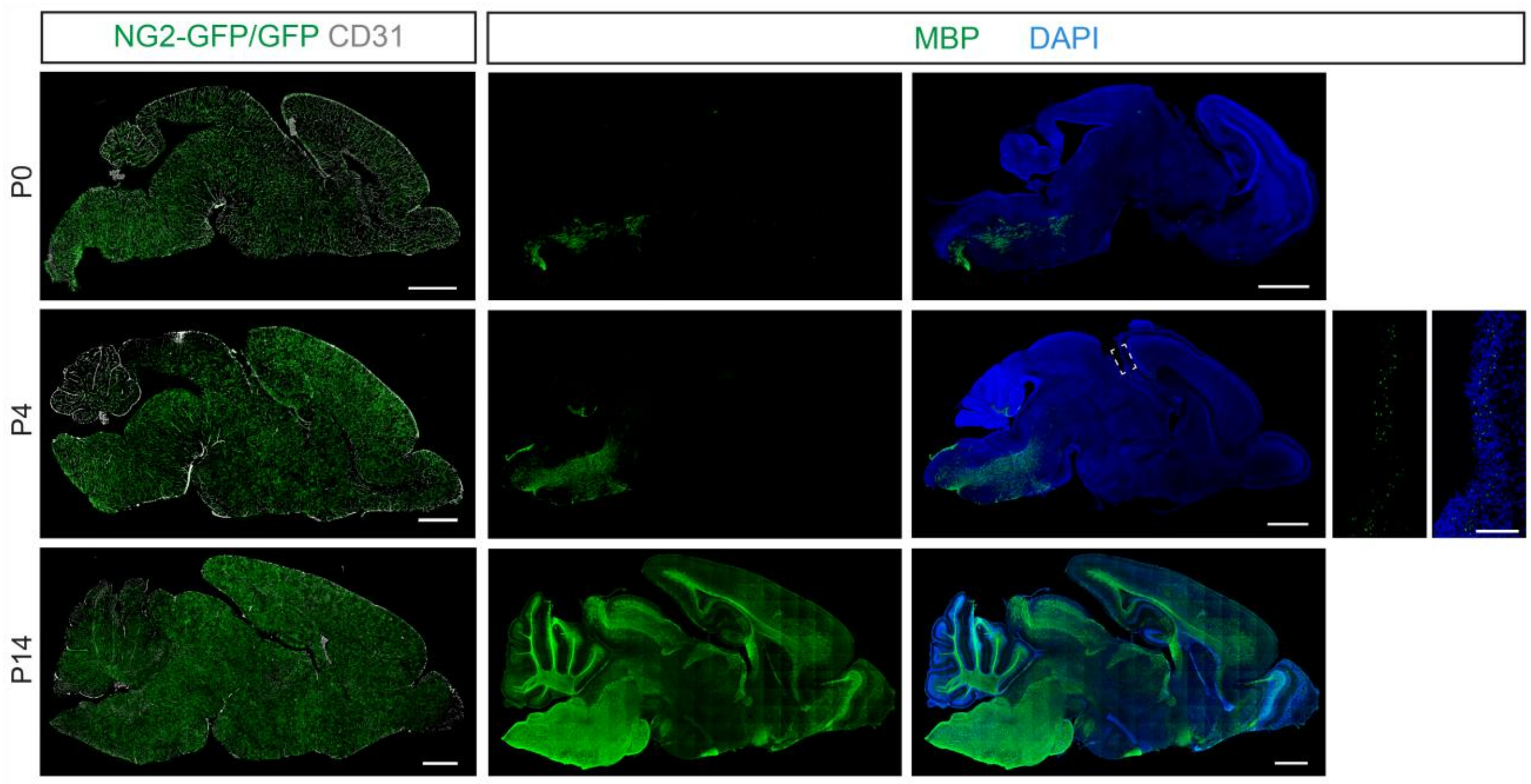

Supplementary Figure 10. Spatiotemporal differences in OPC development and differentiation *in vivo*. Representative images of immunofluorescence for NG2-GFP, CD31 (marking pericytes which also express NG2) (left), and MBP (right) from the developing mouse brain at P0, P4, and P14 (50 μm thick free-floating sections). Scale bar is 1 mm for whole-section images and 100 μm for high magnification images. Source data are provided as a Source Data file.

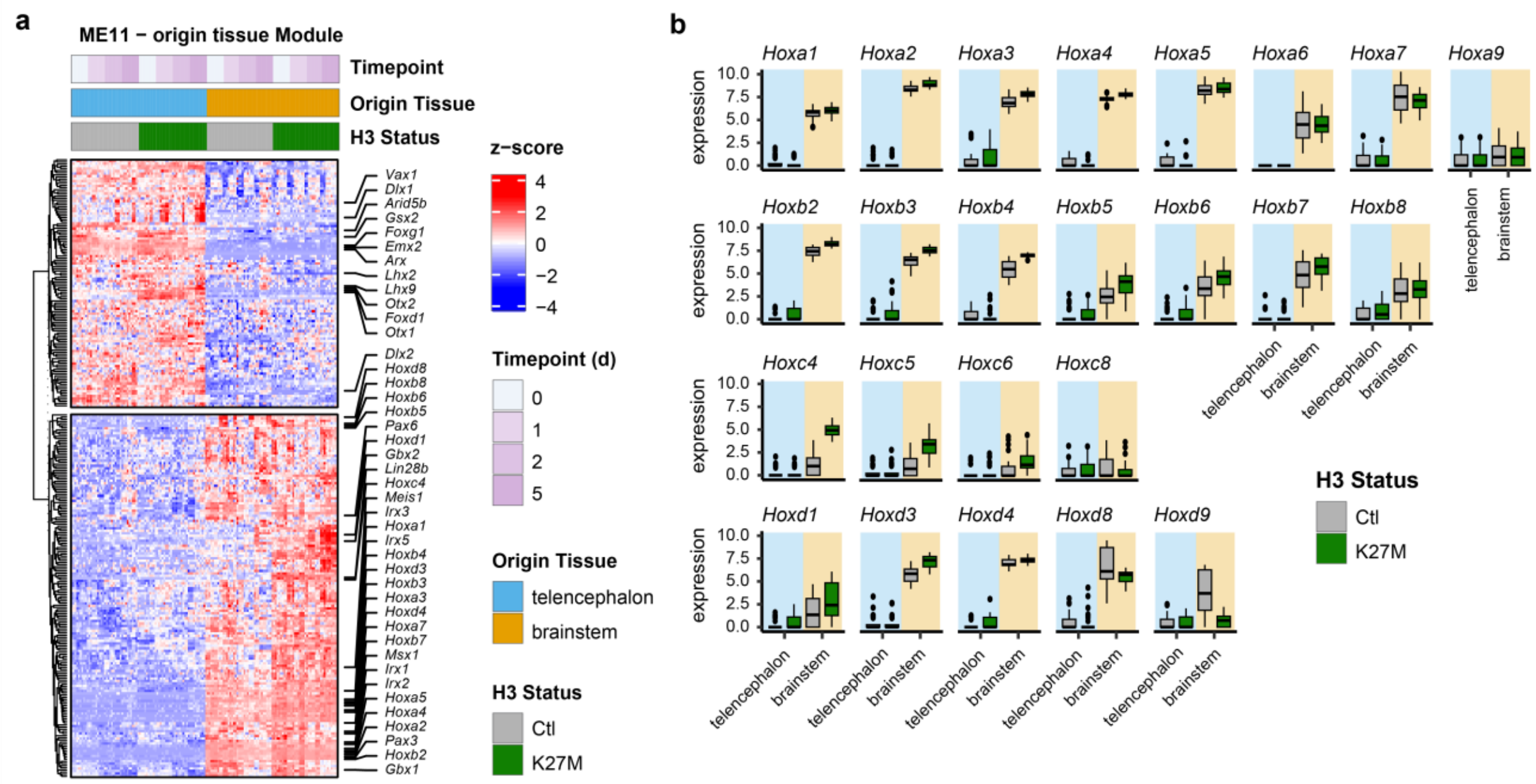

Supplementary Figure 11. Regional identity transcriptional programs persist through *in vitro* OPC differentiation. a Heatmap of genes in origin tissue-associated gene module 11 from WGCNA analysis (from **Fig. 3g**; **Supplementary Data 7**) with select genes labeled. **b** RNA-seq boxplots of Hox family genes. For each gene, all timepoints were included in each brain location/genotype group (n=35). Expression values plotted are log2(normalized counts + 1). For boxplots, center line, median; box limits, upper and lower quartiles; whiskers, 1.5x interquartile range; outliers shown.

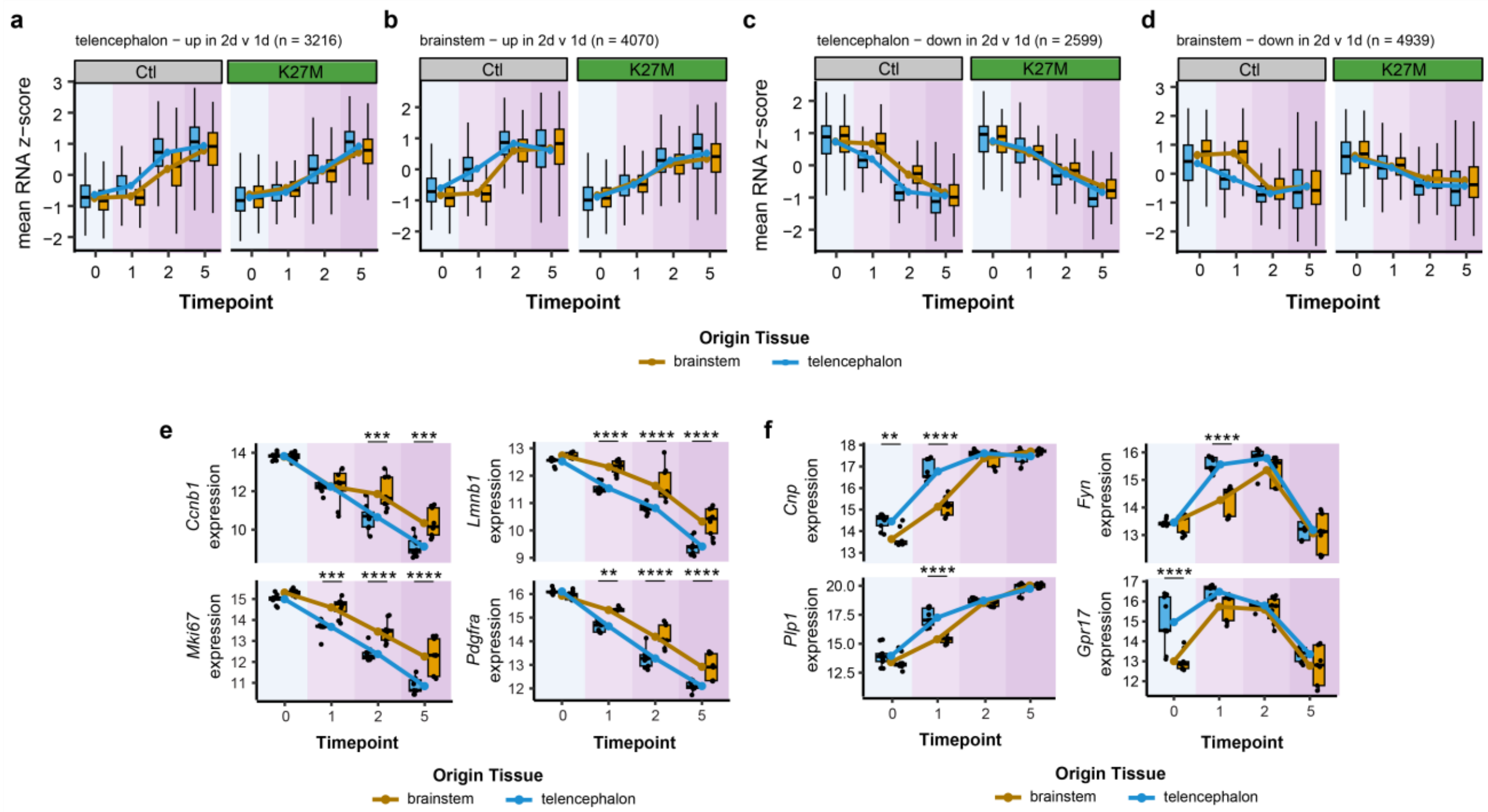

Supplementary Figure 12. Telencephalon-derived OPCs are primed for differentiation while brainstem-derived OPCs retain a more proliferative state. a-b Boxplots showing the average expression z-score for up-regulated genes (two-tailed Wald test, Benjamini-Hochberg adjustment, p.adj < 0.05, effect size testing threshold |FC| > 1.1; **Supplementary Data 8**) between the 2-day and 1-day timepoints in telencephalon-derived (**a**) or brainstem-derived (**b**) control OPC cultures across differentiation. The average for each group is connected via a lineplot. The same genes are displayed for H3.3 K27M cultures (right). **c-d** The same as in (**a-b**) but for genes down-regulated through differentiation in control cultures. **e-f** RNA-seq boxplots for select known proliferation (*Ccnb1*, *Mki67*) and OPC-associated (*Lmnb1*, *Pdgfra*) genes (**e**) or committed oligodendrocyte precursor (*Fyn*, *Gpr17*) and mature oligodendrocyte markers (*Plp1*, *Cnp*) genes (**f**) in OPC control cultures across differentiation timepoints between origin tissue. Expression values plotted are log2(normalized counts + 1). Differentially expressed genes (two-tailed Wald test, Benjamini-Hochberg adjustment, p.adj < 0.05, effect size testing threshold |FC| > 1.1; **Supplementary Data 8**) between groups are indicated by asterisks. p.adj < 0.05: *; p.adj < 0.01: **; p.adj < 0.001: ***; p.adj < 0.0001: ****. For boxplots, center line, median; box limits, upper and lower quartiles; whiskers, 1.5x interquartile range. Source data are provided as a Source Data file.

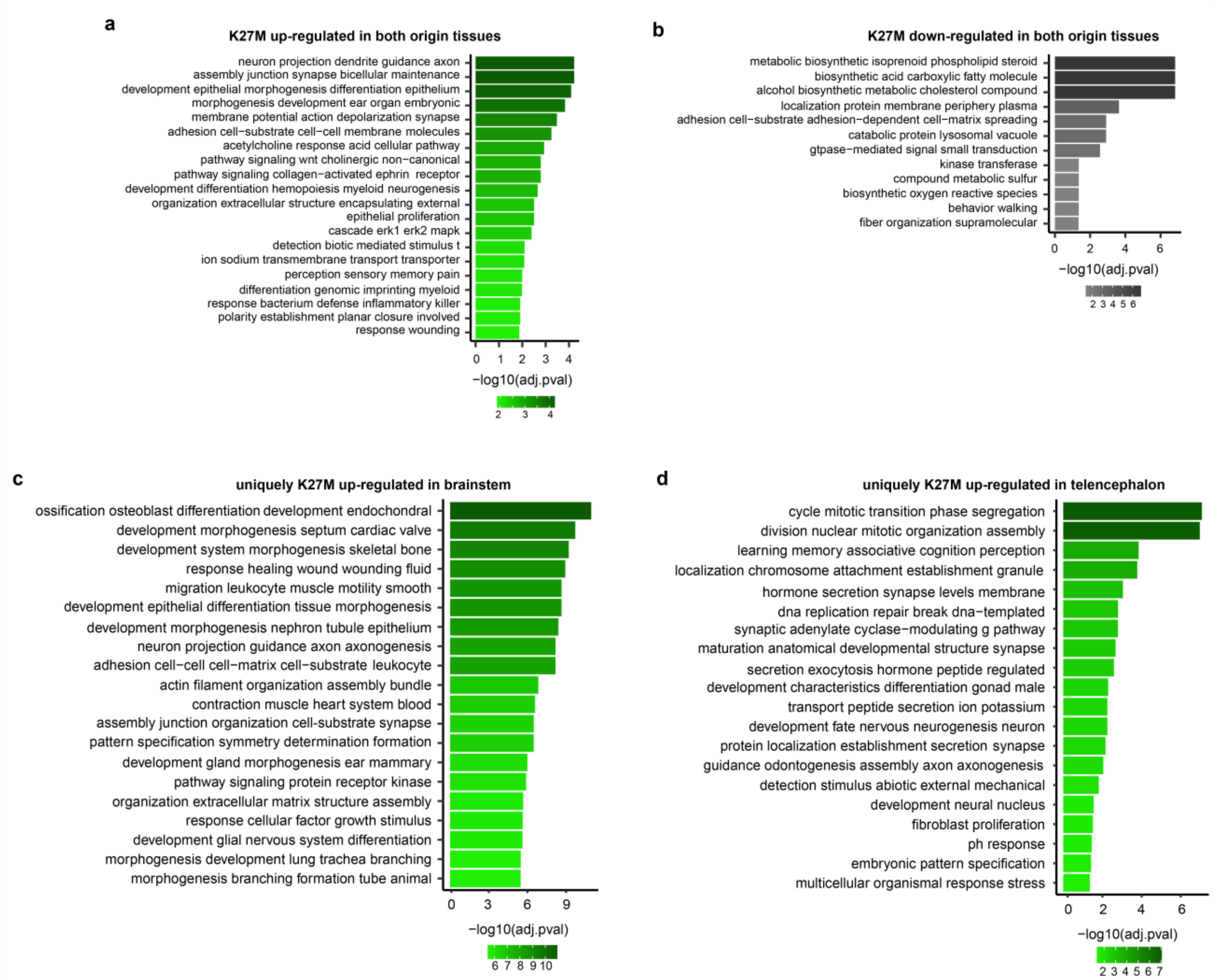

Supplementary Figure 13. H3.3 K27M-induced expression changes in the OL lineage exhibit regional selectivity. a Summary of top words describing GO biological process terms identified by enrichment testing of genes commonly up-regulated (two-tailed Wald test, Benjamini-Hochberg adjustment, p.adj < 0.05, effect size testing threshold FC > 1.1) in K27M vs control OPC cultures across any timepoint in both origin tissues. GO terms were clustered by semantic similarity with a threshold of 0.7, the top 5 most frequent words for each cluster were used as labels, and the top -log10(adjusted p-value) (one-tailed hypergeometric enrichment test with Benjamini-Hochberg adjustment) for the cluster members is plotted. Word frequency was derived from all terms in each cluster with common stopwords removed. **b** Same as in (**a**) but for K27M commonly down-regulated genes in both origin tissues across any timepoints. **c** Same as in (**a**) but for genes uniquely up-regulated in K27M vs control OPC cultures across any timepoint, specifically in brainstem OPC cultures. **d** Same as in (**c**), but for genes uniquely up-regulated in K27M vs control, specifically in telencephalon OPC cultures. Source data are provided as a Source Data file.
