# Supplementary material for: Cell autonomous regional differences in oligodendrocyte lineage development and responses to oncohistone H3.3 K27M
Source: Nat Commun. 2026 Aug 26;17:8811. doi: 10.1038/s41467-026-76468-6 (PMC13518833; doi:10.1038/s41467-026-76468-6)
Supplement: Supplementary file 4 [file 41467_2026_76468_MOESM2_ESM.pdf]

Description of Additional Supplementary Files

File name: Supplementary Video 1
Description: Video of cleared whole brain of P14 littermate control mouse with SYTO16 (green) and EdU-ClickiT labeling (magenta).

File name: Supplementary Video 2
Description: Video of cleared whole brain of P14 K27M-flag mouse with SYTO16 (green) and EdU-ClickiT labeling (magenta).

File name: Supplementary Data 1
Description: Immunofluorescence image quantifications and statistics for Fig. 1c-i and Supplementary Fig. 3.

File name: Supplementary Data 2
Description: Broad atlas regions, puncta counts, and statistics for cleared brain imaging, related to Fig. 2 and Supplementary Fig. 4.

File name: Supplementary Data 3
Description: Differential expression results for K27M vs control comparisons of pseudobulk profiles of scRNA celltypes at each age, related to Supplementary Figs. 8-9.

File name: Supplementary Data 4
Description: scRNA cell type markers and GSEA results for cell type-specific K27M vs control comparisons, related to Supplementary Fig. 8a, b and Supplementary Fig. 9a.

File name: Supplementary Data 5
Description: Results for scRNAseq K27M vs control condition test along pseudotime of the oligodendrocyte lineage, related to Supplementary Fig. 9b-c.

File name: Supplementary Data 6
Description: Robust cell type agnostic K27M signature derived from scRNA data and associated GO Biological Process term enrichments for signature, related to Supplementary Fig. 9d-f.

File name: Supplementary Data 7
Description: Bulk RNA-seq WGCNA module gene membership from Fig. 3g; Fig. 4c; and Supplementary Fig. 11a.

File name: Supplementary Data 8
Description: Differential expression results for bulk RNA-seq comparisons, related to Fig. 3j, k; Fig. 5e, f; Fig. 6e; and Supplementary Fig. 12e, f.

File name: Supplementary Data 9

St. Jude - Confidential

Description: Pre-ranked GSEA for bulk RNA-seq comparisons using genesets from published glioma datasets, related to Fig. 3h.

File name: Supplementary Data 10
Description: Hypergeometric enrichment analyses for bulk RNA-seq comparisons, related to Fig. 4a, b and Supplementary Fig. 13a-d.

File name: Supplementary Data 11
Description: Cell type deconvolution proportions and differentiation indices of differentiated OPC cultures using control brainstem scRNA as a reference, related to Fig. 4f; Fig. 5g; and Supplementary Fig. 8a-b.

File name: Supplementary Data 12
Description: Bivalently marked genes and enrichment results, related to Fig. 6a-e and Fig. 7b.
